# Transcriptional activation of plant immunity by salicylic acid

**DOI:** 10.1038/s41586-026-11021-5

**Published:** 2026-09-16

**Authors:** Songwen Zhang, Madeline Gish, Huan Li, Thomas R. Hinds, Haibin Mao, Moira Cornell, Massi Silvetti, Sydney Klucas, Clint Vorauer, Charlie C. Mundorff, Smita Yadav, Miklos Guttman, Ning Zheng

**Affiliations:** 1 Howard Hughes Medical Institute, University of Washington, Seattle, WA USA; 2 Department of Pharmacology, University of Washington, Seattle, WA USA; 3 Department of Medicinal Chemistry, University of Washington, Seattle, WA USA

**Keywords:** Plant hormones, Structural biology

## Abstract

The plant defence hormone salicylic acid (SA) triggers systemic acquired resistance by signalling through its receptor, NON-EXPRESSOR OF PATHOGENESIS-RELATED GENE 1 (NPR1), leading to widespread transcriptional reprogramming^1,2^. However, the molecular mechanism by which NPR1 senses SA and activates transcription remains unresolved. Here we show that SA stabilizes the SA-binding domain (SBD) of NPR1 and allosterically promotes its interaction with MED15A, a subunit of the Mediator complex. Leveraging the NPR1 proxiome and AlphaFold structural predictions, we identify a direct interaction between NPR1 and the kinase-inducible domain interacting (KIX) domain of MED15A. We show that recombinant NPR1 recruits MED15A in an SA-dependent manner, and that MED15A binding reciprocally enhances the affinity of NPR1 for SA. Cryo-electron microscopy and hydrogen–deuterium exchange mass spectrometry reveal that SA and the MED15A KIX domain cooperatively potentiate NPR1-SBD for ternary complex formation by stabilizing its SA-binding core via separate interfaces. We further demonstrate that NIMIN1, a repressor of the SA–NPR1 pathway, antagonizes SA signalling by competing with MED15A for the same NPR1-SBD docking site and allosterically blocking hormone binding. In line with previous genetic evidence establishing a critical role of MED15A in SA signalling, our findings provide a mechanistic resolution to the longstanding question of how SA promotes transcriptional activation during plant immune responses.

## Abstract (web summary)

A mechanistic basis for how the hormone salicylic acid promotes transcriptional activation during plant immune responses is described.

## Main

**Fig. 1:**
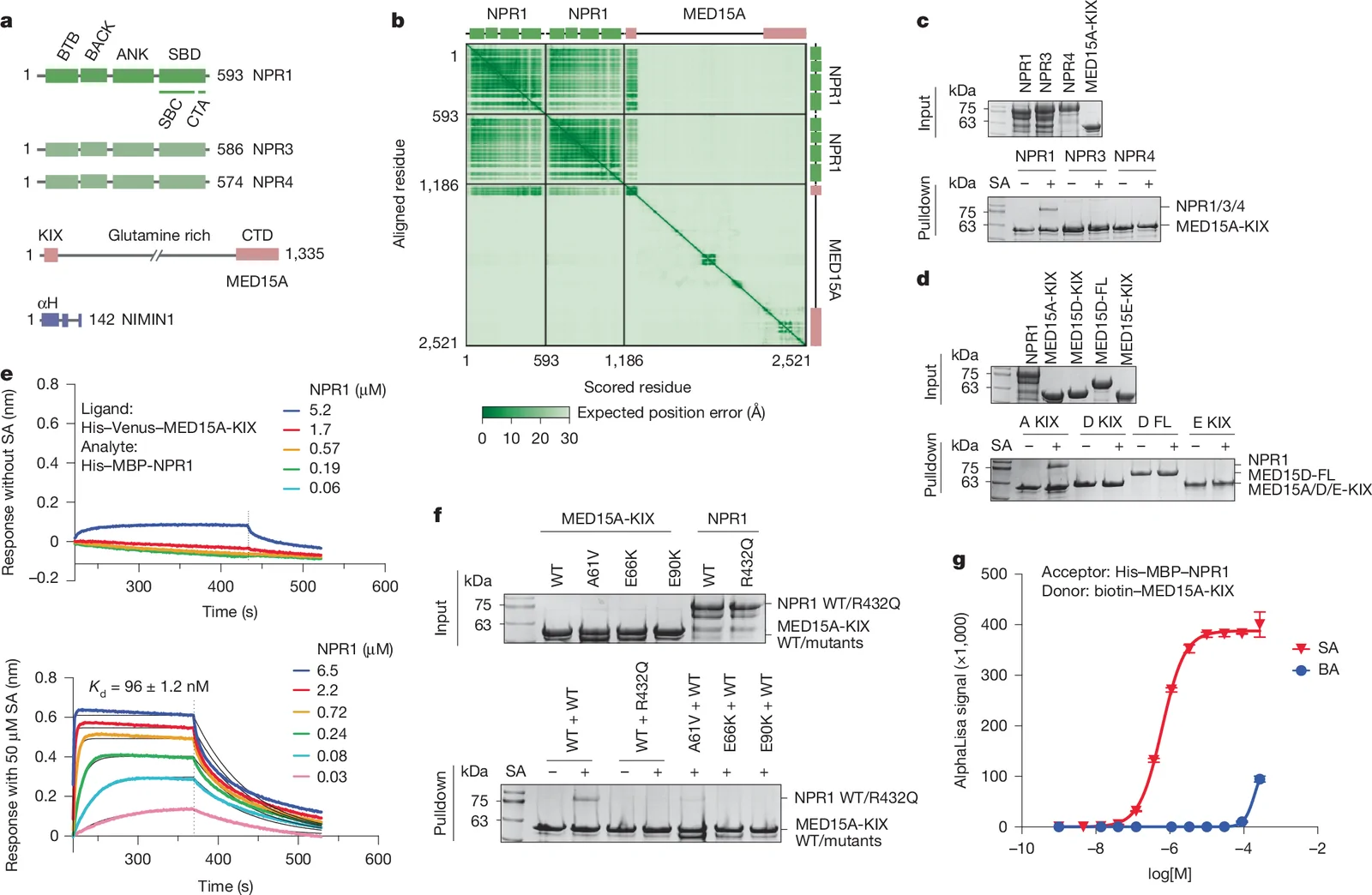
SA-dependent NPR1–MED15A-KIX interaction. **a**, Domain composition of *A. thaliana* NPR1, NPR3, NPR4, MED15A and NIMIN1. **b**, The AlphaFold predicted aligned error plot of the MED15A–NPR1 dimer interaction. **c**, Affinity pulldown assay assessing the SA-dependent binding of His–Venus-tagged NPR1, NPR3 and NPR4 to His–MBP-tagged MED15A-KIX. **d**, Affinity pulldown assay assessing the interaction between His–Venus-tagged NPR1 and the KIX domain of MED15 paralogues as well as the full-length MED15D (MED15D-FL) that are fused with His–MBP. **e**, Biolayer interferometry assay assessing the NPR1–MED15A-KIX interaction in the absence and presence of SA. His–Venus–MED15A-KIX was immobilized on Venus nanobody-coated streptavidin probes. The black curves are the fitting curves. The vertical dashed lines are the shift time points from association to dissociation. *K*_d_, dissociation constant. **f**, Affinity pulldown assay assessing the effect of mis-sense mutations on the NPR1–MED15A-KIX interaction. NPR1 wild type (WT) and R432Q were His–Venus tagged, and MED15A-KIX WT and mutants were His–MBP tagged. **g**, AlphaLISA assay assessing the activities of SA and benzoic acid (BA) in promoting the NPR1–MED15A-KIX interaction (*n* = 3 reactions). Data are presented as mean ± s.d. The solid lines represent non-linear regression fits (*R*^2^(SA) = 0.998 and *R*^2^(BA) = 0.997). The calculated EC_50_(SA) is 611.4 nM (95% confidence interval of 592.6–664.6 nM) and EC_50_(BA) is 449.4 µM (95% confidence interval of 431.2–468.5 µM). Experiments were performed three times (**c**–**g**). Source data

Facing constant challenges from pathogens, plants have evolved sophisticated immune responses to defend against these threats^3^. SA is a plant defence hormone that accumulates both locally and systemically in response to pathogen infection and has a critical role in establishing systemic acquired resistance^4–6^. At elevated levels, SA induces the expression of numerous *pathogenesis-related* (*PR*) genes, orchestrating a comprehensive transcriptional reprogramming that is essential for effective disease resistance. Early genetic studies in *Arabidopsis thaliana* have identified *NPR1* as the master positive regulator of SA-dependent immunity^7–9^. Loss-of-function *npr1* mutants are SA insensitive and fail to mount *PR* gene expression, thereby suffering from increased susceptibility to pathogens. Recent biochemical studies have revealed that NPR1, along with its paralogues NPR3 and NPR4, directly senses SA and functions as a hormone receptor^10–12^. At the sequence level, the NPR proteins share an N-terminal broad-complex, tramtrack and bric-à-brac (BTB) dimerization domain followed by a BTB and carboxyl-terminal Kelch (BACK) domain, a central TGA-binding ankyrin (ANK) repeat domain and a C-terminal SBD^13^ (Fig. 1a). Despite their high sequence homology, these SA receptors control transcription via assumed opposing mechanisms. Although NPR1 positively promotes SA-induced expression of defence genes, NPR3 and NPR4 act as transcriptional repressors, whose repressive activity is relieved upon SA binding^1,2^.

Despite recent advances in deciphering the SA signalling pathway, the precise mechanism by which SA-bound NPR1 activates transcription remains elusive. Although strong evidence has suggested that NPR1 is recruited to the promoters of *PR* genes through interaction with TGA (TGACG-binding) transcription factors, neither the NPR1–TGA interaction nor the recruitment of the complex to DNA appears to be SA-dependent^13,14^. This conundrum points to a hypothetic model in which SA might facilitate transcription reprogramming by enabling NPR1 to engage a yet-to-be-identified transcriptional co-regulator or chromatin modifier^1,7^. Such a SA-dependent NPR1-interacting protein might also help to explain the inconsistent SA-binding activity of NPR1, which varies substantially depending on the methods used for protein purification and ligand-binding measurement^10–12,15,16^. In stark contrast to NPR4, which exhibits robust SA binding with a low-nanomolar affinity, NPR1 may require a binding partner to potentiate its SA-binding activity, as other select phytohormone receptors^17,18^.

Here, by mining the proxiome of NPR1 using AlphaFold structural prediction, we identified MED15A, a subunit of the Mediator complex, as the long-sought factor that bridges SA-bound NPR1 to the general transcriptional machinery to drive *PR* gene expression. Through in-depth structural and biophysical analyses, we have elucidated the mechanism by which SA and MED15A mutually strengthen their interactions with NPR1 and how their cooperative function is antagonized by NIMIN1, a negative regulator of SA signalling.

## MED15A as a NPR1-binding candidate

**Extended Data Fig. 1:**
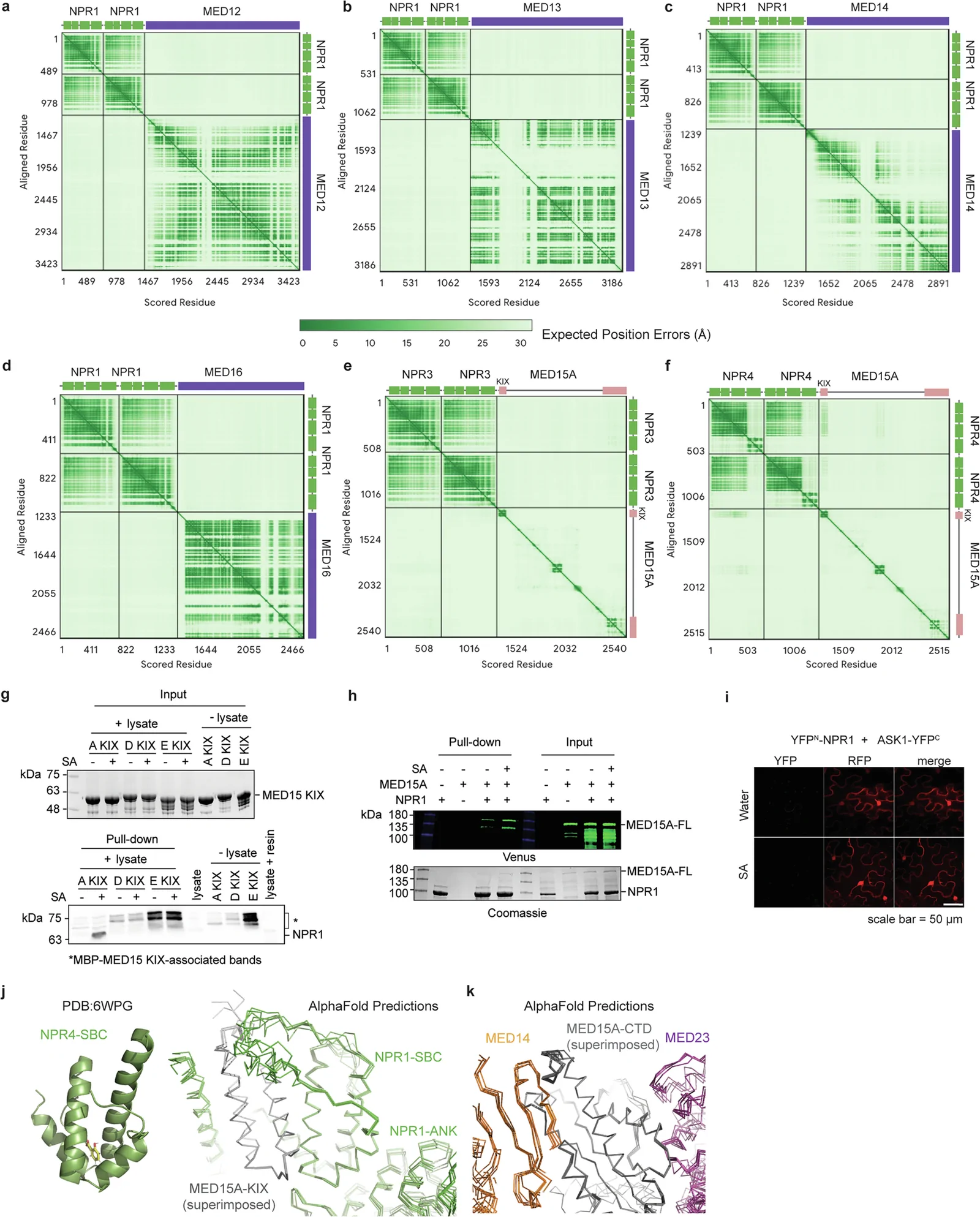
Assessment of potential interactions between NPR protein and select Mediator subunits. **a–d**. The AlphaFold PAE plots of the predicted interactions between *Arabidopsis thaliana* NPR1 dimer and MED12, MED13, MED14, and MED16. **e**,**f**. The AlphaFold PAE plot of the predicted interactions between *Arabidopsis thaliana* MED15A with NPR3 and NPR4 homodimers. **g**. Affinity pull-down of endogenous NPR1 from SA-treated *Arabidopsis thaliana* seedlings by His-MBP fused KIX domain of MED15 paralogues in the absence and presence of 200 µM SA. NPR1 was detected by western blot with an anti-NPR1 antibody. Asterisks indicate MED15-associated non-specific bands. **h**. Affinity pull down assay assessing the SA-sensitive interaction between Venus-MED15A^FL^ and MBP-NPR1. Venus-MED15A^FL^ was visualized by both Coomassie staining (bottom) and Venus fluorescence (top). **i**. Lack of interaction between co-expressed NPR1 and ASK1 as assessed by BiFC assay. The BiFC experiment here were performed once. **j**. Interaction between MED15A-KIX and NPR1 predicted by AlphaFold. Left: crystal structure of SA-bound NPR4-SBC. Right: superposition analysis of five MED15A^KIX^-NPR1 complex solutions predicted by AlphaFold with only MED15A-KIX superimposed. The converging solutions of NPR1-SBC indicate high confidence of the predictions. **k**. A close-up view of the interactions among *Arabidopsis thaliana* MED15A C-terminal domain (CTD), MED14 and MED23 in five solutions predicted by AlphaFold3 with only MED15A-CTD superimposed. The converging solutions of the MED15A-CTD contacting regions in MED14 and MED23 indicate high confidence of the predictions. Experiments in **g** and **h** were repeated three times.

To identify the missing factor that hypothetically interacts with SA-bound NPR1, we performed AlphaFold predictions of the SA receptor in complexes with individual NPR1 proximal proteins recently revealed by proximity labelling experiments^19^. In addition to NIMIN1, which has been shown to directly interact with NPR1 (refs. ^20,21^), a subunit of the Mediator complex, MED15A, emerged as a strong candidate based on several lines of evidence. First, the N-terminal KIX domain of MED15A is predicted to interact with NPR1 with high confidence scores (Fig. 1b), whereas other Mediator subunits identified in the NPR1 proxiome, including MED12, MED13, MED14 and MED16, are not predicted to bind to the SA receptor (Extended Data Fig. 1a–d). Second, the predicted aligned error values are markedly lower for the interaction between MED15A-KIX and NPR1 than with NPR3 or NPR4, hinting at a high specificity of their interface (Extended Data Fig. 1e,f). Third, and most importantly, the *Non-Recognition-of-BTH4* (*NRB4*) gene, which encodes MED15A, has been previously identified in an unbiased genetic screen as having a critical role in SA response^22^. All three *nrb4* alleles recovered from the screen harbour missense mutations clustered in the KIX domain of MED15A. With SA-insensitive phenotypes resembling *npr1* mutants, these *nrb4* mutants show no defects in other tested phenotypes. The multi-subunit Mediator complex functions as a crucial bridge between transcription factors and RNA polymerase II during transcription activation^23,24^. As a subunit of the Mediator Tail module, MED15 directly interacts with various transcription factors through its KIX domain in yeast, plants and animals^25–27^. Our AlphaFold predictions strongly suggest that *A. thaliana* MED15A may physically link NPR1 with the Mediator complex to drive RNA polymerase II-dependent transcription.

## SA-dependent NPR1–MED15A interaction

Although yeast two-hybrid assays have previously failed to detect any MED15A–NPR1 interaction either in the absence or presence of SA^22^, we sought to validate the AlphaFold predictions using purified recombinant proteins. In vitro pulldown assays revealed a robust SA-dependent interaction between the KIX domain of MED15A and NPR1 (Fig. 1c). Consistent with the AlphaFold predictions, MED15A-KIX showed no detectable interaction with NPR3 or NPR4 (Fig. 1c). Beyond MED15A, the *A. thaliana* genome encodes two additional KIX domain-containing MED15 paralogues: MED15E and MED15D. Although MED15E shares greater sequence similarity with MED15A, MED15D harbours two tandem KIX domains, the first of which is more homologous to MED15A-KIX^28^. Despite these similarities, MED15E and MED15D showed little to no interaction with NPR1 in the presence or absence of SA (Fig. 1d). The interaction between NPR1 and the KIX domain of MED15A, therefore, is highly specific and reflects the distinct functions of the NPR proteins as well as the non-redundant roles of the MED15 paralogues in SA signalling. Using biolayer interferometry, we next quantified the binding between NPR1 and MED15A-KIX. As expected, SA had a profound effect on the NPR1–MED15A interaction, increasing their binding affinity from a marginally detectable level to approximately 100 nM (Fig. 1e).

To further confirm their SA-dependent interaction, we introduced SA-insensitive missense mutations in NPR1 and MED15A-KIX, including the *npr1-9* allele (R432Q) and the three *nrb4* alleles (A61V, E66K and E90K) characterized from previous genetic screens^8,11,22,29^. All four single amino acid mutations either abolished or severely impaired the NPR1–MED15A-KIX interactions in the in vitro pulldown assay (Fig. 1f). By monitoring the binding between the two proteins in an amplified luminescent proximity homogenous assay (ALPHA), we established a dose–response curve for SA with a half-maximal effective concentration (EC_50_) value of approximately 600 nM (Fig. 1g). By contrast, benzoic acid, an inactive SA analogue lacking the *ortho*-hydroxyl group, could barely promote the NPR1–MED15A interaction even at a concentration of 0.5 mM. Collectively, these in vitro results indicate that SA-primed NPR1 can specifically engage MED15A, a Mediator subunit crucial for the SA signalling pathway.

**Fig. 2:**
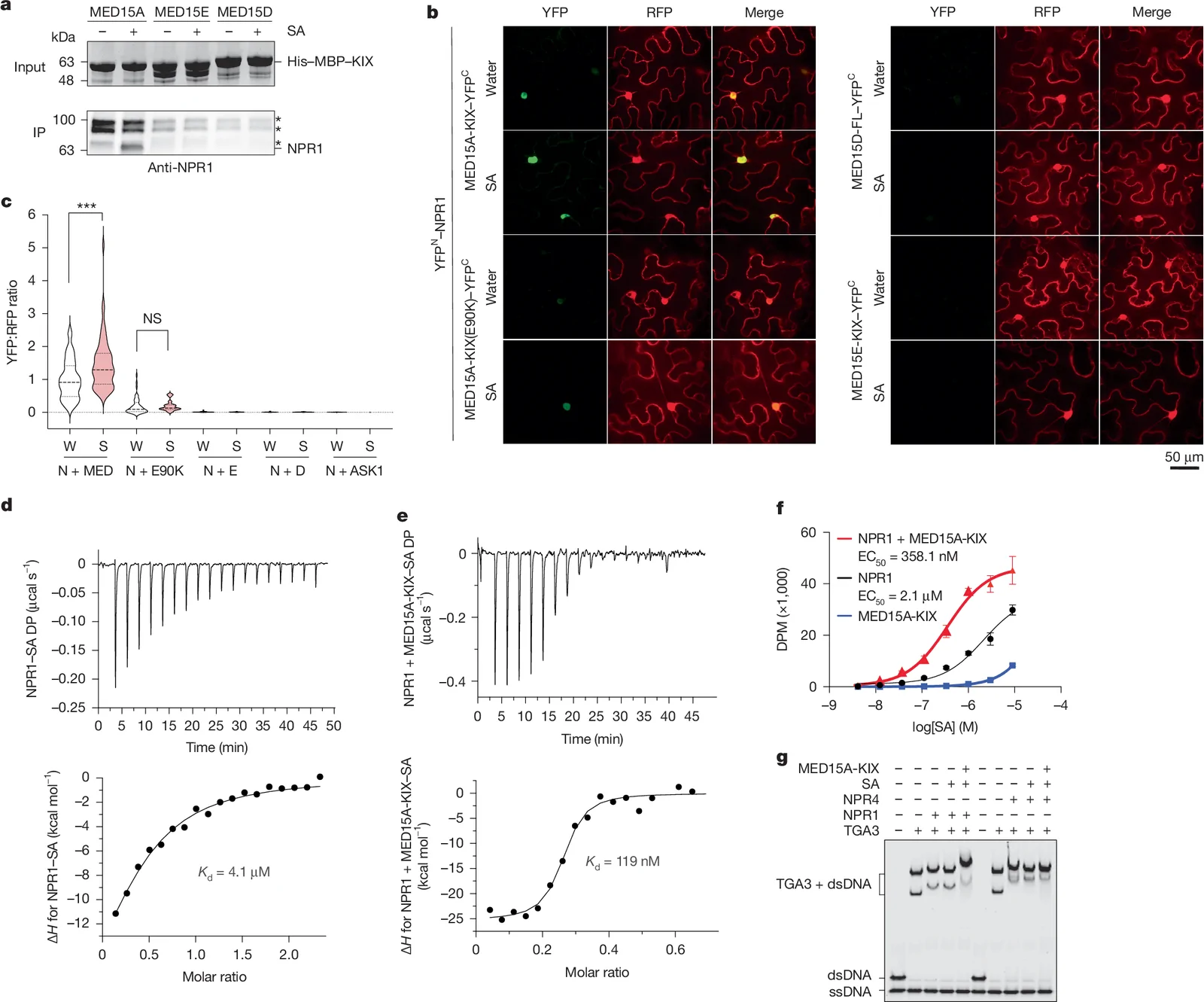
Cooperativity of SA and MED15A for NPR1 binding. **a**, Affinity pulldown of endogenous NPR1 from SA-treated *A. thaliana* seedlings by His–MBP-fused KIX domain of MED15 paralogues in the absence and presence of 200 µM SA. NPR1 was detected by western blot with an anti-NPR1 antibody. The asterisks indicate MED15-associated nonspecific bands. IP, immunoprecipitate. **b**, BiFC assays addressing the in vivo interaction between YFP^N^-fused NPR1 (N) and YFP^C^-fused WT MED15A-KIX (MED), MED15A-KIX(E90K), MED15E-KIX (E), MED15D-FL (D), and a known non-interactor, ASK1, transiently expressed in tobacco. RFP is an internal marker. The *Agrobacterium*-infiltrated tobacco leaves were infiltrated again with either 10 mM MgCl_2_ (water) or 10 mM MgCl_2_ with 0.5 mM SA at 2 days post-infiltration. Leaves were imaged at 5 h after treatments. The fluorescence images for the ASK1 control are in Extended Data Fig. 1i. **c**, Statistical analysis of the normalized YFP:RFP ratio. The dashed lines are the lower quartile, median and upper quartile, respectively, from bottom to top. *P* values were calculated using two-tailed Welch’s *t*-test (*n* > 40 nuclei for ‘N + MED’, *n* > 30 nuclei for ‘N + E90K’, *n* > 20 nuclei for the rest): ***P = 0.0003; not significant (NS) *P* = 0.99. S, SA; W, water. **d**, Binding affinity of SA to NPR1 determined by ITC. SA was titrated into NPR1. DP, differential power; Δ*H*, enthalpy change. **e**, Cooperative binding of SA and MED15A-KIX to NPR1 determined by ITC. SA was titrated into NPR1 + MED15A-KIX. **f**, Affinity of SA towards NPR1 in the absence and presence of MED15A-KIX determined by radio-labelled ligand-binding assay using ^3^H-labelled SA. MED15A-KIX was used as a negative control. Data are presented as mean ± s.d. (*n* = 3 reactions). DPM, disintegrations per minute. **g**, Assembly of the *as-1*-anchored TGA3–MED15A complex by NPR1, but not by NPR4, in the presence of SA as assessed by an electrophoresis mobility shift assay. dsDNA, double-stranded DNA; ssDNA, single-stranded DNA. Experiments were performed three times (**a**,**d**–**g**). Source data

To rule out any artefact introduced by recombinant NPR1, we next verified that the wild-type KIX domain of MED15A, but not that of MED15D or MED15E, can pull down endogenous NPR1 from the *Arabidopsis* leaf lysate in a SA-dependent manner (Fig. 2a and Extended Data Fig. 1g). Moreover, NPR1 also showed SA-sensitive binding to full-length MED15A purified from insect cells (Extended Data Fig. 1h). In a bimolecular fluorescence complementation (BiFC) assay, we further validated that SA treatment markedly enhances the interaction between NPR1 and MED15A-KIX fused with complementary fragments of yellow fluorescent protein (YFP; Fig. 2b,c). Such an effect was abolished when MED15A-KIX was replaced with the E90K mutant, the KIX domain of MED15D or MED15E, or an unrelated protein, ASK1 (Fig. 2b,c and Extended Data Fig. 1i). Together, these findings support the notion that SA promotes the direct and specific engagement between NPR1 and the KIX domain of MED15A in vivo.

## MED15A sensitizes NPR1 to bind to SA

Our previous work has revealed that NPR4 recognizes the ligand through a four-helical SA-binding core (SBC) within its SBD, a structure stabilized by the ligand itself^15^. Although NPR1 possesses identical SA-contacting residues, uncertainty in its ligand-binding activity complicates a definitive understanding of its role as a SA receptor^10–12,15^. The AlphaFold model places the KIX domain of MED15A adjacent to the SBC of NPR1, suggesting that MED15A may stabilize the four-helix bundle to strengthen ligand engagement (Extended Data Fig. 1j). To test this idea, we monitored the binding between NPR1 and SA using isothermal titration calorimetry (ITC) in the presence and absence of MED15A-KIX. Without MED15A-KIX, NPR1 recognizes SA with a low-micromolar affinity (Fig. 2d). Inclusion of MED15A-KIX enhanced the ‘apparent SA-binding affinity’ of NPR1 to approximately 119 nM, which reflects the cooperative engagement of the ligand and the binding partner with the hormone receptor (Fig. 2e). Using a membrane filter assay with ^3^H-labelled SA, we further confirmed that NPR1 gains a substantially higher affinity specifically towards SA when mixed with MED15A-KIX (Fig. 2f). Together, these results corroborate the AlphaFold prediction model and demonstrate that recruitment of MED15A-KIX by SA-bound NPR1 further enhances its SA sensitivity, implicating the cooperative nature of the tri-molecular complex formation.

## SA-bound NPR1 links Mediator to TGA–DNA

The NPR1 homodimer has been reported to engage a pair of TGA3 dimers anchored to the *PR1* gene promoter DNA with two *as-1* *cis*-elements^13^. The TGA-binding site was mapped to the tip of the NPR1 ANK domain, distal to its SBD. This structural arrangement predicts that the NPR1–TGA–DNA assembly is compatible with MED15A-KIX binding. In an electrophoresis mobility shift assay, both NPR1 and NPR4 produced a super-shift of the TGA3–DNA complex in the absence of SA. However, only the NPR1 super-shift was further upshifted upon addition of SA and the KIX domain of MED15A (Fig. 2g). We therefore conclude that SA-bound NPR1 can bridge MED15A and DNA-bound TGA3.

In addition to its N-terminal KIX domain, MED15A contains a C-terminal structured domain (CTD), which shares sequence homology with its counterpart in human MED15. Cryo-electron microscopy (cryo-EM) studies of the human Mediator complex have established the MED15 CTD as a core component of the Tail module, forming direct contacts with MED14 and MED23 (ref. ^30^). AlphaFold predictions indicate that the *Arabidopsis* MED15A CTD engages in analogous interactions with its partners, MED14 and MED23 (Extended Data Fig. 1k). A key feature of MED15A is the more than 1,000-amino acid-long linker region between its two terminal domains, which is glutamine rich and predicted to be largely disordered (Fig. 1a). It is conceivable that this linker sequence allows NPR1 to recruit the entire Mediator complex by binding to the MED15A KIX domain without any steric hindrance. Supporting this mechanism, four additional Mediator subunits (MED12, MED13, MED14 and MED16) have been identified as NPR1-proximal proteins^19^, and mutations in three Mediator subunits (MED14, MED16 and MED19a) besides MED15A have been shown to impair SA responses^31–34^. Together, these data support a model in which SA-activated NPR1 drives transcription by serving as a molecular scaffold that recruits the Mediator complex to the promoter-bound TGA factors.

## SA–NPR1–MED15A complex structure

**Fig. 3:**
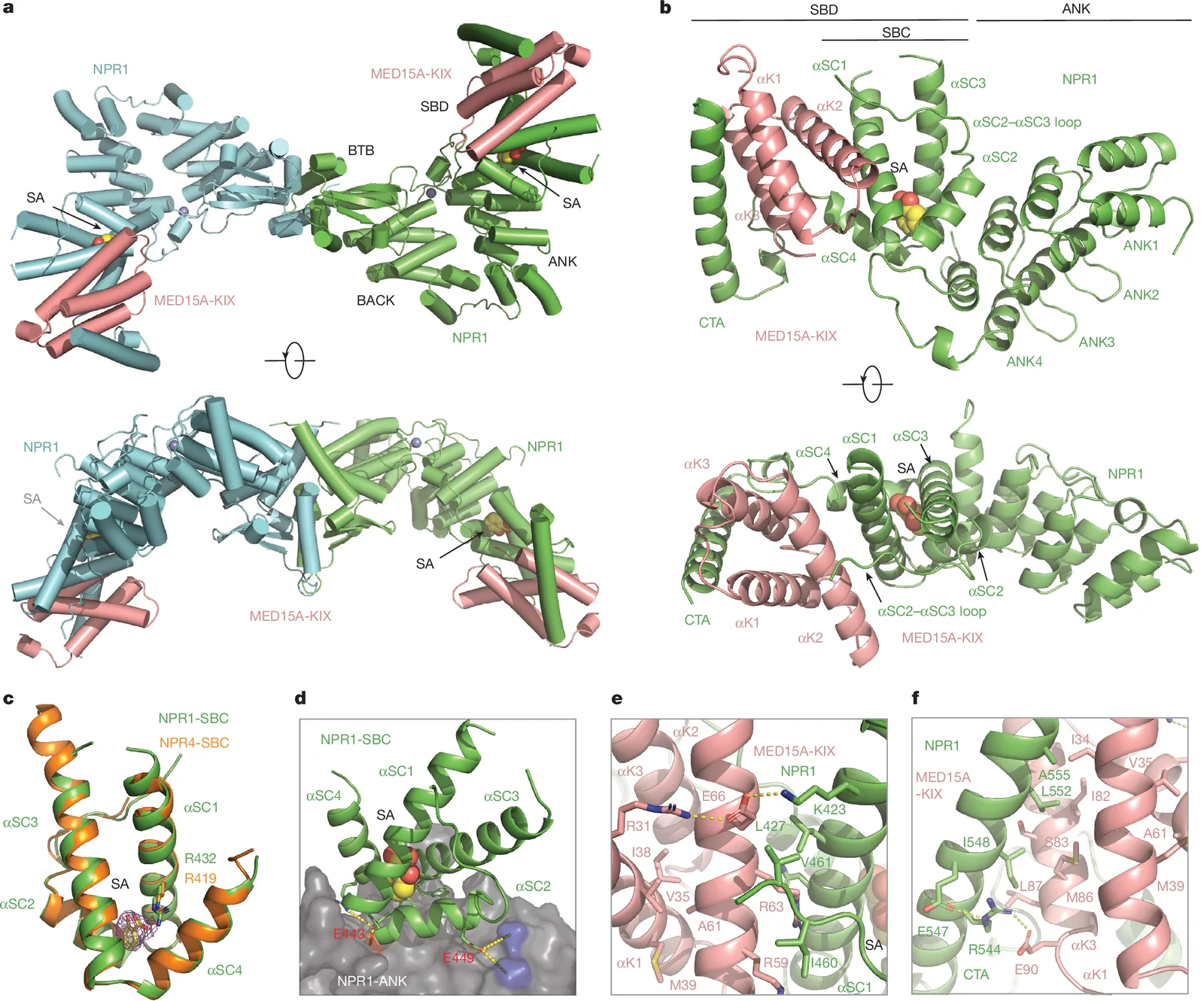
Cryo-EM structure of NPR1–SA–MED15A-KIX. **a**, Two orthogonal views of a MED15A-KIX-bound NPR1 dimer modelled based on the NPR1 dimer and the NPR1–MED15A-KIX protomer structures. **b**, Two orthogonal views of the NPR1-ANK–SBD–MED15A-KIX structural module. SA is shown in red–yellow spheres. The α-helices of the NPR1-SBD and MED15A-KIX are labelled. **c**, Superposition of the SA-bound NPR1-SBC and NPR4-SBC. SA bound to NPR1 is shown as sticks, with its density shown as mesh. **d**, A close-up view of the interaction between SA-bound NPR1-SBC and NPR1-ANK. SA is shown in red–yellow spheres. The side chains of two SBC glutamate residues mutated in SA-insensitive *npr1* alleles are shown as red–green sticks. The yellow dashed lines and blue-and-red-coloured NPR1-ANK surface areas indicate a polar interaction formed between the two glutamate residues and amino acids in NPR1-ANK. **e**, A close-up view of the interface between the MED15A-KIX αK2 helix and the NPR1 αSC1 helix. The dashed lines indicate polar interactions. The side chains of interfacial residues and MED15-KIX amino acids contributing to its hydrophobic core are shown as sticks. **f**, A close-up view of the interface between the NPR1 CTA helix and the αK1 and αK3 helices of MED15A-KIX and the MED15A-KIX residues surrounding Ala61 (see also panel **e**).

**Extended Data Fig. 2:**
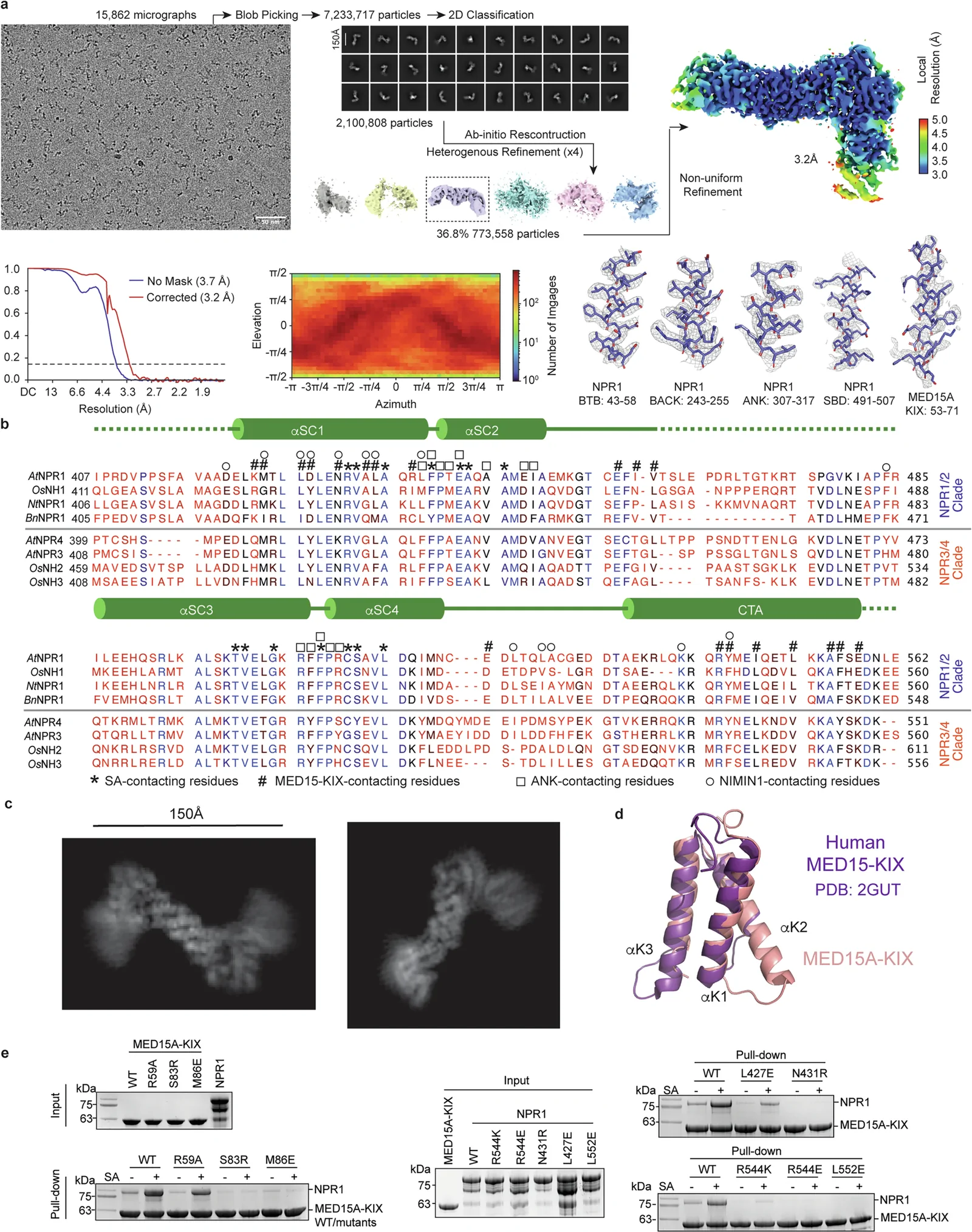
Cryo-EM structure determination of NPR1-SA-MED15A^KIX^ complex. **a**. A simplified cryo-EM data processing workflow for the NPR1-SA-MED15A^KIX^ complex. One presentative micrograph out of 15,862 is shown here. **b**. Structure-based sequence alignment of the SBD regions of NPR1 and NPR4 orthologues. The secondary-structure diagram of NPR1 SBD is shown above the sequences. Regions with no regular secondary structure are shown by lines, and α-helices are represented by cylinders. The dashed lines indicate a disordered loop that is not resolved in the structure. Strictly conserved residues are coloured in blue. The rest of the sequences are coloured in black, brown, and red based on their decreasing degrees of conservation. The residues directly involved in SA, MED15A-KIX, ANK, and NIMIN1 binding are indicated by asterisk, hash, black square, and black circle signs. *At* (*Arabidopsis thaliana*; *NPR1*-AT1G64280, *NPR3*-AT5G45110, and *NPR4*-AT4G19660); *Os* (*Oryza sativa NH1*-Os01g09800, *NH2*-Os01g56200, and *NH3*-Os03g46440); *Nb (Nicotiana benthamiana NPR1*- LOC107831756); *Bn*, (*Brassica napa NPR1*- LOC106389246). **c**. Representative 2D class averages showing the symmetric nature of the dimeric NPR1-SA-MED15A^KIX^ complex. **d**. Superposition analysis of *Arabidopsis thaliana* MED15A KIX domain (salmon) and its human ortholog (magenta, Protein Data Bank ID: 2GUT). **e**. Maltose affinity pull-down assays assessing the impact of select NPR1-MED15A^KIX^ interface residues on NPR1-MED15A^KIX^ interactions. Experiments in **e** was repeated three times.

To elucidate the structural basis of the SA-dependent interaction between NPR1 and MED15A, we next determined the cryo-EM structure of the SA-bound NPR1 in complex with the MED15A KIX domain at a 3.2 Å resolution (Fig. 3a, Extended Data Table 1 and Extended Data Fig. 2a,b). Two-dimensional class averages of the SA–NPR1–MED15A-KIX particles revealed a symmetrical dimeric complex with discernible densities of NPR1-SBD and MED15-KIX (Extended Data Fig. 2c). To improve the resolution of the NPR1–MED15A interfaces, we performed non-uniform global refinement focusing on a single protomer. Consistent with the AlphaFold predictions, the MED15A-KIX domain binds exclusively to NPR1-SBD, which itself is fully engaged with the ANK domain of the SA receptor (Fig. 3a,b).

The complex structure revealed an unexpected architecture of NPR1-SBD, which consists of two independent structural elements. The majority of NPR1-SBD forms the four-helix bundle SBC, which houses the SA-binding site constructed by four SA-contacting helices: αSC1 to αSC4 (Fig. 3b). The rest of NPR1-SBD folds into a C-terminal helical arm (CTA) that is detached from SBC but acts together to bind to MED15A-KIX. Akin to its binding mode with NPR4-SBC^15^, SA is embedded within the centre of the NPR1-SBC four-helix bundle. Superposition analysis reveals that SA-bound NPR1-SBC and NPR4-SBC share a nearly identical topology with strictly conserved SA-contacting residues (Fig. 3c and Extended Data Fig. 2b). Outside the SA-binding pocket, however, NPR1-SBC and NPR4-SBC show marked sequence divergence, which is consistent with their differential capacities for binding MED15A and the assumed opposite mechanisms of their functions (Extended Data Fig. 2b).

In agreement with a previous model of NPR1-SBC^13^, the SA-occupied four-helix bundle is stably anchored to the NPR1 ANK domain (Fig. 3b). With the help of a long intra-repeat loop in the fourth ankyrin repeat (ANK4), NPR1-ANK presents a platform that cradles the SBC αSC2 helix while enclosing the short αSC1–αSC2 and αSC3–αSC4 loops (Fig. 3d). In addition to the previously characterized hydrophobic interactions, the SBC–ANK interface is further reinforced by multiple polar residues, including Glu443 and Glu449, whose missense mutations underlie the SA-insensitive *npr1-25* and *npr1-31* alleles^29^. Although SA is completely sequestered by the SBC and makes no direct contact with the ANK domain, the extensive SBC–ANK interface strongly suggests that the ANK domain contributes to the stabilization of the SBC by SA.

## NPR1-SBD–MED15A-KIX interface

The MED15A KIX domain adopts a three-helix bundle structure comprising three α-helices designated αK1, αK2 and αK3 (Fig. 3b and Extended Data Fig. 2d). Unlike previously characterized mammalian KIX domains^25,35,36^, which utilize a localized surface to bind transcription factor activation domains, MED15A-KIX interacts with NPR1-SBD through a bipartite interface that involves all three α-helices. On one side of the KIX domain, the αK2 helix packs against the NPR1-SBC αSC1 helix at an approximately 40° angle. Their interface is augmented by the partially ordered loop linking αSC2 and αSC3 as well as the C-terminal end of αSC4, which make contacts with both the αK2 and the αK3 helices of MED15A-KIX. On the opposing side, the NPR1 CTA helix is simultaneously engaged with the αK1 and αK3 helices of MED15A-KIX. Together, the NPR1 SBC and CTA helix sandwich the KIX domain of MED15A from opposite sides and bury a surface area of approximately 1,740 Å^2^.

Reminiscent of the NPR1 ANK–SBC interface, the SA-dependent interaction between MED15A-KIX and NPR1-SBD is mediated by an extensive network of hydrophobic and polar contacts. These include two MED15A residues, whose missense mutations were previously identified in the SA-insensitive *nrb4* alleles^22^. Specifically, Glu66 of MED15A-KIX extends the hydrophobic αK2–αSC1 helix packing by forming a salt bridge with the NPR1 Lys423 residue (Fig. 3e). Its mutation to lysine, E66K, found in the *nrb4-3* allele, is expected to not only disrupt the salt bridge but also introduce electrostatic repulsion at the inter-molecular junction. Similarly, the E90K mutation by the *nrb4-2* allele would impact the salt bridge formed between Glu90 of MED15A-KIX and Arg544 of NPR1-CTA at the edge of the αK1–αK3–CTA interface (Fig. 3f). The same arginine residue on the NPR1 side of this salt bridge is mutated to lysine in the *npr1-22* allele^29^. Despite maintaining a positive charge, the resulting R544K mutation abrogates the bidentate interactions that Arg544 simultaneously makes with its nearby Glu547 and MED15A Glu90 residues. Of note, the third *nrb4* allele (*nrb4-1*) is characterized by an A61V mutation^22^, which introduces two extra methyl groups into the hydrophobic core of the KIX domain formed among Ala61 and three surrounding hydrophobic residues (Val35, Ile38 and Met39; Fig. 3e,f). The resulting bulkier side chain is expected to compromise the proper folding of the compact three-helix bundle. Its SA-insensitive phenotype, therefore, underscores the functional importance of the structural integrity of the KIX domain of MED15A. Analogous to these genetically mapped interfacial residues, mutations of additional amino acids at the NPR1-SBD–MED15A-KIX interfaces either weakened or abrogated their interactions in affinity pulldown assays (Extended Data Fig. 2e). Overall, MED15A-KIX, SA-bound NPR1-SBD and the NPR1-ANK domain appear to form a continuous and closely packed all-helical assembly, which is nucleated by the hormone at the centre.

## Cooperative action of SA and MED15A

**Fig. 4:**
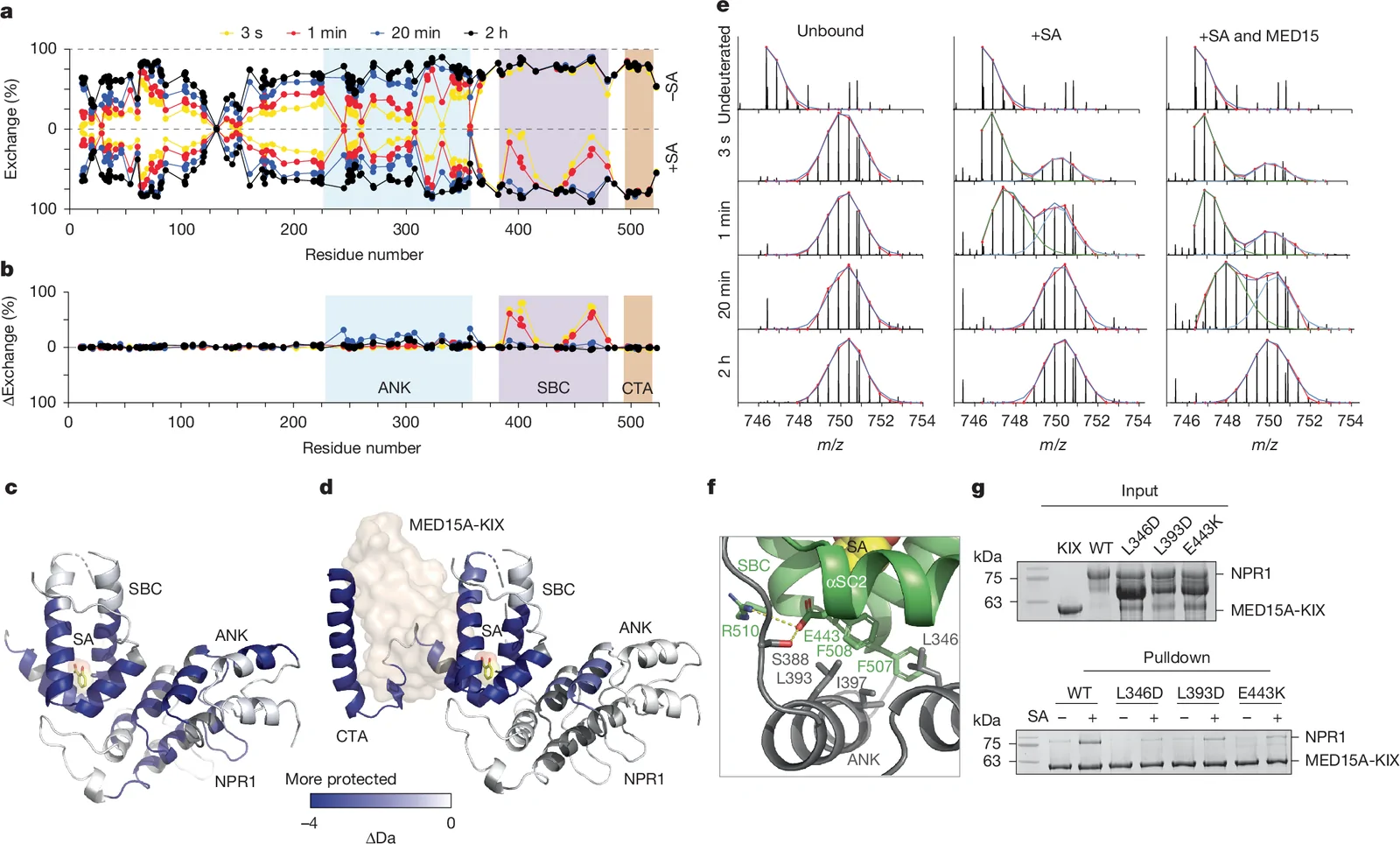
Stabilization of NPR1-SBD by SA and MED15A-KIX. **a**, Mirror plot showing the percentage of deuterium exchange after 3 s (yellow), 1 min (red), 20 min (blue) and 2 h (black) for each observable peptide at the midpoint of their primary sequence for the apo (top) and SA-bound (bottom) NPR1. Dashed lines represent 0% and 100% of deuterium exchange. **b**, Net difference (SA-free − SA-treated) in the percentage deuterium exchange at each time point plotted for each observable peptide. Regions with slowed exchange upon SA binding fall in the positive *y* axis and are highlighted. The ANK domain, the SBC and the CTA helix regions are highlighted in cyan, light purple and light brown colours, respectively. **c**, Structural mapping of SA-induced differences in HDX of the NPR1 ANK domain and the SBC. The structural model is based on the cryo-EM structure of the NPR1–SA–MED15A-KIX complex. Regions in blue are protected upon SA binding, with a colour ramp reflecting the degree of protection calculated as the summation of changes across all four time points. SA is shown as red–yellow sticks with its transparent surface. **d**, Structural mapping of SA-induced differences in HDX of the NPR1 ANK domain and the SBD. MED15A-KIX is shown in yellow surface representation. **e**, Bimodal exchange behaviour of a NPR1-SBC peptide in the free, SA-bound and SA–MED15A-bound forms of NPR1. Individual populations within the spectra are outlined in green and cyan. The amino acid sequence of the peptide is QRLFPTEAQAAME (amino acids 398–410). **f**, A close-up view of the SBC–ANK interface with the side chains of key hydrophobic and polar interfacial residues shown as sticks. **g**, Affinity pulldown assay assessing the impact of ANK (L346D and L393D) and SBC (E443K) missense mutations at the SBC–ANK interface and found in the SA-insensitive *npr1* alleles to the SA-dependent NPR1–MED15A-KIX interaction. The experiment was performed three times.

**Extended Data Fig. 3:**
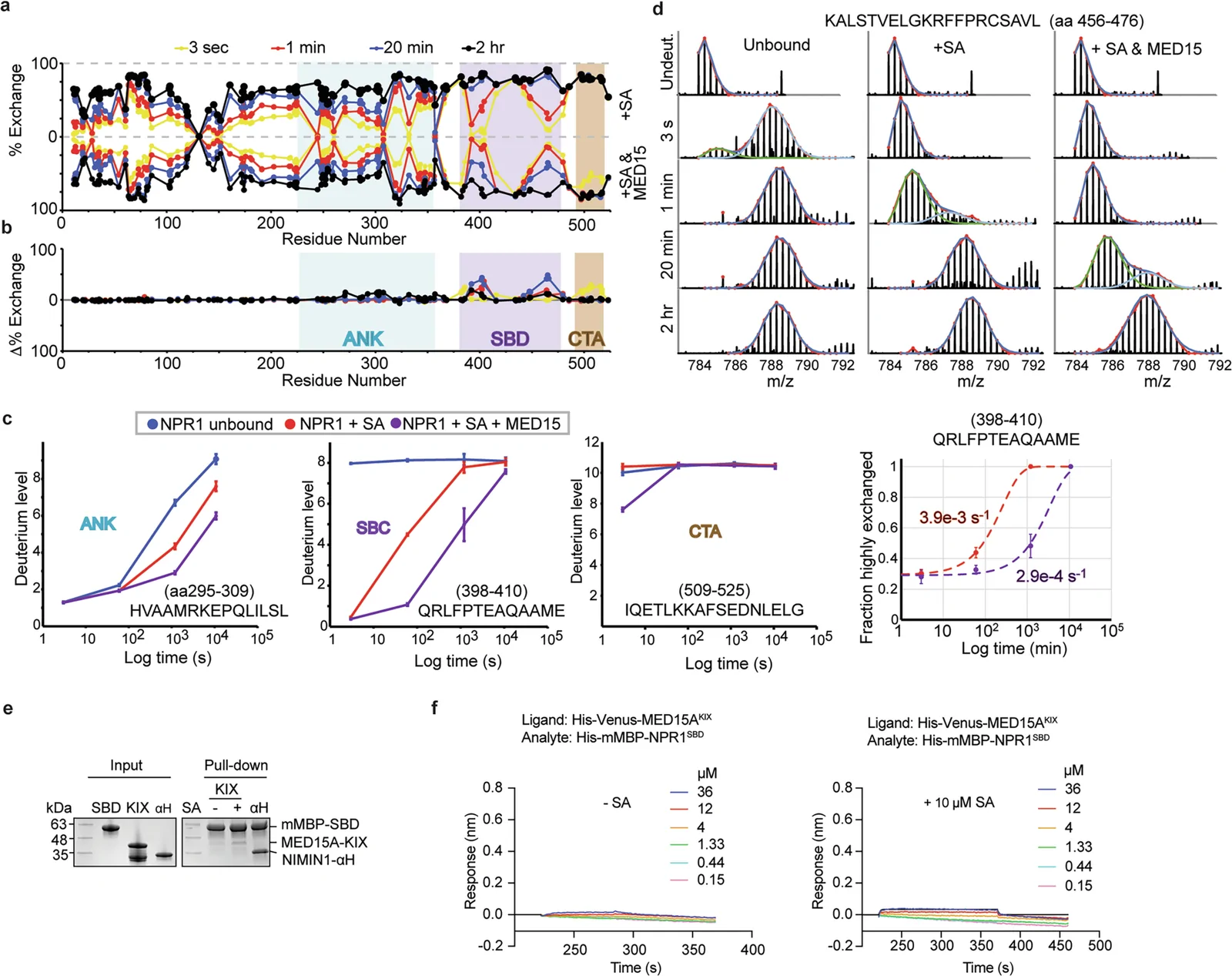
HDX-MS and BLI analysis of NPR1-SA-MED15A^KIX^ interactions. **a**. Mirror plots showing the percentage of deuterium exchange after 3 s (yellow), 1 min (red), 20 min (blue) and 2 h (black) for each observable peptide at the midpoint of their primary sequence for the SA-bound (top) and SA- and MED15A-KIX-bound (bottom) NPR1. **b**. Net difference (SA-bound vs. SA- and MED15A-KIX-bound) in the percentage deuterium exchange at each time point plotted for each observable peptide. Regions with slowed exchange upon MED15A-KIX binding fall in the positive *y*-axis. The ANK domain, the SBC, and the C-terminal arm (CTA) helix regions are highlighted in cyan, light purple, and light brown colors. **c**. Deuterium uptake plots of three representative peptides of the NPR1 ANK domain, SBC, and the C-terminal arm in the absence or presence of SA and MED15A-KIX. n = 3 independent samples. Error bars representing s.d. (center value) are shown. The peptide sequences, amino acid numbers, and structural domain to which they belong are indicated. Panel on the right shows the plot of the protected and highly exchanged populations of peptide 398-410 in the +SA (red) and +SA & MED15 states (purple). Dashed lines show fits of a single exponential with rates in the inset. **d**. Bimodal exchange behavior of a NPR1-SBC peptide (residues 456–476) in the free, SA-bound, and SA-MED15A-bound forms of NPR1. Individual populations within the spectra are outlined in green and cyan. The amino acid sequence of the peptide is shown above the plots. **e**. Affinity pull-down assay showing that NPR1-SBD is sufficient for NIMIN1 binding but not for SA-dependent MED15A-KIX binding (NIMIN1-αH long, residues 31-72). **f**. Lack of detectable interactions between isolated NPR1-SBD and MED15A-KIX in the absence or presence of SA as determined by BLI. The concentration of His-mMBP-NPR1^SBD^ as an analyte was titrated from 150 nM to 36 μM. Experiments in **e** and **f** were repeated three times. Source data

To further elucidate the mechanism by which SA promotes the NPR1–MED15A interaction, we performed hydrogen–deuterium exchange mass spectrometry (HDX-MS) analyses to assess the effect of SA and MED15-KIX on NPR1. HDX-MS measures the conformational stability of hydrogen bonds across a protein as stable hydrogen bonds exchange slowly, whereas unstructured or backbone amides in flexible regions exchange quickly. Similar to NPR4 (ref. ^15^), the structure of NPR1-SBC in the free NPR1 sample is intrinsically flexible, as evidenced by its rapid deuterium exchange rate compared with the rest of the protein (Fig. 4a and Supplementary Table 1). Consistent with previous studies, SA binding not only stabilized the SBC but also reduced deuterium uptake in a series of sequences within the ANK domain, corroborating SA-mediated SBC–ANK engagement (Fig. 4b,c). Of note, the addition of MED15-KIX to SA-bound NPR1 induced further protection of NPR1-SBD, including both the SBC and the CTA helix (Fig. 4d and Extended Data Fig. 3a–c). The peptides spanning the helices nesting the SA in the SBC display a unique bimodal exchange profile in the SA-bound and SA–MED15A-bound states (Fig. 4e and Extended Data Fig. 3d). This correlated population shift to a fast-exchanging state probably results from a large-scale unfolding of the SBC helices during a transient dissociation of the bound SA. The apparent unfolding rate is approximately tenfold slower with MED15A-KIX bound (Extended Data Fig. 3c), further indicating that the MED15A interaction stabilizes the helices within the SBD. Together, these results strongly suggest that SA potentiates NPR1-SBD for binding MED15A-KIX by structurally organizing the SBC four-helix bundle. The KIX domain of MED15A, in return, enhances the structural stability of the SA-bound SBC, thereby strengthening the engagement of the hormone. Rigidification of the SBC four-helix bundle, therefore, represents a common mechanism underlying the SA-induced NPR1–MED15A-KIX interaction and the MED15A-KIX-enhanced affinity of NPR1 for SA.

Previous mutational studies have established a critical role of the SBC–ANK interface in the function of NPR1 as a SA receptor^13^. The propagation of the SA-induced and MED15A-KIX-induced structure stabilization effects to the ANK domain implicates that the cooperative action between SA and MED15A-KIX is most likely shared by the NPR1 ANK domain (Fig. 3b–d and Extended Data Fig. 3b). Indeed, isolated NPR1-SBD could not effectively interact with MED15A-KIX in the presence of SA when the ANK domain was absent (Extended Data Fig. 3e,f). Similarly, SA-dependent interaction between full-length NPR1 and MED15A-KIX was either abolished or appreciably compromised when missense mutations disrupting the hydrophobic packing (L346D and L393D) or polar interactions (E443K in the *npr1-25* allele) were introduced at the SBC–ANK interface (Fig. 4f,g). These findings demonstrate that the NPR1 ANK domain synergizes with SA to structurally stabilize the SBC four-helix bundle, thereby priming NPR1-SBD for binding the Mediator subunit.

## Competition between NIMIN1 and MED15A

**Extended Data Fig. 4:**
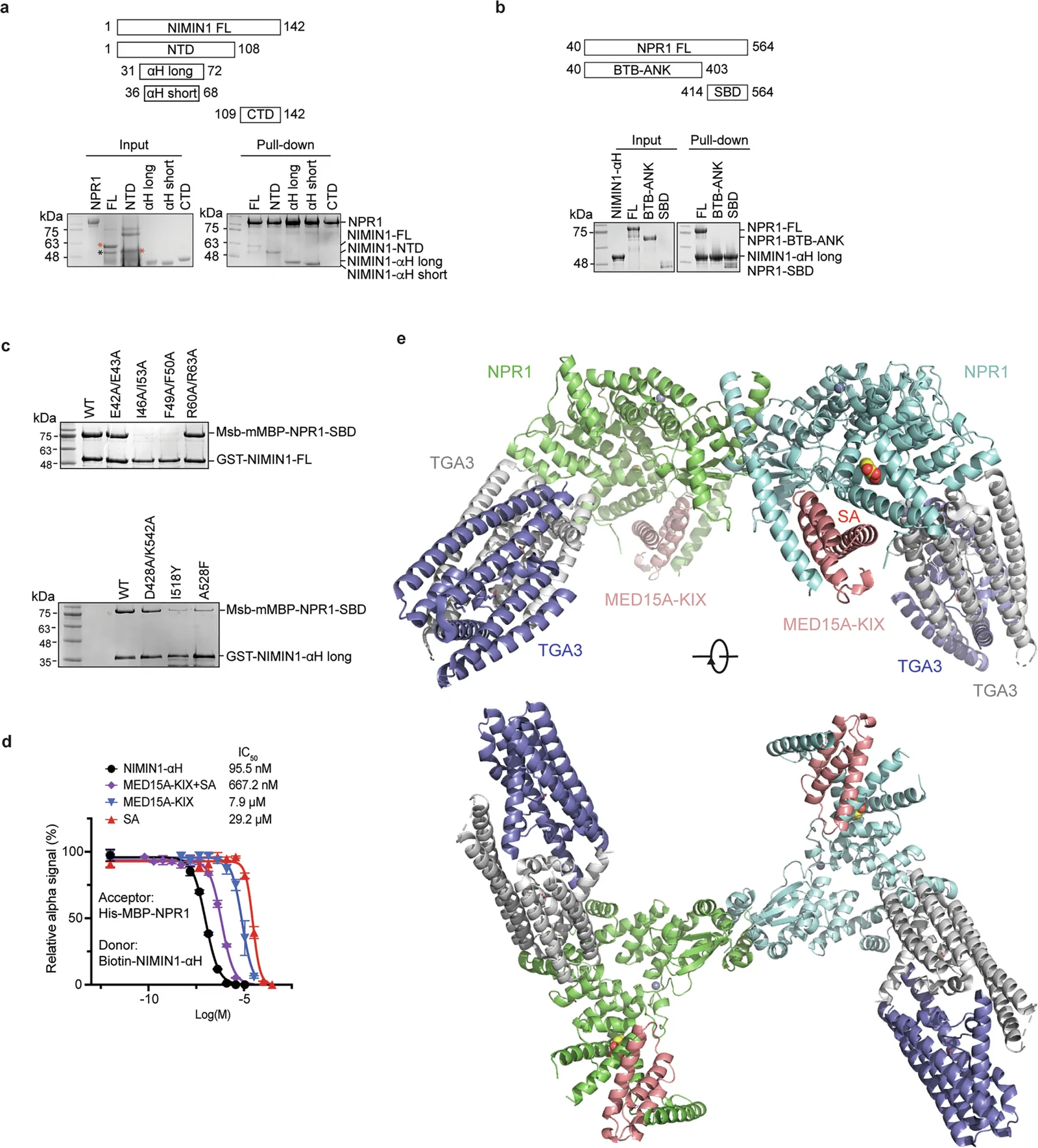
Analysis of NIMIN1-NPR1 interaction and a structural model of the NPR1-SA-MED15A^KIX^-TGA3 complex. **a**. Mapping of NPR1-interacting regions in NIMIN1 by affinity pull-down assay. FL: full-length; NTD: N-terminal domain; CTD: C-terminal domain. Red asterisk: intact NIMIN1 FL and NTD; black asterisk: a proteolytic product of NIMIN1 FL. **b**. Mapping of NIMIN1-interacting regions in NPR1 by affinity pull-down assay. **c**. Glutathione affinity pull-down assays assessing the impact of mutations of hydrophobic and charged residues at the NPR1-SBD and NIMIN1-αH interaction interfaces. **d**. Potency of tag-free NIMIN1-αH (short), SA, MED15A-KIX, and SA plus MED15A-KIX in competing with biotinylated NIMIN1-αH for NPR1 binding. For the competition measurement of SA plus MED15A-KIX, SA concentration was maintained at 3.7 μM while MED15A-KIX concentration was titrated up to 33 μM. Data are presented as mean values +/− SD (n = 3 reactions) **e**. A complete structural model of an SA-bound NPR1 dimer in complex with two TGA3 homodimers and two MED15A-KIX monomers. SA is shown in red-yellow spheres. Experiments in **a**, **b, c** and **d** were repeated three times. Source data

NIMIN1 is an NPR1-binding protein that acts as a transcriptional repressor of *PR* genes^21,37^. Elevated SA levels trigger the upregulation of NIMIN1 expression, which establishes a negative feedback loop to restrain the SA signalling pathway. Previous studies have mapped the NIMIN1-binding site on NPR1 to its C-terminal region and identified a minimal 38-amino acid binding fragment in NIMIN1 (residues 35–72)^37^. The NIMIN1–NPR1 interaction is incompatible with SA binding, indicating functional antagonism between NIMIN1 and the hormone^38^. Using recombinant proteins in an in vitro pulldown assay, we first confirmed the direct interaction between NPR1 and NIMIN1 and further narrowed down the NPR1-binding segment to residues 36–68 (Extended Data Fig. 4a). We then validated that NPR1-SBD is necessary and sufficient for recruiting the transcriptional repressor (Extended Data Fig. 4b).

**Fig. 5:**
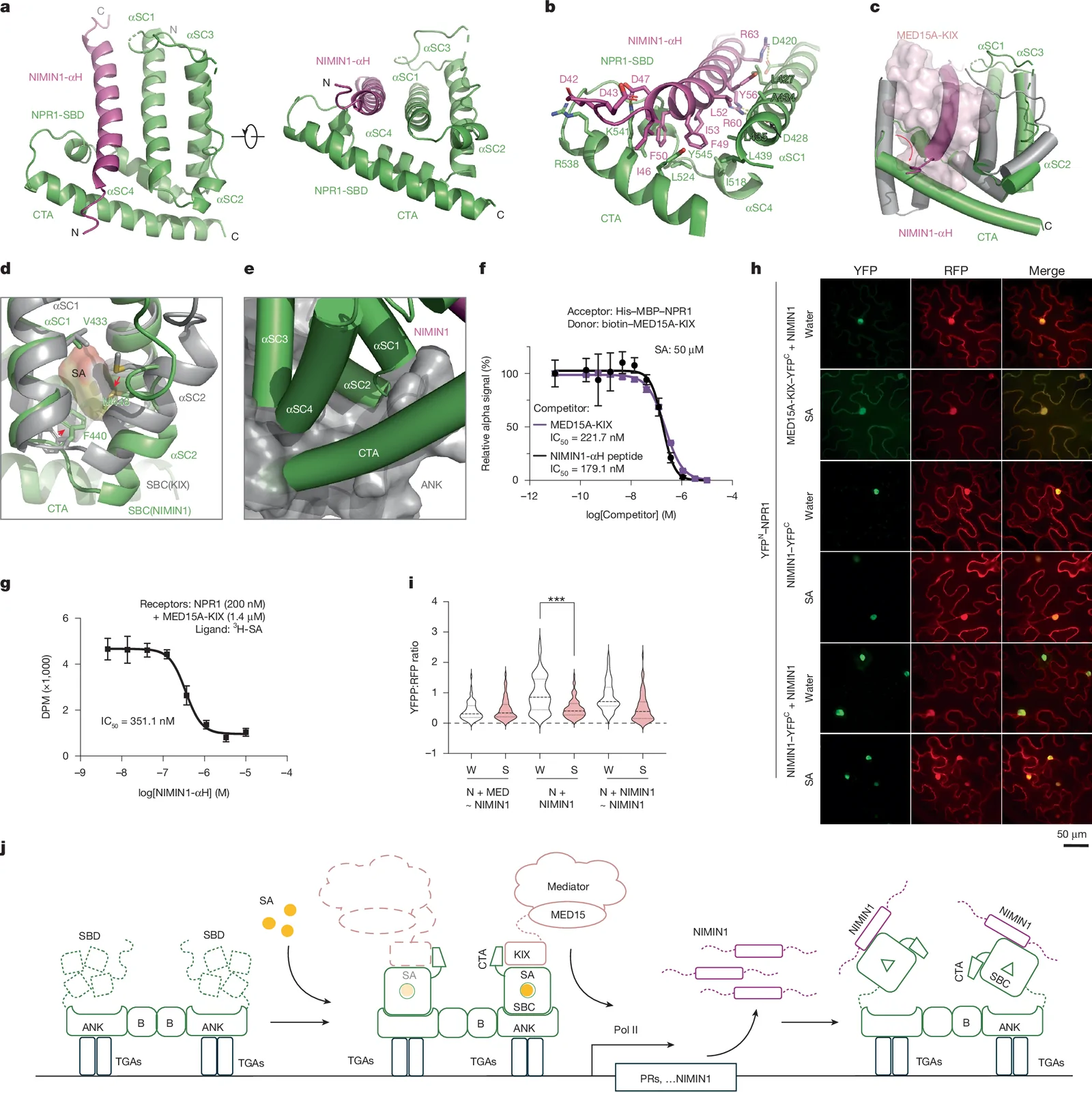
NIMIN1 blocks MED15A binding to NPR1. **a**, Orthogonal views of the NPR1-SBD–NIMIN1-αH complex. **b**, Close-up view of the NPR1-SBD–NIMIN1-αH interface with the side chains of interacting residues shown as sticks. **c**, A superposition of NPR1-SBD bound to NIMIN1 (magenta; NPR1-SBD in green) and MED15A-KIX (light pink in surface representation; NPR1-SBD in grey). The red arrow shows the rotation angle relating the NPR1 CTA helix in the two structures. **d**, Close-up view of the SA-binding site in the NPR1-SBC four-helix-bundle remodelled by NIMIN1. NIMIN1-bound and SA–MED15A-KIX-bound NPR1-SBC are coloured in green and grey, respectively. SA is shown as red–yellow surface. F440 and M448 are shown as sticks. The red arrows indicate their positional shift with V433 shown as reference. **e**, Steric hindrance between the NIMIN1-bound NPR1-SBC four-helical bundle and the NPR1 ANK domain when modelled onto the NPR1–SA–MED15A-KIX structure. **f**, AlphaLISA competition assay measuring the potency of the NIMIN1-αH short (residues 36–68) peptide in disrupting the SA-enhanced NPR1–MED15A-KIX interaction. **g**, Radio-labelled ligand-binding assay measuring the potency of the NIMIN1-αH short peptide in blocking SA binding to NPR1 with MED15A-KIX in excess. Data in panels **f**,**g** are presented as mean ± s.d. (*n* = 3 reactions), and experiments were repeated three times. **h**, In vivo interaction between YFP^N^-fused NPR1 and YFP^C^-fused NIMIN1 and the reduced interaction between YFP^N^-fused NPR1 and YFP^C^-fused MED15A-KIX in the presence of NIMIN1. **i**, Statistical analysis of the normalized YFP:RFP ratio. The dashed lines are the lower quartile, median and upper quartile, respectively, from bottom to top. The *P* value was calculated using two-tailed Welch’s *t*-test (*n* > 40 nuclei): ***P = 0.00001. Assays were performed in the same set of experiments as Fig. 2. Dashed line shows where the YFP:RFP ratio is 0. Tildes (~) indicate coexpression of NIMIN1 with YFP^N^- and YFP^C^-fused protein pairs. **j**, Schematic of the regulatory mechanisms of NPR1 by SA and NIMIN1. The dotted lines denote intrinsically disordered regions. B, BTB and BACK domains of NPR1; Pol II, RNA polymerase II. Source data

By leveraging maltose-binding protein (MBP) as a crystallization scaffold, we determined the crystal structure of MBP-fused NPR1-SBD in complex with the NIMIN1 fragment (residues 31–72) at 3.3 Å resolution (Fig. 5a and Extended Data Table 2). The structure revealed a NIMIN1-binding pose that is strikingly analogous to MED15A-KIX, despite substantial structural differences in the two. In contrast to the three-helix bundle of MED15A-KIX, the approximately 30 amino acids of the NIMIN1 fragment adopts a single long helical structure (hereafter referred to as αH), which lies in parallel with the αSC1 helix of NPR1-SBC (Fig. 5a,b). Their packing is further secured by the NPR1 CTA helix and its preceding loop, which folds into a short α-helix (Fig. 5b). Consistent with previous mutational analysis^20^, the critical NIMIN1–NPR1 interaction is mediated by two adjacent NIMIN1 phenylalanine residues, Phe49 and Phe50, which orchestrate a hydrophobic core together with a panel of hydrophobic residues in both their flanking regions and the NPR1-SBD helices (Fig. 5b). Mutations of these hydrophobic residues, but not surrounding charged amino acids, readily abolished NPR1–NIMIN1 interactions in affinity pulldown assays (Extended Data Fig. 4c). In comparison to the MED15A-KIX-bound NPR1-SBD, the NPR1 CTA helix undergoes an approximately 110° rotation to envelop NIMIN1-αH (Fig. 5c). As a result, the C-terminal half of the CTA helix is positioned to make close contact with the αSC1–αSC2 and αSC3–αSC4 loops in SBC. Superposition analysis indicates that, upon binding to NPR1-SBD, NIMIN1 would sterically occlude MED15A-KIX (Fig. 5c).

## NIMIN1 obliterates the SA-binding site

A close examination of NPR1-SBC revealed additional structural impact made by NIMIN1 binding that interferes with the cooperative binding of SA and MED15A to NPR1. First, the repositioned NPR1 CTA helix induces a major conformational change at the αSC1–αSC2 loop, which shifts the position of the αSC2 helix and remodels its C-terminal half into a coiled conformation (Fig. 5d). These structural changes reshape the SA-binding pocket, rendering it incompetent for ligand binding. In particular, the bulky and hydrophobic side chains of two residues, Phe440 and Met448, insert into the SA-binding pocket, partially blocking the ligand-binding site. Second, when superimposed with the MED15-KIX-bound NPR1 structure, the tapered end of the SBC four-helix bundle and the C-terminal tip of the CTA helix collide with the NPR1 ANK domain, hampering the SBC–ANK interaction (Fig. 5e). Overall, the NIMIN1 α-helix not only physically competes with MED15A-KIX for NPR1 binding but also allosterically abolishes the ligand-binding capacity of the SA receptor. Unlike MED15A-KIX, NIMIN1 can form a stable complex with isolated NPR1-SBD in the absence of the NPR1 ANK domain (Extended Data Figs. 3e and 4b), underlining its ability to sequester the SA-binding module away from the rest of the SA receptor.

Consistent with these structural results, the NIMIN1 αH peptide potently inhibited the NPR1–MED15A interaction with a half-maximal inhibitory concentration (IC_50_) of approximately 180 nM in an AlphaLISA-based competition assay (Fig. 5f). It also effectively abrogated the binding of SA to NPR1, even in the presence of MED15A-KIX, as determined by the radio-labelled ligand-binding assay (Fig. 5g). Accordingly, NIMIN1 strongly interacted with NPR1 as assessed by the BiFC assay. NIMIN1 co-expression diminished both the basal and SA-stimulated NPR1–MED15A-KIX interactions (Fig. 5h,i compared with Fig. 2b,c). The NIMIN1–NPR1 interaction, nevertheless, is reversible. At sufficient high concentrations, MED15A-KIX and SA synergistically displaced NIMIN1 from NPR1 (Extended Data Fig. 4d). SA treatment also weakened the NIMIN1–NPR1 signal, probably with assistance from endogenous MED15A (Fig. 5h,i). Together, these findings illuminate how NIMIN1 promotes disassembly of the NPR1–MED15A transcription-activating complex and underscore the structural plasticity of the NPR1 SBD domain in enabling dynamic positive and negative regulation.

## Discussion

Unlike classical plant hormones, such as auxins, jasmonates and gibberellins, which act on ubiquitin ligases to trigger the degradation of transcriptional repressors^39–41^, our findings suggest that SA promotes recruitment of the Mediator complex to NPR1, thereby bridging the transcriptional coactivator complex to the TGA transcription factors (Fig. 5j). Our structural studies allowed us to build a complete model of the fully assembled TGA–NPR1–SA–MED15A-KIX complex (Extended Data Fig. 4e), illustrating how an SA-bound NPR1 dimer can provide two MED15A-docking sites while engaging two TGA dimers. Tandem or multiple *as-1* elements in promoters, therefore, could enhance SA-responsive transcription by increasing the likelihood of Mediator recruitment and subsequent transcription initiation by RNA polymerase II (Fig. 5j).

SA and jasmonate are widely viewed as mutually antagonistic defence hormones in plant immunity^42^. Yet, at the mechanistic level, their signalling circuits mirror one another. SA-driven recruitment of MED15A by NPR1 parallels jasmonate-dependent docking of MED25, another Tail module subunit, to MYCs^43,44^. Whereas jasmonate promotes the turnover of the JAZ repressors that occlude MED25–MYC binding, SA enables NPR1 to tether MED15A to TGAs, a process that NIMIN1 can impede. These distinct yet parallel strategies highlight how plants harness the same Mediator co-activator hub by leveraging different subunits and binding logic to drive hormone-specific transcriptional outputs.

Together with earlier work, our findings have revealed a striking convergence among plant hormones in their ability to promote protein–protein interactions. Auxins and jasmonates act as molecular glues at protein interfaces, whereas SA, gibberellins, abscisic acids and strigolactones modulate protein interactions allosterically^18,45–50^. In the cases of SA and gibberellins, the hormones function as protein co-folders, fixing otherwise disordered structural elements in their receptors into a conformation competent for effector binding. In these systems, hormone-induced protein–protein interactions in return confer an apparent ligand potency that exceeds the affinity of the hormone for the free receptor, underscoring the cooperative nature of these tri-molecular assemblies. Deciphering how these naturally occurring small molecules rewire protein networks not only informs agronomic applications but also inspires protein design and future therapeutics development.

## Methods

### Molecular cloning

Coding sequences for the near full-length NPR1 (amino acids 40–564; AT1G64280), NPR3 (amino acids 33–561; AT5G45110), NPR4 (amino acids 30–552; AT4G19660), MED15A-FL (amino acids 1–1,335; AT1G15780), MED15A-KIX (amino acids 17–98; AT1G15780), MED15A-KIX long (amino acids 1–98), MED15E-KIX (amino acids 1–75; AT2G10440), MED15D-FL (amino acids 1–189; AT1G15790), MED15D-KIX (amino acids 1–93), NIMIN1-FL (amino acids 1–142; AT1G02450), NIMIN1-NTD (amino acids 1–108), NIMIN1-αH long (amino acids 31–72), NIMIN1-αH short (amino acids 36–68), NIMIN1-CTD (amino acids 109–142) and TGA3 (amino acids 87–384; AT1G22070) were amplified from the *A. thaliana* cDNA library with primers pairs that contain linker sequences at the 5′ ends for T4 DNA polymerase (NEB) treatment (forward: 5′-AAAACCTCTACTTCCAATCG-3′, reverse: 5′-CCACACTCATCCTCCGGTTA-3′) for ligation-independent cloning. To generate the Avi-tagged MED15A-KIX, MED15A-KIX (amino acids 17–98) was synthesized with an Avi tag followed by a GSGSGSGS linker at the 5′ by Integrated DNA Technologies and fused after the TEV cleavage site. The loop-deletion NPR1-SBD (amino acids 415–564 with amino acids 460–483 replaced with a GSGSG linker) and the mammalianized MBP (mMBP)^51^ were synthesized by Azenta/Genewiz. The mMBP–NPR1-SBD fusion construct, where mMBP was used as a crystallization chaperone, was generated by fusing the loop-deletion NPR1-SBD to the last α-helix of mMBP through overlap PCR with a linker sequence encoding five alanine residues (A5) between the two^52^. The NPR1 and MED15A-KIX mutant inserts were made either through overlap PCR or using the Q5 Site-Directed Mutagenesis Kit (New England Biolabs). For His–MBP-tagged constructs, the inserts were cloned into the pAL vector for *Escherichia coli* expression system or into HTB vector for insect cell. For His–Venus, the pACE vector that was modified for expressing in both *E. coli* and the insect cell systems was used. For His–Msb (*E. coli* acidic protein msyB)^53^, a pET vector was used. T4 DNA polymerase-treated inserts were incubated with T4 DNA polymerase-treated vectors at a 2:1 molar ratio at room temperature for 5 min before transformation into DH5α-competent or Stbl3-competent cells. For all constructs, the inserts were fused with a 6×His tag followed by a MBP, Venus or Msb protein and a TEV cleavage site at the N terminus. Plasmids were miniprepped with Qiagen miniprep kit following the manufacturer’s instructions and confirmed via Sanger sequencing or whole-plasmid sequencing at Genewiz (Azenta). Positive plasmids were either transformed into BL21-competent cells for expression in *E. coli* or into DH10Bac cells for bacmids. For insect cell expression, DH10Bac cells were cultured in LB overnight at 37 °C before being resuspended, lysed and then neutralized with 250 µl of P1, 250 µl of P2 and 350 µl of N3 buffers from the Qiagen miniprep kit, respectively. The mixture was incubated on ice for 10 min before being centrifuged at top speed for 10 min. The supernatant was mixed with equal volume of 2-propanol and incubated on ice for 30 min before centrifuging for 15 min at room temperature. The resulting pellet was washed with 500 µl of 70% ice-cold ethanol twice, air dried and eventually dissolved in 40 µl of ddH_2_O. Recombinant virus was made using Sf9 monolayer cells (Thermo Fisher Scientific, Gibco), and proteins were expressed in Hi5 monolayer cells (Thermo Fisher Scientific, HighFive) in the baculovirus expression vector system (Invitrogen).

### Protein expression and purification

*E. coli* cells expressing the target proteins were cultured at 37 °C to optical density at 600 nm (OD_600_) of 0.8–1 and induced with 0.2–0.5 mM IPTG at 16 °C overnight. Cells were harvested by centrifugation at 3,000*g*, resuspended and lysed in lysis buffering (200 mM NaCl, 20 mM Tris HCl pH 8, 0.5 mM TCEP and 20 mM imidazole pH 8) and 1 mM PMSF by sonicating with 30% amplitude, 0.5 s ON–0.5 s OFF for 5 min. The sonicated crude was then centrifuged at 39,375*g* for 50 min at 4 °C. The supernatant or lysate was loaded on a column that contained 2 ml of nickel or amylose resin and was pre-equilibrated with lysis buffer. After being washed with lysis buffer, protein on the resin was eluted with lysis buffer containing either 200 mM imidazole (for nickel resin) or 10 mM maltose (for amylose resin). The eluted protein was mixed with 4× Laemmli buffer (277.8 mM Tris-HCl, pH 6.8, 44.4% (v/v) glycerol, 4.4% SDS and 0.02% bromophenol blue) and confirmed on an SDS–PAGE gel and by TEV cleavage. Purification procedures for proteins expressed in insect cell were the same as above with a few modifications. Insect cells were pelleted by centrifugation at 2,000*g* for 15 min, resuspended and lysed in the same lysis buffer supplemented with 1 mM PMSF and protease inhibitors including leupeptin, aprotinin and pepstatin. The elution was then concentrated, centrifuged at top speed at 4 °C for 10 min before size exclusion using Superdex 200 increase 10/30 gl (Cytiva) in sizing buffer (150 mM NaCl, 20 mM HEPES pH 7.5 and 0.5 mM TCEP). Unless otherwise stated, NPR1 (amino acids 40–564) and MED15A-KIX (amino acids 17–98) were used in this study.

### In vitro pulldown assays

To detect the interaction between MED15s and NPRs, His–MBP–MED15s and His–Venus–NPRs were expressed in *E. coli* separately as described above. As His–Venus-tagged NPRs were associated with multiple proteolytic products, to control the amount of the input proteins for the pulldown experiments, 4 µg of each protein was run on an SDS–PAGE gel to calculate the percentage of the intact protein in the sample. The bands of the intact proteins were quantified with the Image Lab (v5.1) software (Bio-Rad). The concentration of the intact proteins was calculated accordingly. A total of 20 µg of His–MBP–MED15 was mixed with His–Venus–NPR at a 1:2 molar ratio with or without 200 µM SA and incubated on ice for 20 min. The mixture was then applied to 20 µl amylose resin (NEB) that was pre-equilibrated with sizing buffer, gently mixed and incubated on ice for 1 h. The supernatant was removed after centrifugation at 600*g* for 1 min. The resin was washed three times with 200 µl of sizing buffer. To elute, the resin was resuspended with 40 µl of 1× sample buffer and boiled at 100 °C for 10 min. After centrifugation at top speed for 1 min, the supernatant was collected and loaded on an SDS–PAGE gel that was run in 1× Rapid Running Buffer Solution (Nacalai Tesque) at 210 V for 25 min. For gel source data, see Supplementary Fig. 1.

For the pulldown experiment involving full-length NPR1 and various NIMIN1 constructs, His–MBP–NPR1 was used to pull down His–Venus–NIMIN1s. In the experiment assessing the interaction between NIMIN1 αH and various NPR1 constructs, the His–MBP-tagged NIMIN1-αH long (amino acids 31–72) was co-expressed with His–Msb-tagged NPR1-FL, NPR1-BTB-ANK or NPR1-SBD and purified with amylose resin. In the experiment in which the interaction between NPR1-SBD and MED15A-KIX or NIMIN1-αH long (amino acids 31–72) was examined, His–mMBP-tagged NPR1-SBD was used to pull down His–Venus-tagged MED15A-KIX or His–Venus-tagged NIMIN1-αH long. To pull down the MED15A-FL, His–MBP–NPR1 and His–Venus–MED15A were expressed together in the insect cell. The co-expression samples were assessed side by side with the two separately expressed proteins. For the SA-treated samples, 200 µM SA was included in buffers throughout the pulldown assays. For in-gel Venus florescence detection, protein samples were incubated in 1× sample buffer at room temperature for 5 min, and unboiled samples were loaded to the SDS–PAGE gel; before Coomassie blue staining, Venus fluorescence was detected with DyLight 488 Blot, and protein ladder was imaged with DyLight 680 Blot using the Image Lab Touch software (v3.0.1.14) built in the ChemiDoc MP imaging system (Bio-Rad).

### Biolayer interferometry

The binding between NPR1 and MED15A-KIX with or without SA was detected using Octet Red 96 (ForteBio, Pall Life Sciences). The Octet streptavidin biosensors (Sartorius, for kinetics) were equilibrated in buffer for 60 s, pre-activated with 100 nM biotinylated Venus nanobody for 20 s and quenched with 200 nM biocytin for 60 s. His–Venus-tagged MED15A-KIX (200 nM) was then immobilized on the Venus nanobody-coated biosensors for 22 s. After being rinsed in buffer for 60 s, the MED15A-KIX-loaded biosensors were then dipped into a serial dilution of His–MBP–NPR1 purified from insect cell or His–mMBP–NPR1-SBD with or without SA for association and back to buffer for dissociation. All reactions were carried out at 30 °C in a Greiner black 96-well microplate containing 200 μl of sizing buffer containing 0.02% Tween-20 and 0.1% ovalbumin. The buffer-only well served as the instrument internal control, and the MED15A-KIX-absent well was the reference. Data collection was done using the Octet BLI Discovery software (v13.0.3.26). The dissociation constant between MED15A-KIX and NPR1 was determined from the steady-state equilibrium responses using the Octet BLI Analysis (v12.2).

### AlphaLISA binding assays

ALPHA was carried out in a Revvity 96-well white 1/2 area microplate sealed with a clear adhesive microplate seal (Revvity TopSeal-A PLUS). Plates were read using a PerkinElmer EnSpire 2300 Multilabel Reader. All the incubation was done with constant shaking at room temperature. His–MBP–NPR1 purified from insect cell was immobilized on the AlphaLISA 6His Nickel Chelate Acceptor Beads (Revvity), whereas the biotinylated MED15A-KIX or biotinylated NIMIN1-αH peptide (amino acids 36–68, biotin-EDEEEEEEKKIDTFFKLIKHYQEARKRRREELA) was captured on the Alpha Streptavidin Donor Beads (Revvity). Avi-tagged MED15A-KIX was biotinylated as previously described^54^. A cross-titration was performed for each pair (NPR1–MED15A-KIX or NPR1–NIMIN1-αH) before competition assays. Specifically, each protein of a pair was cross-titrated with serial dilutions from the high pM range to 1 µM. The optimal low concentration for each protein was determined to avoid the hooking effect in the following titration assays. The NPR1–MED15A-KIX cross-titration was carried out in the presence of 50 µM SA. For all cross-titrations, the two proteins were mixed first with shaking at room temperature for 1 h followed by a 1-h incubation with 10–20 µg µl^−1^ acceptor beads, and finally with a 1-h incubation with 10–20 µg µl^−1^ donor beads in the dark in a 40–50-µl reaction system. The reaction buffer contained sizing buffer, 0.02% Tween-20 and 0.1% bovine serum albumin. Plates were read 1 h after all components were added. For competition–signal reduction assays, competitors (non-tagged MED15A-KIX or non-biotinylated NIMIN1-αH peptide) were mixed with NPR1 for 1 h followed before incubations with other components. For the assays with the NPR1–MED15A-KIX pair, 111.1 nM His–MBP–NPR1 and 12.3 nM biotinylated MED15A-KIX were used in each 50-μl reaction, and for the other pair, 5 nM His–MBP–NPR1 and 30 nM biotinylated NIMIN1-αH peptide were used in each 40-μl reactions. For SA–benzoic acid titration assays, up to 270 µM SA or benzoic acid was titrated into 111.1 nM His–MBP–NPR1 and 12.3 nM biotinylated MED15A-KIX. The experiments were performed in three replicates. IC_50_ was determined using non-linear curve fitting of the dose–response curves generated with Prism (GraphPad).

### ITC

The effect of SA on the NPR1–MED15A-KIX interaction was examined using ITC. Measurements were conducted at 25 °C on a Malvern MICROCAL PEAQ-ITC instrument with the Malvern MICROCAL PEAQ-ITC Control software (v1.41). SA was loaded into the syringe and delivered into the sample cell via an initial 0.4-µl injection (0.8 s), followed by 18 2-µl injections (4 s each) with 150-s intervals and stirring at 750 rpm. To determine the NPR1–SA-binding affinity, 125 µM SA was titrated into 250 µl of 10 µM His–MBP–NPR1 (amino acids 40–564) purified from *E. coli*. To measure the cooperative binding of SA and MED15A-KIX, 100 µM SA was titrated into 250 µl of a mixture containing 27 µM His–MBP–NPR1 and 53.6 µM His–MBP–MED15A-KIX. A baseline heat response was obtained by titrating 100 µM SA into 250 µl of sizing buffer alone. Data were processed using the Malvern MICROCAL PEAQ-ITC analysis software (v1.41). Experimental thermograms were referenced against the buffer control to derive accurate dissociation constants.

### Tritium-labelled SA membrane filter assay

Ring-^3^H SA (American Radiolabeled Chemicals, 20 µM in 20% ethanol, 1 mCi ml^−1^, 50 Ci mmol^−1^) was used to detect SA-specific binding to NPR1 in the absence and presence of MED15A-KIX in a vacuum filtration system. Specifically, 50 µl of 400 nM His–MBP–NPR1 purified from insect cell, 4 µM His–Venus–MED15A-KIX or the mixture of both proteins was incubated with equal volume of a mixture of cold (unlabelled) and hot (^3^H labelled) SA (8.6:1 cold:hot) at various concentrations in sizing buffer. Each reaction had three replicates. The samples were incubated in glass vials at room temperature for 30 min. Immediately before filtration, the 100-µl reactions in vials were diluted with 2.5 ml cold sizing buffer and quickly passed through 0.45-µm MCE membranes (MF-Millipore, 25 mm, hydrophilic) that were pre-soaked in sizing buffer overnight. The vials were further rinsed with 2.5 ml of cold sizing buffer twice. The membranes were subsequently transferred into clean scintillation vials and submerged in 5 ml of scintillation liquid (ULTIMA Gold, PerkinElmer). ^3^H radioactivity was read 2 min per sample with a scintillation counter (Beckman LS6500). For the NIMIN1 competition assay, each 100-µl reaction had 200 nM His–MBP–NPR1, 1.4 µM His–Venus–MED15A-KIX and 1 µM SA (8.6:1 cold:hot) and NIMIN1-αH peptide at various concentrations using the Tri-Carb 4910 TR Liquid Scintillation Analyzer (Revvity) with the same settings in the QuantaSmartTM for Tri-Carb (v5.3; Revvity).

### Ratiometric BiFC

The 2in1 Plasmid Toolkit was a gift from C. Grefen (AddGene kit #1000000147). The constructs were made as previously described^55^. Specifically, coding sequences for NPR1, MED15A-KIX, MED15E-KIX, MED15D-FL and the full-length NIMIN1 were amplified from the constructs that were used for in vitro pulldown assays. The coding sequence for the *Arabidopsis* SKP1 (ASK1, AT1G75950), a negative control, was amplified from the *A. thaliana* cDNA library. NPR1 (with a stop codon) was designed to fuse to the C terminus of the N-terminal half of the YFP, and the others (without a stop codon) were fused to the N terminus of the C-terminal half. Primers consisted of the insert-specific sequence and the overlapping sequence with the entry vector pUC-L1L4 (for MED15s, NIMIN1 and ASK1 forward primers: 5′-ggctctagagtcgac-3′, reverse primers: 5′-gttgggtggccggcctgcag-3′) or pUC-L2L3 (for NPR1 forward primer: 5′-aagttgctagcgtcgac-3′, reverse primer: 5′-tgggtagccggcctgcag-3′). The entry vectors and inserts (except for ASK1) were linearized by double restriction digestion using SalI-HF and PstI (NEB) and gel extracted. To make the pUC-L1L4-ASK1 construct, pUC-L1L4 and ASK1 was linearized with restriction enzymes XbaI and PstI instead. The restriction-digested inserts and vectors were ligated using T4 DNA ligase (NEB) following the manufacturer’s instructions. The ligation mixture was used for transformation into *E. coli* (Stbl3)-competent cells. Colonies were picked, miniprepped (Qiagen) and confirmed with whole-plasmid sequencing (Genewiz, Azenta). The positive recombinant entry constructs were mixed the destination vector pBiFC-2in1-NC in a Gateway LR reaction to get the recombinant constructs. Expression of NPR1 fusions, MED15s–NIMIN1–ASK1 fusions and the red fluorescent protein (RFP) were all driven by independent 35S promoters. The resulting constructs were then transformed into *Agrobacterium* (GV3101) via electroporation. Colonies carrying the construct were validated with PCR for both NPR1 and the other inserts (MED15s, NIMIN1 and ASK1). *Agrobacterium* culture, activation and infiltration was carried out according to a previously published procedure^56^ with the following modifications. *Agrobaterium* at OD_600_ of 0.5 was activated in AS medium (150 µM acetosyringone, 10 mM MES-KOH pH 5.6 and 10 mM MgCl_2_) at room temperature for 3 h. Tobacco (*Nicotiana benthamiana*) plants were grown in growing mix soil (sungro) at 25 °C with a 16-h photoperiod with 100–150 μmol m^−2^ s^−1^ light intensity. The second and the third true leaves of six healthy 4-week-old tobacco plants were infiltrated with *Agrobacterium* carrying the appropriate constructs. After 48 h post-infiltration, the leaves of three individual plants were infiltrated again either with 10 mM MgCl_2_ (‘water’ or ‘mock’) or with 10 mM MgCl_2_ and 0.5 mM SA. Four leaf discs were collected for imaging with a hole puncher 5 h after the treatments. For NIMIN1 competition BiFC experiments, NIMIN1-FL was amplified with forward primer containing 5′-ggggacaagtttgtacaaaaaagcaggcttc-3′ and reverse primer containing 5′-ggggaccactttgtacaagaaagctgggtc-3′, besides the NIMIN1-specific sequences. NIMIN1 was cloned into the pDONR-Zeo entry vector and then the pEARLEYGate202 destination vector using Gateway Cloning. *Agrobacterium* carrying pBiFC-2in1-NPR1 + MED15A-KIX or pBiFC-2in1-NPR1 + NIMIN1 was mixed with the cells carrying pEARLEYGate202-NIMIN1 at a 1:1 ratio to OD_600_ of 0.5 for infiltration.

### Confocal laser scanning microscopy and data analysis

All imaging was performed on a Nikon Ti2 Eclipse-CSU-X1 confocal spinning disc microscope equipped with four laser lines, 405, 488, 561 and 670 nm, and the Nikon Elements software NIS-Elements AR5.20.01. To detect YFP and RFP, the 488-nm and 561-nm lasers were used at 15% laser power, 100 mW across all samples. An Andor Zyla4.2 Plus sCMOS camera was used for image acquisition with a Nikon ×40/1.30 oil objective and with 100-ms exposure and 0.5-µm *z*-step set in the Nikon Elements software. All image analyses were done using the open access Fiji ImageJ software (v2.16.0/1.54p). As YFP was exclusively detected in the nuclei of tobacco epidermal cells, to measure the florescence intensity, a 11.663-µm^2^ region of interest (ROI) was applied in all the nuclei assessed. For each treatment, the YFP and RFP signal, and the background signal in each channel, from over 40 nuclei was measured. The YFP:RFP ratio was calculated as (YFP_nucleusROI_ − YFP_backgroundROI_)/(RFP_nucleusROI_ − RFP_backgroundROI_). The effects of SA treatment on NPR1–MED15A-KIX, NPR1–MED15A-KIX(E90K) and NPR1–NIMIN1 were analysed using two-tailed Welch’s *t*-test in Prism 10 (v10.6.1, GraphPad).

### Semi-in vitro precipitation assay

*A. thaliana* (Col-0) plants were grown in growing mix soil (Sungro) at 25 °C with a 16-h photoperiod with 100–150 μmol m^−2^ s^−1^ light intensity. To induce the endogenous NPR1 level, 3-week-old *Arabidopsis* plants were sprayed with 1 mM SA with 0.1% (v/v) Silwet L-77 (Fisher Scientific) as the surfactant 24 h before use. Approximately 8 g of SA-treated leaves were harvested and ground in liquid nitrogen with a pestle and a mortar into 30 ml of extraction buffer (150 mM KCl, 50 mM HEPES pH 7.5, 10 mM EDTA, 0.5 mM Triton X-100, 20 mM dithiothreitol, 7 µM MG132, 1 mM PMSF and protease inhibitor cocktail). The crude extract was briefly sonicated with 20% amplitude, 0.5 s ON–0.5 s OFF for 2 min in iced water followed with centrifugation at 39,375*g* for 30 min at 4 °C. The lysate was then filtered into a new tube with a 0.45-µm filter (Fisher Brand). The baits, His–MBP-tagged MED15A-KIX, MED15A-B-KIX and MED15A-C-KIX, were purified from *E. coli* as described above. Proteins were adjusted to 1 mg ml^−1^ before use. EDTA-compatible nickel resin (Pierce High-Capacity EDTA-Compatible Ni-IMAC Resin) was equilibrated with sizing buffer and then incubated with saturating amount of each of three proteins on a rotator for 1 h at 4 °C. The supernatant was removed by centrifugation at 600*g* for 1 min. His–MBP–MED15-bound resin was then washed 3 times with 10 bed volumes of sizing buffer. To pull down the endogenous NPR1, 50 µl of resin was mixed with 1.5 ml of lysate containing 10 mM imidazole with or without 200 µl SA and incubated at 4 °C on a rotator overnight. The next day, the resin was invert incubated and washed with 1.5 ml of sizing buffer three times, each for 5 min before being boil-eluted (100 °C for 10 min) into 100 µl of 1× Laemmli buffer with 50 mM dithiothreitol. To assess the inputs, 3 µl of the elution was loaded on an SDS–PAGE gel, stained with Coomassie blue (40% ethanol, 0.1% Brilliant Blue R250 and 25% acetic acid) and destained for imaging. To detect endogenous NPR1, 20 µl of the elution was loaded on 4–15% Mini-Protean TGX gel (Bio-Rad). The gel was run with 1× Tris-glycine SDS running buffer at 150 V for 45 min and subsequently transferred to 0.2-µm PVDF membrane (Bio-Rad Trans-Blot Turbo, mini) with Trans-Blot Turbo (Bio-Rad) using the mixed molecular weight protocol (1.3 A at 250 V for 7.5 min). The membrane was washed with 1× TBS-T for 10 min twice before being blocked in the blocking solution (1× TBS-T and 5% milk) for 1 h at room temperature. The blocked membrane was washed with 1% milk in 1× TBS-T for 10 min twice before being incubated with the primary NPR1 antibody (gift from Z. Mou; rabbit, 1:5,000 dilution) on a shaker at 4 °C overnight. The next day, the membrane was washed with 1% milk in 1× TBS-T three times for 15 min and incubated with the secondary antibody (1:10,000 dilution; ECL horseradish peroxidase-conjugated anti-rabbit IgG, whole antibody from Cytiva) in 1× TBS-T and 1% milk at room temperature for 1 h. The membrane was washed again as above, and washed once with 1× TBS-T for 10 min. Freshly prepared SuperSignal West Femto Maximum Sensitivity Substrate (Protein Biology) was applied to the membrane. The signal was imaged with Chemiluminescent Blot, whereas the protein ladder was imaged with DyLight 680 Blot using the ChemiDoc MP imaging system (Bio-Rad).

### Electrophoretic mobility shift assay

The electrophoresis mobility shift assay assay was carried out following a previously reported protocol^13^ with the following modifications. One strand of the LS5 and LS7 *as-1* *cis*-elements of the SA-responsive *PR1* gene promoter *as-1* (LS5/LS7) was labelled with IRD-700 at the 5′ end (IRD700-5′- gggCTATGACGTAAGTAAAATAGTGACGTAGAGAggg-3′), whereas the other stranded remained unlabelled (5′-cccTCTCTACGTCACTATTTTACTTACGTCATAGccc-3′). The two oligos were dissolved in 1× TE buffer (10 mM Tris-HCl pH 8 and 1 mM EDTA) to make 100 µM stocks, which were mixed at a 1.25:1 ratio (labelled:unlabelled) with a slight excess of labelled strand as an endogenous loading control, and diluted with STE buffer (100 mM NaCl, 10 mM Tris-HCl pH 8.0 and 1 mM EDTA) to get 2 µM double-stranded DNA stock by annealing in boiled water that gradually cooled down to room temperature. The annealed stock was stored at −20 °C in dark until use. The 4–12% TBE gel (Invitrogen) was pre-run in 0.5× TBE buffer (44.5 mM Tris-HCl, 44.5 mM borate and 1 mM EDTA pH 8.3) at 100 V for 30 min. To determine the minimal amount of DNA to use in the assays, the annealed DNA was loaded on the gel at various concentrations (from 847 pM to 50 nM). A follow-up electrophoresis mobility shift assay, where TGA3 (amino acids 87–384) was titrated into 600 pM DNA, was carried out to determine the optimal TGA3 concentration. Two more assays were carried out to determine the optimal concentrations of NPR1 and MED15, respectively. Each component was diluted in the reaction buffer (150 mM NaCl, 20 mM HEPES pH 7.5, 0.5 mM TCEP, 1 mM dithiothreitol and 0.5% Tween-20) to make the working stocks. In the final assay, each 9-µl reaction contained 100 ng µl^−1^ poly(dI-dC) (Thermo Fisher) and 600 pM DNA. His–MBP–TGA3 (amino acids 87–384; 50 nM), His–MBP–NPR1 or His–MBP–NPR4 (15 µM), His–MBP–MED15A-KIX (23.4 µM) and SA (200 µM) were added sequentially across reactions. The reactions were incubated on ice for 30 min, and before loading, 1 µl of 10× orange loading dye (65% sucrose, 10 mM Tris-HCl pH 7.5, 10 mM EDTA and 0.3% (w/v) orange G) was added. The gel was run at 100 V for 100 min at 4 °C and detected using Image Studio (v5.2) (LICORbio) and the LI-COR Odyssey CLx scanner.

### Cryo-EM sample preparation and data collection

His–MBP–MED15A-KIX was co-expressed with His–Venus–NPR1 in *E. coli* (BL21) in LB supplemented with 200 µM SA. The complex was purified with amylose resin. The tags were removed by incubating the protein complex with TEV at 10:1 molar ratio overnight. The TEV-treated sample was then concentrated for size exclusion with Superdex 200 increase (Cytiva) in sizing buffer to separate out the tags and TEV from the complex. The purified NPR1–MED15A-KIX complex at 2 mg ml^−1^ was then crosslinked with 2 mM BS3 (Thermo Fisher Scientific) at room temperature for 30 min. The crosslinking reaction was quenched with 50 mM Tris-HCl pH 7.5. To get rid of the crosslinker, the crosslinked sample was purified again with size exclusion. SA at 200 µM was included in all purification steps. The crosslinked NPR1–MED15A-KIX complex was then concentrated to a concentration between 2 and 3.5 mg ml^−1^. Immediately before grid preparation, 9 µl of the complex was mixed with 1 µl of 10× DM (1%; detergent E from the VitroEase Buffer Screening Kit, Thermo Fisher Scientific). Glow-discharged amorphous Ni-titanium (ANT) holey foil grids (gold support, 300 mesh, 1.2/1.3 aperture Pitch; Molecular Dimernsions, Calibre Scientific) were used. Three microlitres of the sample was manually applied on the ANT grid for 20 s before manfully blotted at room temperature. Manual application and blotting were repeated one more time. The grid was then loaded into the 10 °C, 100% humidity Vitrobot (Vitrobot Mark IV System, Thermo Fisher Scientific), where another 3 µl of sample was applied and two-sided blotted with two layers of blot paper with blot force 0 for 9 s in the Vitrobot chamber. The grid was plunged into liquid ethane and stored in liquid nitrogen for clipping. The clipped grid was screened with Talos Glacios (Thermo Fisher Scientific) equipped with a K3 camera. Data collection was carried out on a Titan Krios transmission electron microscope (Thermo Fisher Scientific) operated at 300 kV at the University of Washington as previously described^54^. The automation scheme was implemented using the SerialEM software (v4.1)^57^ at a nominal magnification of 105,000, resulting in a physical pixel size of 0.829 Å; a total dose of 60 e^−^ Å^−^^2^ for each image fractionated into 100 frames. The images were recorded at a defocus range of 1–2 μm.

### Cryo-EM image processing and three-dimensional reconstruction

A total of 21,687 movies were acquired from two grids and processed in CryoSparc (v4.4.1)^58^. Beam-induced motion of each micrograph stack was corrected by patch motion correction. The defocus parameter of each motion-corrected micrograph was determined by contrast transfer function estimation. After filtered with contrast transfer function parameters and visual inspection, 15,862 micrographs were kept for subsequent processing. A total of 7,233,717 particles were blob picked, extracted and subjected to two rounds of two-dimensional classification. A subset of the selected particles in decent two-dimensional classes were used for ab initio reconstruction and heterogenous refinement. Particles from the good reconstruction were cleaned up again with three more rounds of heterogenous refinement with a good heterogenous refinement volume from a previous data collection included. A total of 773,558 particles were kept, re-extracted without Fourier crop to box size and subjected to non-uniform refinement^59^ to generate a reconstruction with an overall resolution of 3.22 Å.

### Cryo-EM structural model building and refinement

The initial structural model of dimeric NPR1–MED15A-KIX was predicted with AlphaFold3 at the AlphaFold Server^60^, and that of the dimeric NPR1–MED15A-KIX in complex with SA was predicted with Protenix^61^. The predicted models were fitted into the 3.22 Å cryo-EM density map using UCSF ChimeraX (v1.9)^62^. Subsequently, the model was inspected and manually refined in Coot (v0.9.8.95; ccp4) based on the protein sequences and the electron microscopy density. The model was further improved by real-space refinement in PHENIX (v1.21.2-5419) and manual rebuilding in Coot^63–65^. The final model was obtained from refinement using Rosetta^66,67^ and then real-space refinement in PHENIX again. PyMOL (v2.6.2; The PyMOL Molecular Graphics System, Schrödinger) was used to generate figures.

### Crystallization, data collection and structural determination

Crystals of the mMBP–A5–NPR1-SBDΔ4–NIMIN1-αH complex were grown at 4 °C by the hanging drop vapour diffusion method using 0.225 µl protein sample, supplemented by 10 mM maltose, mixed with 0.075 µl reservoir solution (0.2 M ammonium chloride, 0.1 M Na-HEPES pH 7.5 and 25% w/v PEG 3350). The largest crystal was harvested and flash-frozen in the crystallization condition with 27% sucrose at −170 °C. The X-ray diffraction dataset was collected at a wavelength of 1 Å at the BL2.0.1 beamline at the Advanced Light Source in Berkeley and was integrated and scaled by the XDS package^68^. The complex structure was solved by molecular replacement using PHENIX with a structural model predicted by AlphaFold3. The complex structure model was rebuilt, refined and ligand fitted using Coot^63^ and PHENIX^64,65^ (final Ramachandran statistics: 97.81% favoured, 1.71% allowed and 0.48% outliers). PyMOL (The PyMOL Molecular Graphics System, v2.0, Schrödinger) was used to generate figures.

### HDX-MS

Stock concentrations of NPR1 (3.4 µM) were pre-incubated either alone or in a complex with either a 300-fold excess of SA or a 300-fold excess of SA and 3-fold excess of MED15A-KIX for 4 h. Each stock was diluted into 90 µl of deuterated buffer (150 mM NaCl, 20 mM HEPES pH 7.5, 0.5 mM TCEP and 85% D final) containing 0.2 nM bradykinin and incubated for 3 s, 1 min, 20 min or 2 h at 21 °C. Each starting stock also included a mixture of imidazolium compounds to serve as exchange reference standards^69^. At the desired time point, the sample was rapidly mixed with an equal volume of ice-cold 200 mM TCEP, 0.2% formic acid and 0.1% trifluoroacetic acid for a final pH of 2.5. Samples were then immediately frozen on ethanol/dry ice and stored at −80 °C until liquid chromatography–mass spectrometry analysis. Undeuterated samples were prepared the same way but with undeuterated buffer for each step.

Samples were thawed at 5 °C for 8 min and injected using a custom LEAP robot integrated with a liquid chromatography–mass spectrometry system^70^. The protein was first passed over a Pepsin column (2.1 × 30 mm; AffiPro) at 400 µl min^−1^ for inline digestion with the protease column held at 20 °C. Peptides were then trapped on a Waters XSelect CSH C18 trap cartridge column (2.1 × 5 mm, 2.5 µm) and resolved over a CSH C18 column (1 × 50 mm, 1.7 µm, 130 Å) using linear gradient of 5–35% B (A: 0.1% formic acid, 0.025% trifluoroacetic acid and 5% acetonitrile; B: acetonitrile with 0.1% formic acid) over 10 min and analysed on a Thermo Orbitrap Ascend mass spectrometer at a resolution setting of 120,000. A series of washes over the trap and pepsin columns was used between injections to minimize carry-over as previously described^70^. Data-dependent tandem mass spectrometry acquisition was performed on an undeuterated sample using rapid collision-induced dissociation and higher-energy collisional dissociation scans and processed in Byonic (Protein Metrics) with a score cut-off of 150 to identify peptides. Deuterium incorporation was analysed using HDExaminer (v3; Trajan Scientific and Medical). Spectra displaying bimodal behaviour were exported from HDExaminer and analysed in HX-Express (v3)^71^.

### Reporting summary

Further information on research design is available in the Nature Portfolio Reporting Summary linked to this article.

## Online content

Any methods, additional references, Nature Portfolio reporting summaries, source data, extended data, supplementary information, acknowledgements, peer review information; details of author contributions and competing interests; and statements of data and code availability are available at https://doi.org/10.1038/s41586-026-11021-5.

## Supplementary information

Supplementary Figure 1 Uncropped gel and blot images

Reporting Summary

Supplementary Table 1 HDX-MS data and statistics summary

Peer Review File

## Source data

Source Data Fig. 1

Source Data Fig. 2

Source Data Fig. 5

Source Data Extended Data Fig. 3

Source Data Extended Data Fig. 4

## Figures and Tables

**Extended Data Table 1:**
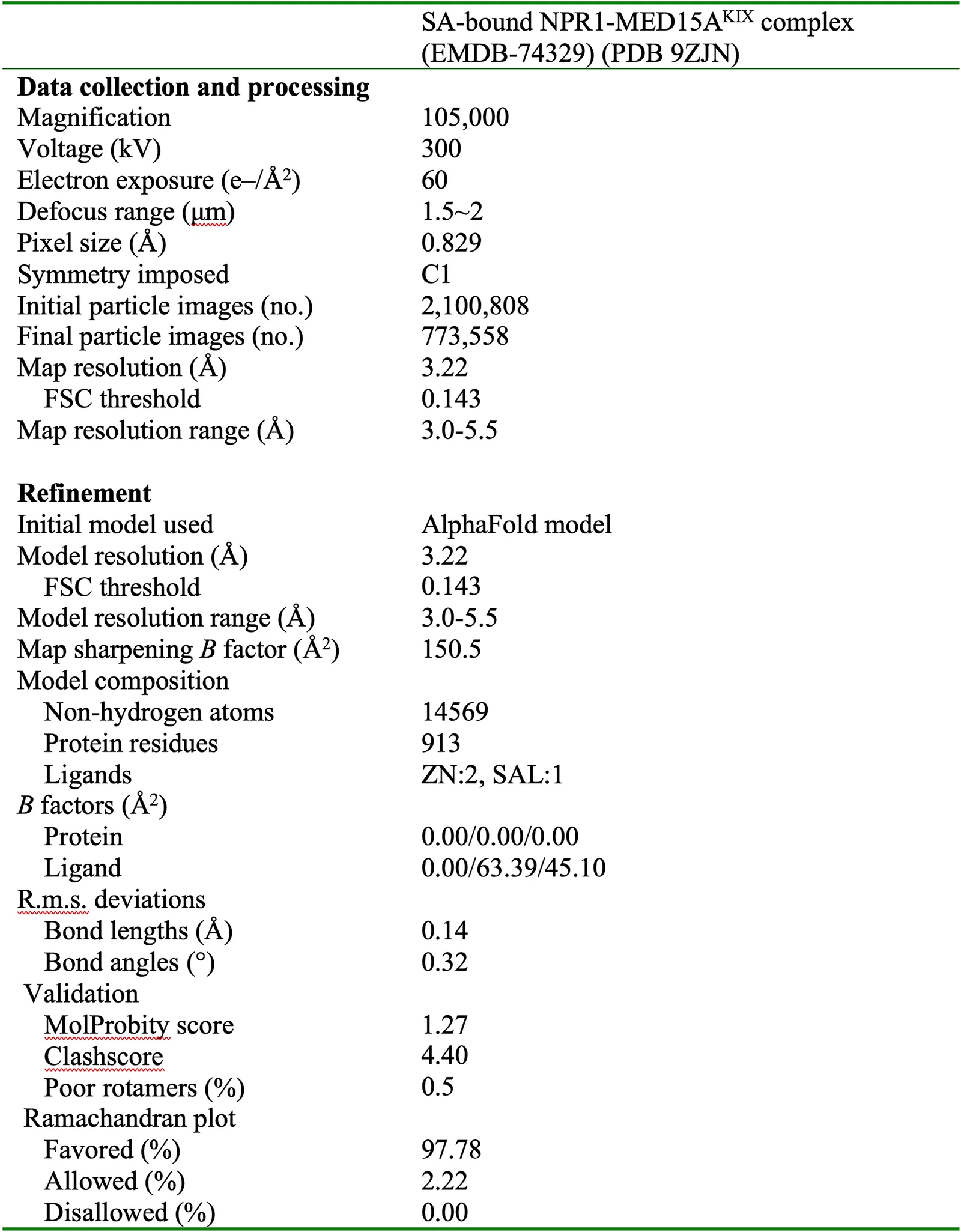
Cryo-EM data collection, refinement & validation statistics

**Extended Data Table 2:**
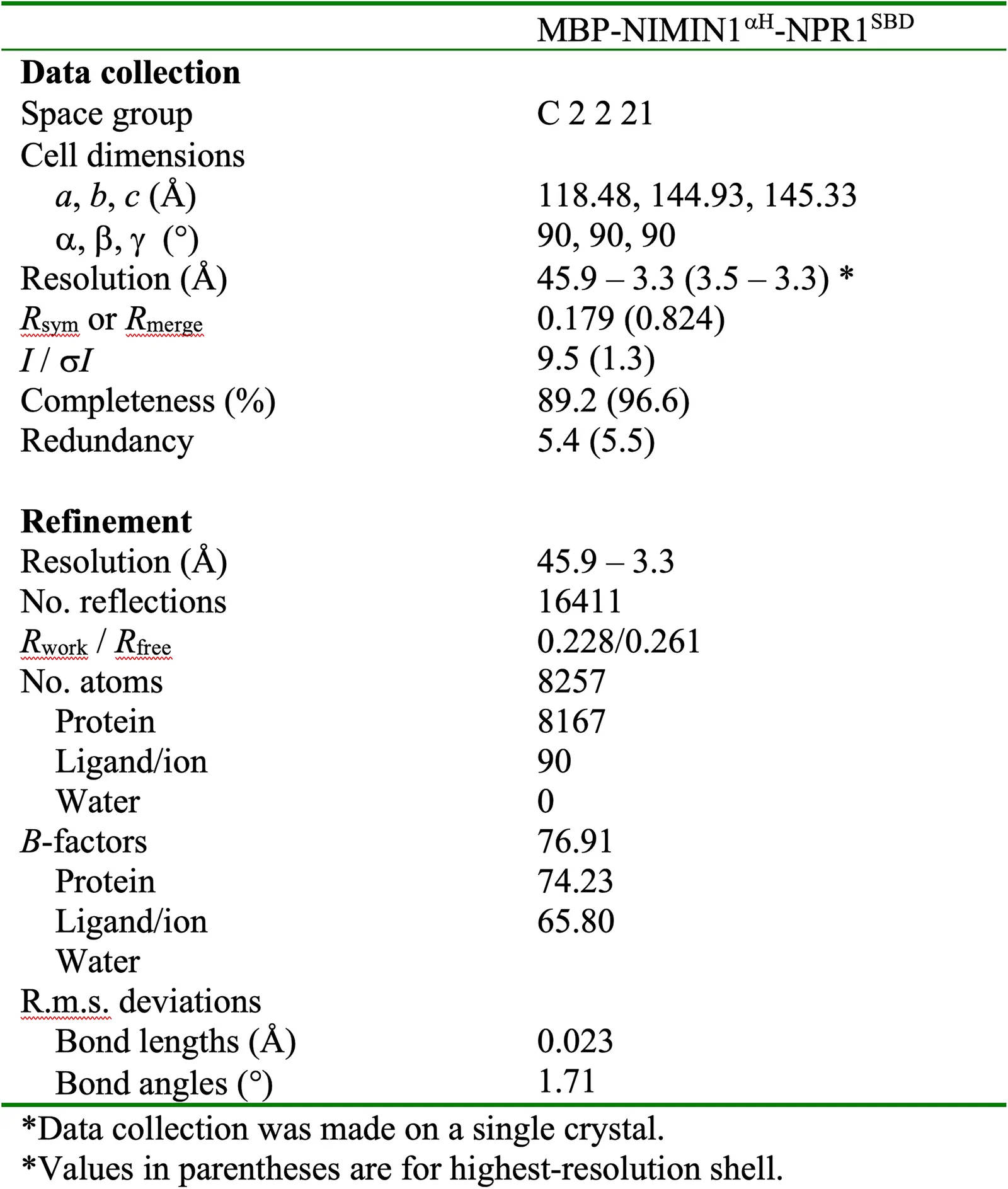
X-ray crystallography data collection and refinement statistics

## Data Availability

No custom code was developed for this study.

## Data availability

The coordinates and cryo-EM maps have been deposited in the Protein Data Bank and the Electron Microscopy Data Bank, respectively, with the following accession numbers: 9ZJN and EMD-74329 for NPR1–MED15A-KIX and 9ZM5 for NIMIN1–NPR1-SBD. The mass spectrometry data associated with HDX-MS have been deposited to the ProteomeXchange Consortium via the PRIDE partner repository with the dataset identifier PXD075340. Full versions of all gels and blots are provided in Supplementary Fig. 1. There are no restrictions on data availability. Source data are provided with this paper.
