# Supplementary material for: Transcriptional activation of plant immunity by salicylic acid
Source: Nature. 2026 Sep 16;659(8139):168–76. doi: 10.1038/s41586-026-11021-5 (PMC13736206; doi:10.1038/s41586-026-11021-5)

nature portfolio

https://doi.org/10.1038/s41586-026-11021-5

Supplementary information

Transcriptional activation of plant immunity by salicylic acid

In the format provided by the authors and unedited

Figure 1.

c

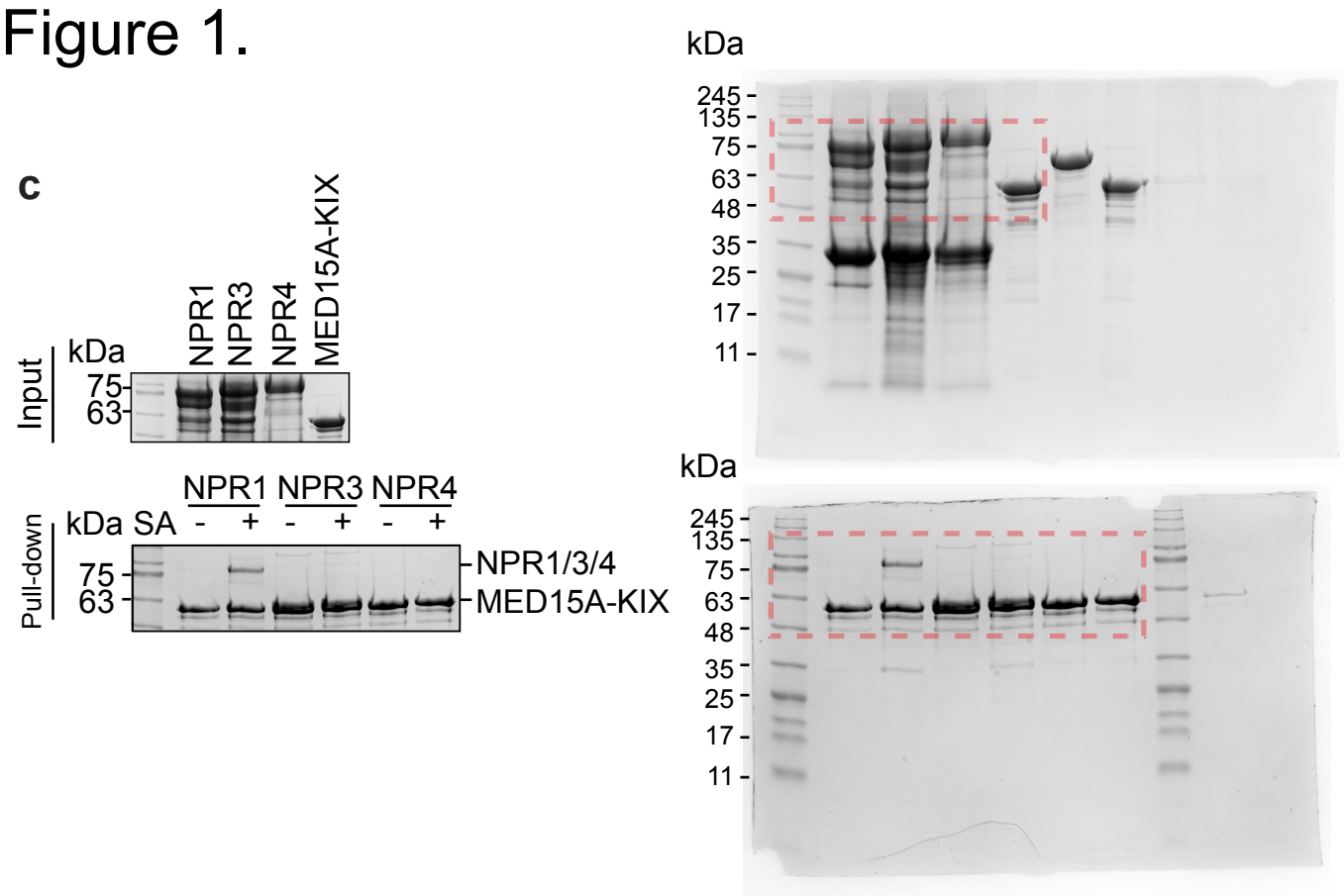

d

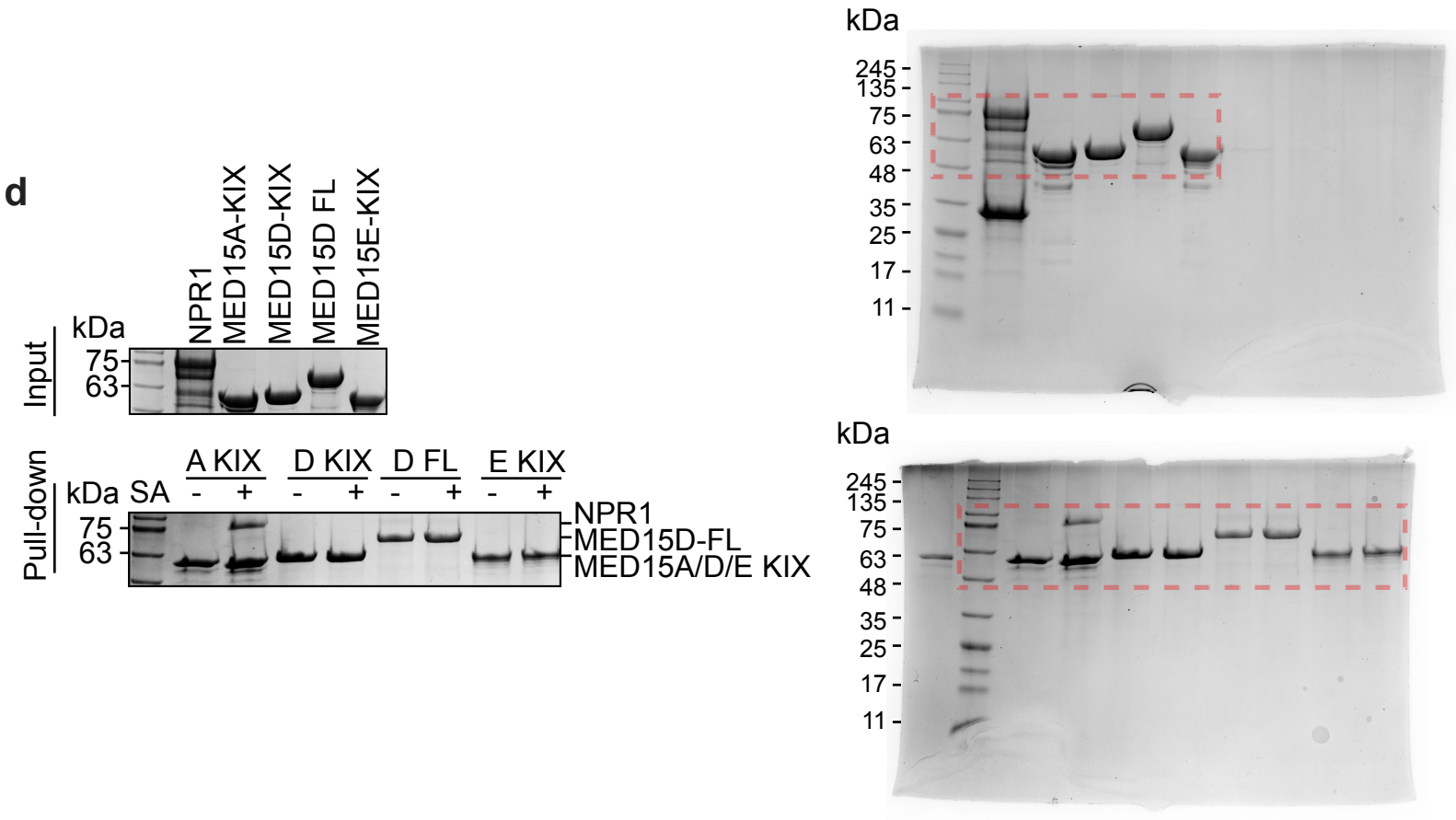

f

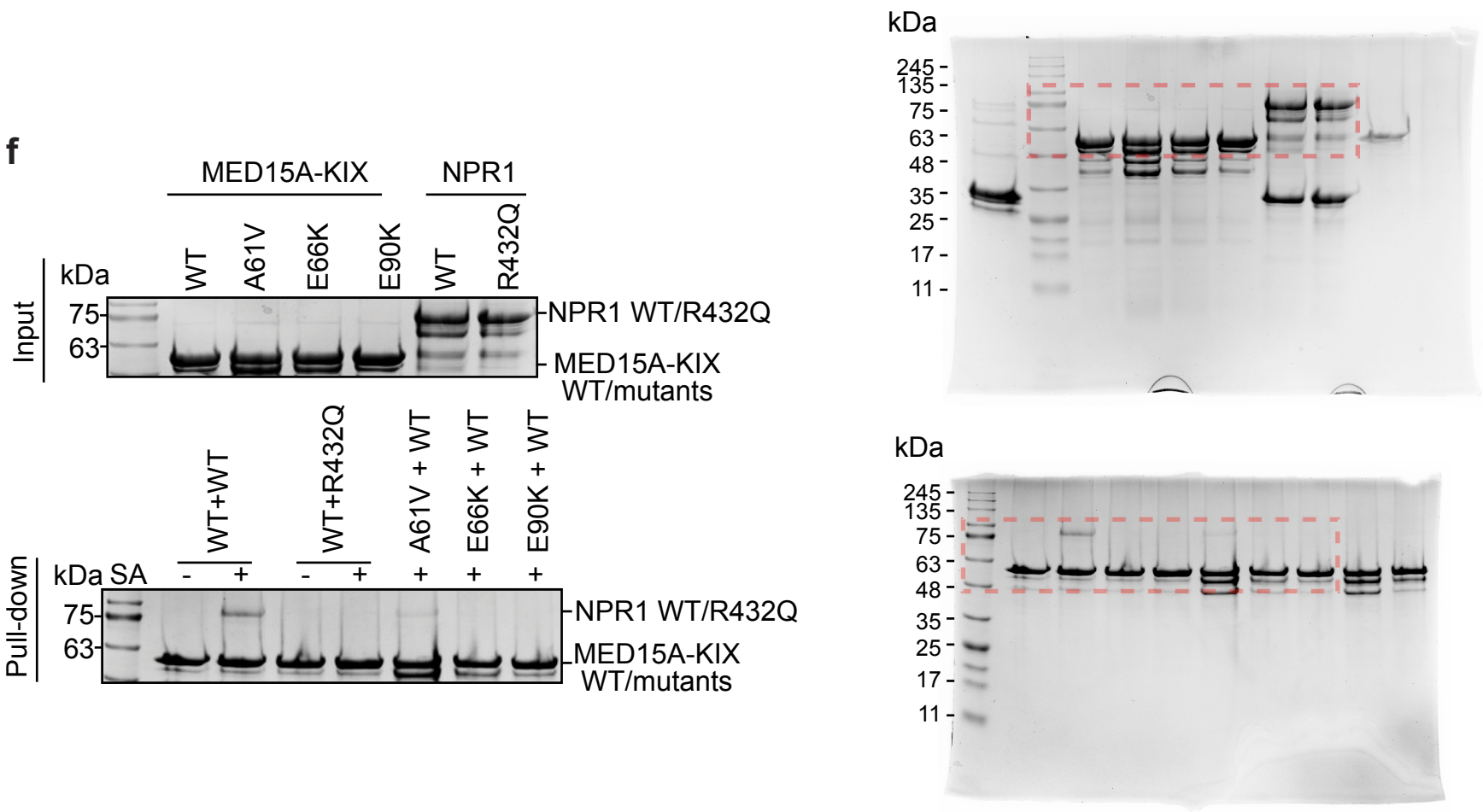

Figure 2.

a

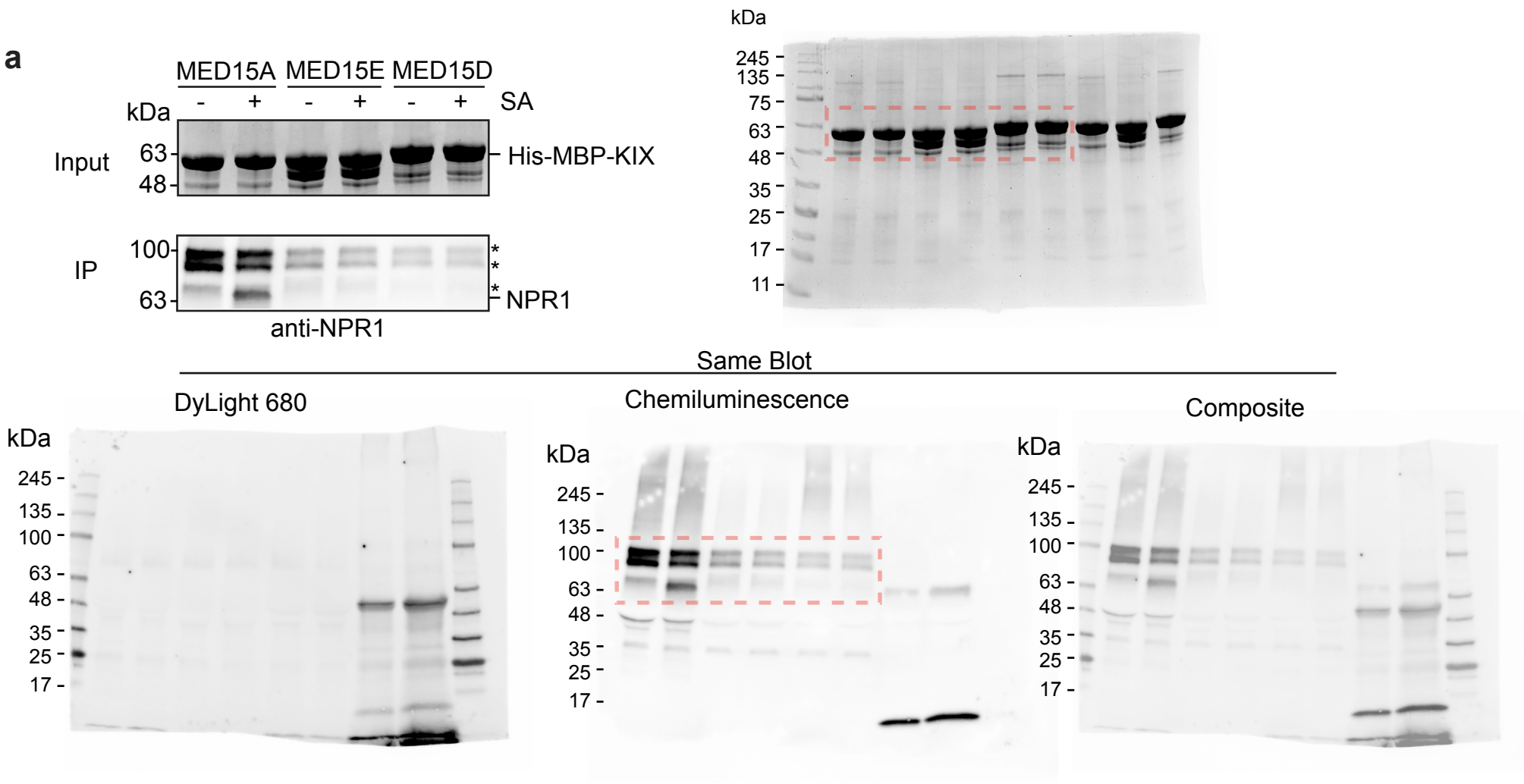

g

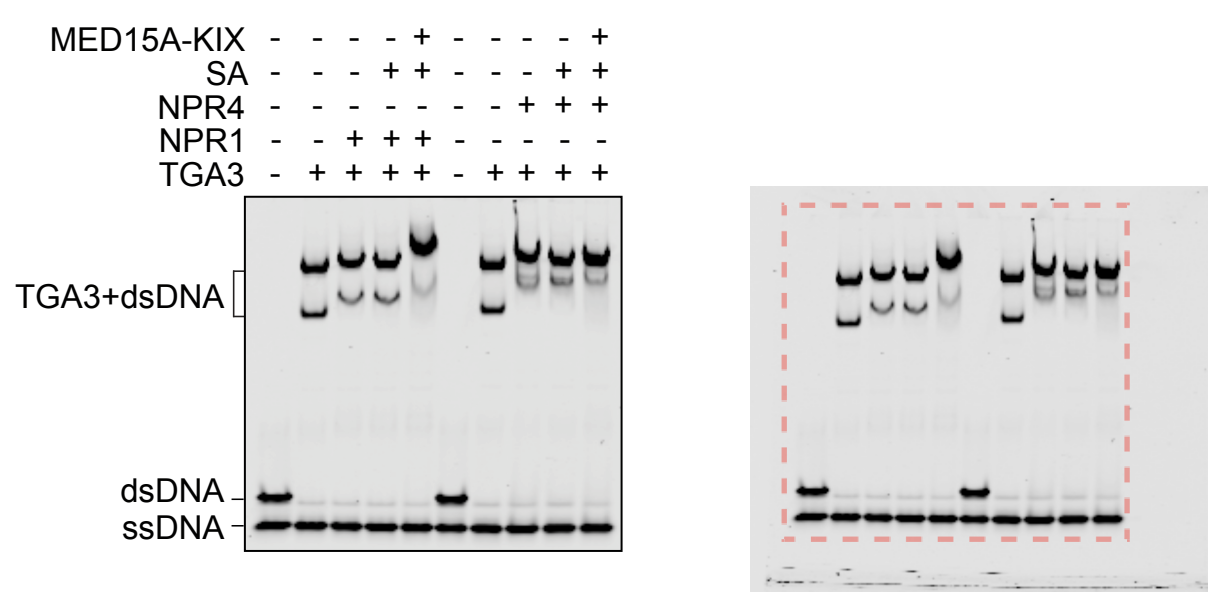

Figure 4.

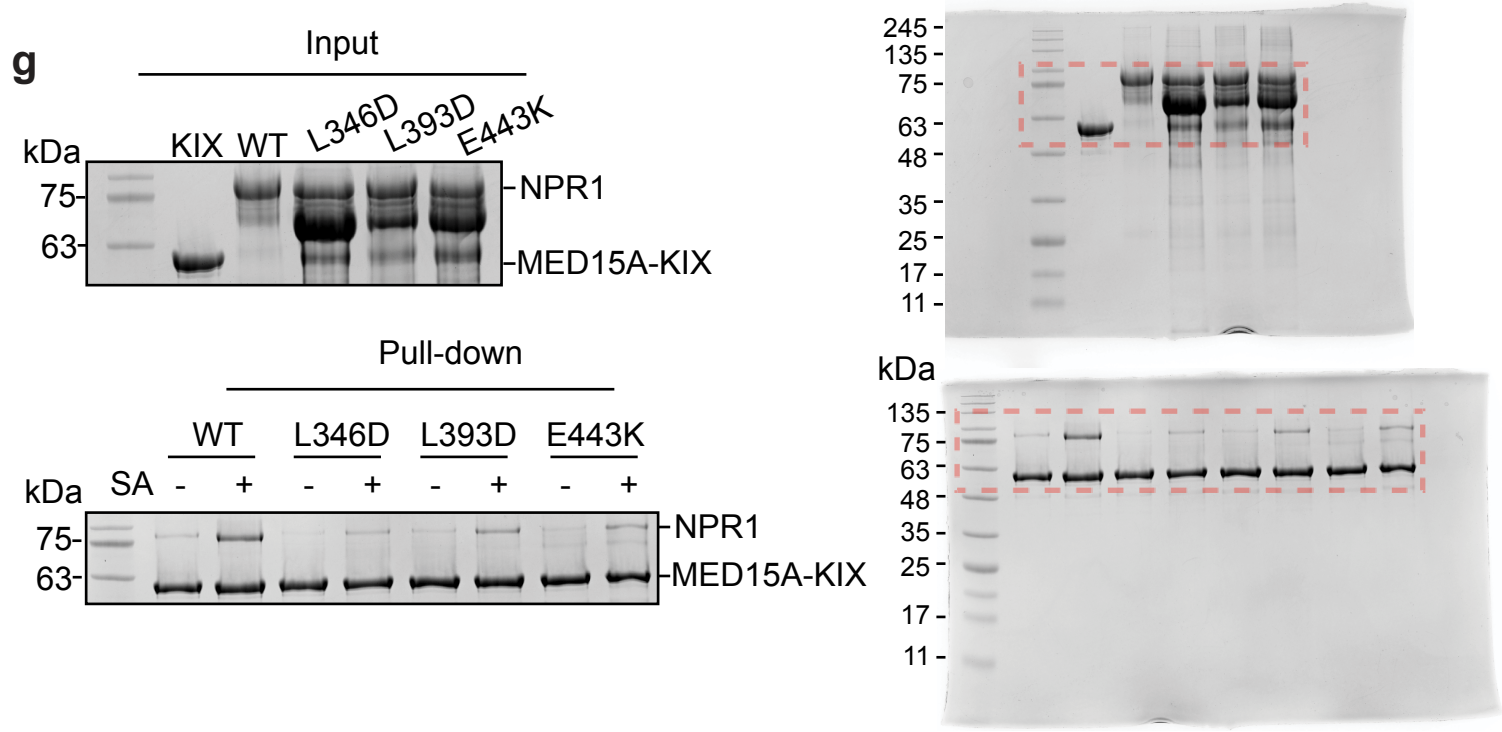

Extended Data Figure 1.

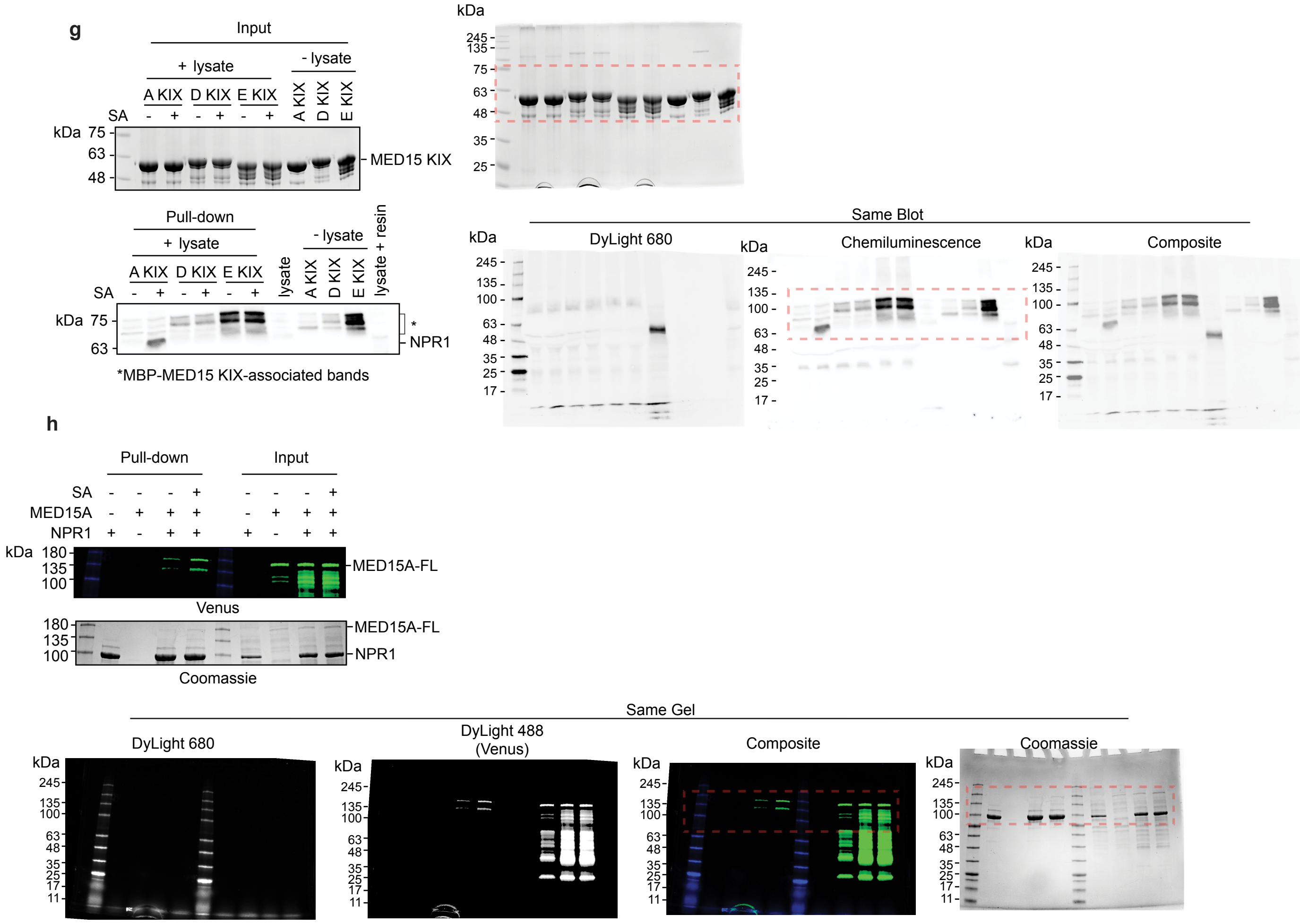

Extended Data Figure 2.

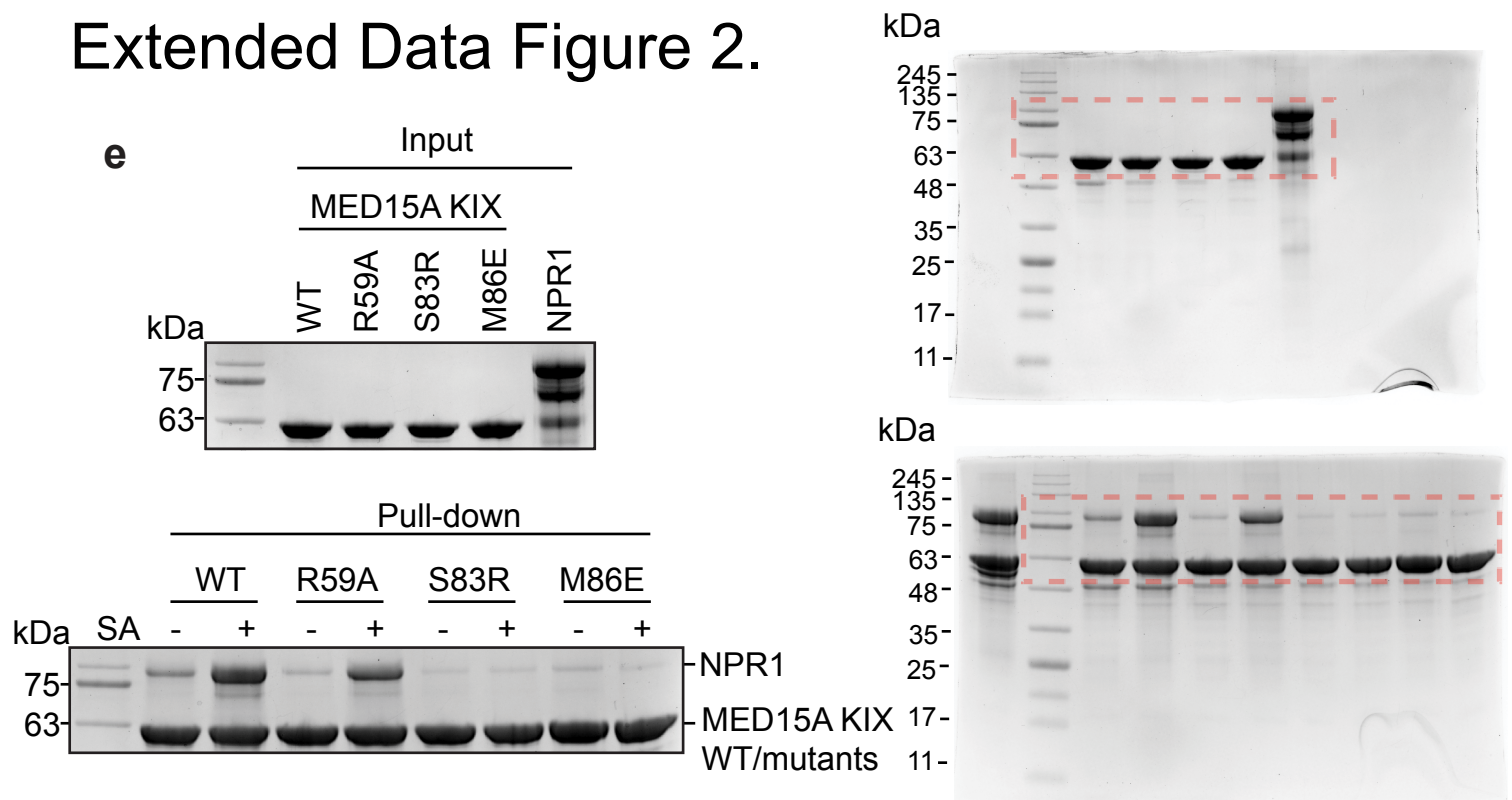

Extended Data Figure 2.

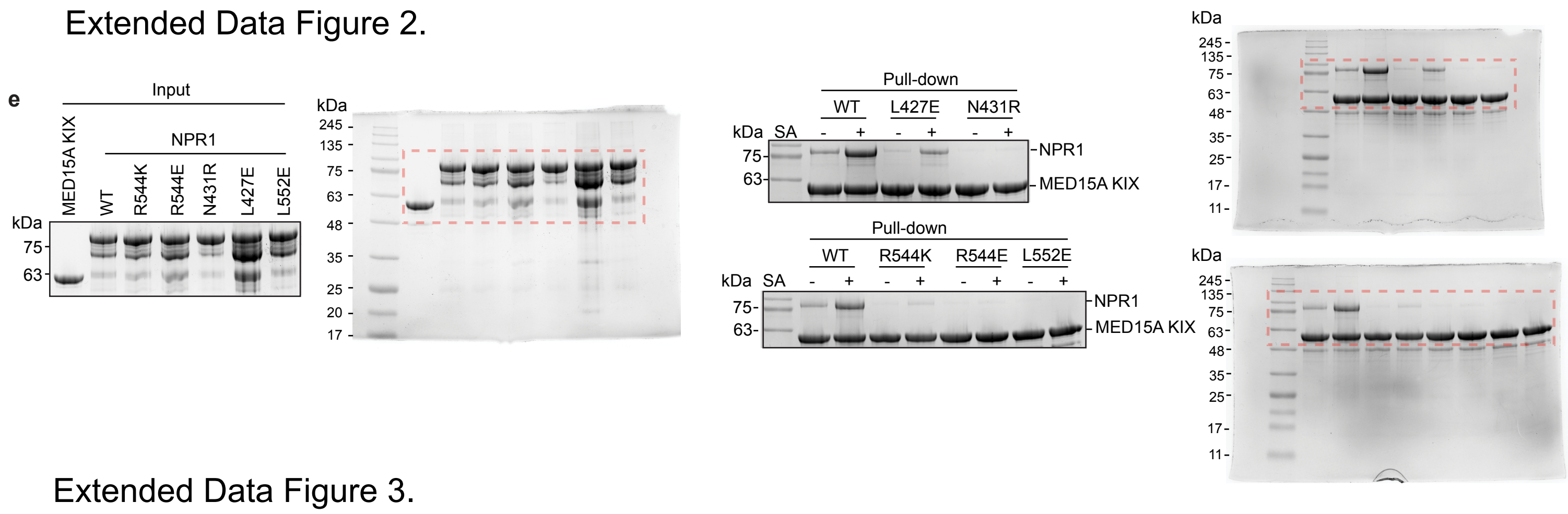

Extended Data Figure 3.

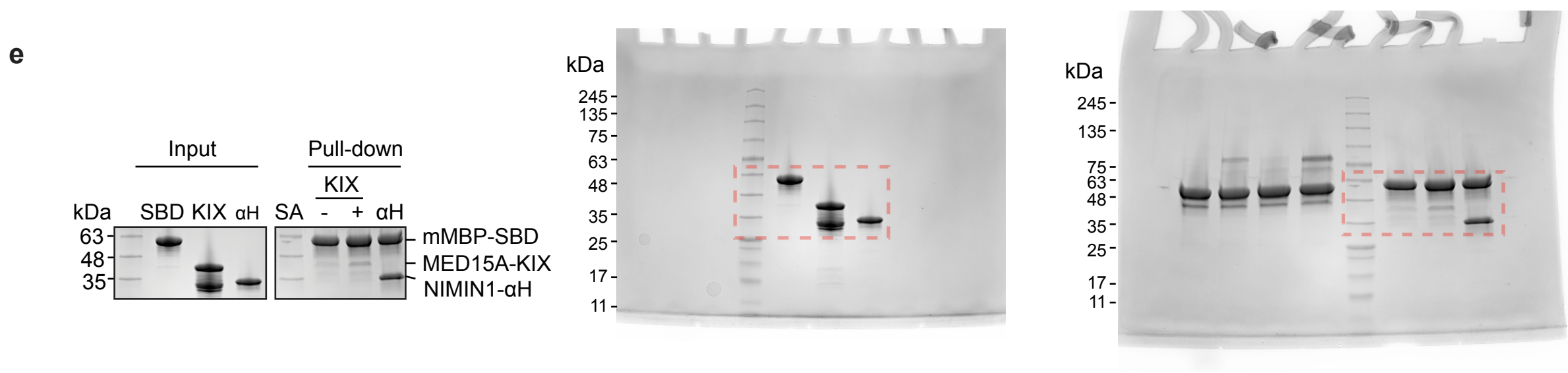

Extended Data Figure 4.

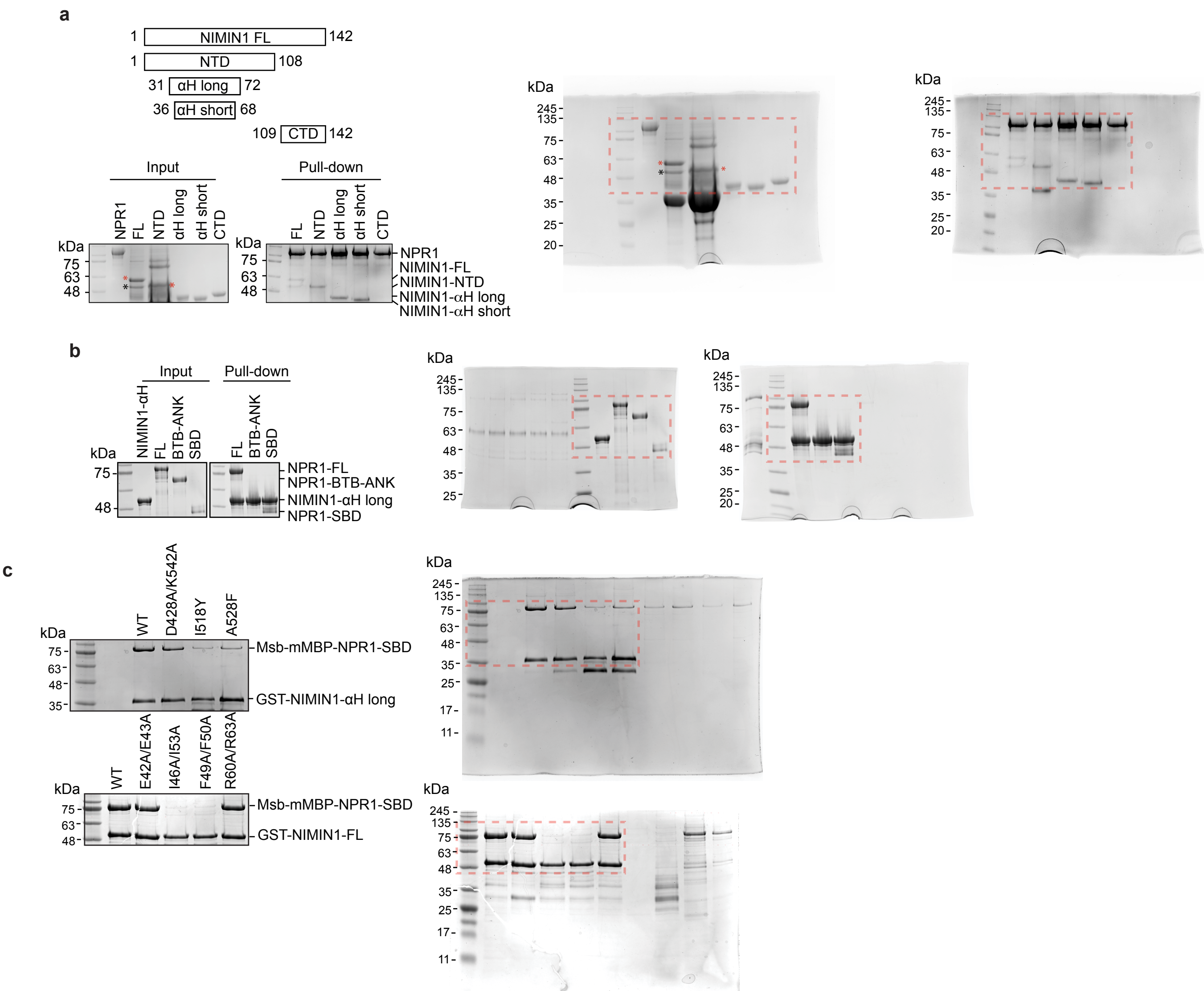

Supplement: Supplementary file 1 [file 41586_2026_11021_MOESM1_ESM.pdf]
