# Supplementary material for: Transcriptional activation of plant immunity by salicylic acid
Source: Nature. 2026 Sep 16;659(8139):168–76. doi: 10.1038/s41586-026-11021-5 (PMC13736206; doi:10.1038/s41586-026-11021-5)
Supplement: Supplementary file 2 [file 41586_2026_11021_MOESM2_ESM.pdf]

nature portfolio

Corresponding author(s): Songwen Zhang and Ning Zheng

Last updated by author(s): Jul 19, 2026

Reporting Summary

Nature Portfolio wishes to improve the reproducibility of the work that we publish. This form provides structure for consistency and transparency in reporting. For further information on Nature Portfolio policies, see our Editorial Policies and the Editorial Policy Checklist.

Statistics

For all statistical analyses, confirm that the following items are present in the figure legend, table legend, main text, or Methods section.

n/a	Confirmed	
☐	☒	The exact sample size (*n*) for each experimental group/condition, given as a discrete number and unit of measurement
☐	☒	A statement on whether measurements were taken from distinct samples or whether the same sample was measured repeatedly
☐	☒	The statistical test(s) used AND whether they are one- or two-sided<br>*Only common tests should be described solely by name; describe more complex techniques in the Methods section.*
☒	☐	A description of all covariates tested
☒	☐	A description of any assumptions or corrections, such as tests of normality and adjustment for multiple comparisons
☐	☒	A full description of the statistical parameters including central tendency (e.g. means) or other basic estimates (e.g. regression coefficient) AND variation (e.g. standard deviation) or associated estimates of uncertainty (e.g. confidence intervals)
☐	☒	For null hypothesis testing, the test statistic (e.g. *F*, *t*, *r*) with confidence intervals, effect sizes, degrees of freedom and *P* value noted<br>*Give P values as exact values whenever suitable.*
☒	☐	For Bayesian analysis, information on the choice of priors and Markov chain Monte Carlo settings
☒	☐	For hierarchical and complex designs, identification of the appropriate level for tests and full reporting of outcomes
☒	☐	Estimates of effect sizes (e.g. Cohen's *d*, Pearson's *r*), indicating how they were calculated

Our web collection on statistics for biologists contains articles on many of the points above.

Software and code

Policy information about availability of computer code

Data collection	Cryo-EM: Krios and Glacios Transmission Electron Microscope (Thermo Fisher) with K3 direct electron detector, operated on SerialEM (v4.1).<br>Protein structure prediction: AlphaFold 3 in the AlphaFold Server and Protenix in the Protenix Server.<br>Gel Imaging: Image Lab Touch software (v3.0.1.14) built in the ChemiDoc MP imaging system (Bio-Rad);<br>BLI data were collected with Octet BLI Discovery (v13.0.3.26).<br>ITC data were collected with MicroCal PEAQ-ITC Control Software (v1.41).<br>AlphaLisa: PerkinElmer EnSpire 2300 Multilabel Reader<br>Tritium-labelled SA membrane filter assay: Revvity QuantaSmartTM for Tri-Carb (v5.3)<br>BiFC imaging: Nikon Elements software (NIS-Elements AR, v5.20.01)<br>EMSA: Image Studio (v5.2) (LICORbioTM)<br>Hydrogen-deuterium exchange mass spectrometry: Byonic (Protein Metrics);<br>Crystallography: The X-ray diffraction data set was collected at the BL2.0.1 beamline at the Advanced Light Source in Berkeley and was integrated and scaled by XDS package.
Data analysis	Cryo-EM datasets were processed with CryoSparc (v4.4.1). The model building used crystal structure model as template, then docked in ChimeraX (v1.9) adjusted with Coot (v0.9.8.91), refined with PHENIX (v1.21.2-5419), and assessed with PyMol (v2.6.2).<br>BLI data was analyzed with Octet BLI Analysis (v12.2).<br>ITC data were analyzed with MicroCal PEAQ-ITC Analysis Software (v1.41).<br>BiFC images: Fiji ImageJ (v2.16.0/1.54p)<br>Hydrogen-deuterium exchange mass spectrometry: Deuterium incorporation was analyzed using HDExaminer (v3) (Trajan Scientific and Medical). Spectra displaying bimodal behavior were exported from HDExaminer and analyzed in HX-Express (v3).

Statistics analysis: GraphPad Prism (v10.6.1)
Gel band density quantification: Image Lab (v5.1) (Bio-Rad)

For manuscripts utilizing custom algorithms or software that are central to the research but not yet described in published literature, software must be made available to editors and reviewers. We strongly encourage code deposition in a community repository (e.g. GitHub). See the Nature Portfolio guidelines for submitting code & software for further information.

Data

Policy information about availability of data

All manuscripts must include a data availability statement. This statement should provide the following information, where applicable:

- Accession codes, unique identifiers, or web links for publicly available datasets
- A description of any restrictions on data availability
- For clinical datasets or third party data, please ensure that the statement adheres to our policy

The coordinates and cryo-EM maps have been deposited in the Protein Data Bank (PDB) and the Electron Microscopy Data Bank (EMDB) with the following accession numbers: NPR1-MED15A-KIX: 9ZJN, EMD-74329; NIMIN1-NPR1-SBD: 9ZM5. The mass spectrometry data associated with HDX-MS have been deposited to the ProteomeXchange Consortium via the PRIDE partner repository with the dataset identifier PXD075340. Full versions of all gels and blots are provided in Supplementary Fig. 1. There are no restrictions on data availability. Source data are provided with this paper.

Research involving human participants, their data, or biological material

Policy information about studies with human participants or human data. See also policy information about sex, gender (identity/presentation), and sexual orientation and race, ethnicity and racism.

Reporting on sex and gender	N/A
Reporting on race, ethnicity, or other socially relevant groupings	N/A
Population characteristics	N/A
Recruitment	N/A
Ethics oversight	N/A

Note that full information on the approval of the study protocol must also be provided in the manuscript.

Field-specific reporting

Please select the one below that is the best fit for your research. If you are not sure, read the appropriate sections before making your selection.

☒ Life sciences ☐ Behavioural & social sciences ☐ Ecological, evolutionary & environmental sciences

For a reference copy of the document with all sections, see nature.com/documents/nr-reporting-summary-flat.pdf

Life sciences study design

All studies must disclose on these points even when the disclosure is negative.

Sample size	For in vitro protein interaction assays (e.g., in-vitro pull-down and semi Co-IP) and biochemical binding assays (ITC, BLI, AlphaLISA, tritium-labelled SA membrane filter assay), all experiments were performed exclusively with purified recombinant protein complexes, and cell lysates in standard biochemical assays with sample sizes determined in previous studies (Shi et al. Nature 2026, Wang et al. Nature 2020, Cao et al. Nature Communication 2022 and Cao et al. Cell 2024). Cryo-EM sample size (e.g., number of micrographs) was as determined in (Shi et al. Nature 2026 and Cao et al. Cell 2024). EMSA sample size was chosen according to Kumar et al. (Nature 2022). For these biochemical binding assays, we ensured reproducibility by performing each assay in three technical replicates and confirming key findings with three independent replicates (including all the in vitro pull-down assays). For the in vivo BiFC confocal microscopy in tobacco, the sample size was determined as described in Kudla and Bock (Plant Cell 2016).
Data exclusions	No data were excluded in this study.
Replication	All experiments (except BiFC) were repeated three times with similar results. The BiFC experiments for the major samples/treatments (e.g., NPR1+MED15AKIX +-SA and NPR1+NIMIN1 +-SA) were repeated at least three times with similar results.
Randomization	Tobacco plants used for BiFC confocal microscopy were from the same batch of plants that grew in the same conditions in each independent replicate. Plants were assigned randomly for each condition/treatment. Otherwise, randomization is not applicable to biochemical assays. All biochemical experiments were performed using purified recombinant proteins, and standard cell culture systems used solely for protein expression. These experimental systems do not involve allocation of subjects, biological groups, or treatments. Instead, reproducibility was ensured by performing biochemical and biophysical assays in independent replicates, which is the standard practice for mechanistic protein

studies.

Blinding

Investigators were not blinded for any of the experiments performed in this study. This was done for practical purposes, and is standard practice for studies employing biochemistry, cell culture, and genomics (Zhang et al., 2018; Vinyard et al., 2019; Haggerty et al., 2021).

Reporting for specific materials, systems and methods

We require information from authors about some types of materials, experimental systems and methods used in many studies. Here, indicate whether each material, system or method listed is relevant to your study. If you are not sure if a list item applies to your research, read the appropriate section before selecting a response.

Materials & experimental systems

n/a	Involved in the study
☐	☒ Antibodies
☐	☒ Eukaryotic cell lines
☒	☐ Palaeontology and archaeology
☒	☐ Animals and other organisms
☒	☐ Clinical data
☒	☐ Dual use research of concern
☐	☒ Plants

Methods

n/a	Involved in the study
☒	☐ ChIP-seq
☒	☐ Flow cytometry
☒	☐ MRI-based neuroimaging

Antibodies

Antibodies used

anti-NPR1: a gift from Dr. Zhonglin Mou from University of Florida. It was originally custom developed for Dr. Mou by Thermo Fisher Scientific.
anti-Rabbit IgG Horseradish Peroxidase: Amersham™ ECL™ Rabbit IgG, HRP-linked whole antibody, from donkey, Cytiva, catalog number NA934V, Lot number 18408666.

Validation

This antibody has been validated by Dr. Zhonglin Mou and many other researchers and reported in the following publications: 1) Ding et al. 2016, PMID: 26865090; 2) Ding et al. 2020, PMID: 33072146. We also validated this antibody by detecting endogenous NPR1 in water-treated and SA-treated Arabidopsis leaf lysate, where NPR1 level was extremely low and barely detectable in water-treated leaves but much higher and more readily detectable in the SA-treated samples.
Anti-Rabbit IgG: https://www.cytivalifesciences.com/en/us/products/items/amersham-ecl-hrp-conjugated-antibodies-p-06260

Eukaryotic cell lines

Policy information about cell lines and Sex and Gender in Research

Cell line source(s)

Sf9 and Hi5 monolayer insect cell lines were purchased from Thermo Fisher Scientific.

Authentication

All cell lines were authenticated by Short Tandem Repeat profiling (Genetica).

Mycoplasma contamination

All cell lines tested negative for mycoplasma.

Commonly misidentified lines
(See ICLAC register)

No commonly misidentified cell lines were used.

Plants

Seed stocks

Arabidopsis thaliana wild type Col-0 seed stock was purchased from TAIR (The Arabidopsis Information Resource)(assession number: 1008804532); Tobacco (Nicotiana benthamiana) wild type seed stock was a gift from Dr. Takato Imaizumi at University of Washington.

Novel plant genotypes

No novel plant genotypes were developed/used in this study.

Authentication

N/A
