# Supplementary material for: Transcriptional activation of plant immunity by salicylic acid
Source: Nature. 2026 Sep 16;659(8139):168–76. doi: 10.1038/s41586-026-11021-5 (PMC13736206; doi:10.1038/s41586-026-11021-5)
Supplement: Supplementary file 3 [file 41586_2026_11021_MOESM4_ESM.pdf]

nature portfolio

Peer Review File

Transcriptional activation of plant immunity by salicylic acid

Corresponding Author: Professor Ning Zheng

Any redactions in this file are there to maintain patient confidentiality, the confidentiality of unpublished data, or to remove third-party material.

This file contains all reviewer reports in order by version, followed by all author rebuttals in order by version.

Version 0:

Reviewer comments:

Referee #1

(Remarks to the Author)
Salicylic acid (SA) is a plant defense hormone that plays a critical role in establishing local and systemic resistance. NPR1 functions as a receptor for SA to positively regulate SA-induced immune gene expression, whereas NIMIN1 is an NPR1-interacting protein that acts as a transcriptional repressor. Despite its established roles in plant immunity, the molecular mechanisms by which NPR1 modulates SA signaling remain incompletely understood. By leveraging AlphaFold predictions, this study identifies the KIX domain of MED15A as a specific NPR1-binding partner, a component which was previously shown to be genetically important for SA signaling. This interaction is validated biochemically and found to be SA-dependent. The authors further determine a cryo-EM structure of NPR1 bound to the MED15A KIX domain, revealing that the KIX domain binds exclusively to the SA-binding domain of NPR1 (NPR1-SBD). Using hydrogen-deuterium exchange mass spectrometry (HDX-MS), they show that SA binding robustly stabilizes the SA-binding core (SBC) within NPR1-SBD, elucidating the mechanism underlying SA-induced NPR1–MED15A-KIX interaction. They also solve the crystal structure of MBP-fused NPR1-SBD in complex with NIMIN1. Structural comparison reveals that NIMIN1 not only sterically occludes the MED15A-KIX binding site on NPR1-SBD but also remodels the SA-binding pocket. Together, these findings demonstrate that SA-bound NPR1 and the Mediator complex together function as a transcriptional co-activator, filling a critical gap in our understanding of SA-induced immune signaling and representing a significant advance in the field of plant immunity. The experiments were well performed, the data are solid, and the conclusions are well supported. The manuscript is clearly written and is a strong candidate for publication in Nature.

Major comments:
1. As mentioned in the introduction, the NPR1 paralogs NPR3 and NPR4 also function as SA receptors but negatively regulate SA-responsive genes. Based on the structural insights into the NIMIN1-NPR1 interaction, can the authors predict and test whether NIMIN1 interacts with NPR3 and NPR4? NPR3, NPR4, and an NPR4 mutant (R419Q) that loses SA binding but retains the ability to interact with TGA2 and form homodimers have been successfully purified (Ding et al., Cell, 2018). It would be straightforward to test these interactions.
2. ITC was used to quantify the SA-binding affinity of NPR1 in the presence of the MED15A-KIX domain. However, under these conditions, the measured heat signal arises from both SA binding to NPR1 and the subsequent binding of MED15A-KIX to SA-bound NPR1, rather than SA binding alone. This complicates the interpretation of the apparent affinity.

Minor comments:
1. Lines 149–150: “inclusion of MED15A-KIX enhanced the SA-binding affinity of NPR1 to ~100 nM (Fig. 2e)” The actual value in Fig. 2e is ~119 nM and should be corrected accordingly.
2. Line 221: “CTA helix is simultaneously engaged with the αK1 and αK2 helices of MED15A-KIX: This should read “αK1 and αK3 helices,” based on the structural data.
3. Line 418: “full-length MED15C” — this should likely be “full-length MED15D,” as MED15D is the isoform discussed in the relevant context.

Referee #2

(Remarks to the Author)
Using AlphaFold predictions, the authors identified the Mediator component MED15A as a candidate protein interacting with the salicylic acid (SA) receptor NPR1. MED15A was genetically found as a crucial SA signalling component and part of the NPR1 proxitome before. In this study, in vitro pull-down assays, bio-layer interferometry and AlphaScreen assays revealed that SA increases the binding affinity between NPR1 and the N-terminal KIX domain of MED15A. This result was validated in vivo by bimolecular fluorescence complementation (BiFC) assays. With isothermal titration calorimetry and radio-labelled ligand binding assays, the authors additionally demonstrated that the presence of MED15A-KIX enhances the SA-binding affinity of NPR1. An electrophoresis mobility shift assay showed the in vitro formation of a TGA3-NPR1-MED15A KIX complex being formed at the TGA3 target elements of the PR1 promoter.
The authors performed cryo-electron microscopy of SA-bound NPR1 in complex with the MED15A KIX domain as well as hydrogen-deuterium exchange mass spectrometry to show cooperative binding of SA and MED15A-KIX to NPR1.
In the last section of this manuscript, the molecular function of the NPR1-interacting protein NIMIN1, a known transcriptional repressor was analysed. The crystal structure of the NPR1 SA-binding domain (SBD, fused to the maltose binding protein) in complex with a NIMIN1 fragment, AlphaScreen-based competition assays and BiFC analyses provided evidence for an inhibition of the NPR1-MED15A-KIX interaction by NIMIN1. Radio-labeled ligand binding assay demonstrated that SA-binding to NPR1 is abrogated in the presence of the NIMIN1 peptide.
Altogether, the authors presented a model consisting of an SA-bound NPR1 dimer attached to two TGA3 homodimers and two MED15A-KIX domains bridging to the Mediator complex for SA-induced transcriptional activation of NPR1/TGA-dependent target promoters.

It was so far unknown how binding of SA to NPR1 enhances its transcriptional activation capacity. Here, Zhang et al. present comprehensive analyses of the structure and the cooperative binding kinetics of a complex consisting of NPR1, SA and the MED15A KIX domain.
I have to admit, that I am not a structural biologist, so it is difficult for me to judge e.g. the quality of the cryo-EM and crystallisation data, but I think the paper is of high interest in the plant-pathogen field.

However, I have some remarks:

Figure 1c: NPR3 and NPR4 fusions migrate a little bit slower than NPR1. Why has the pull-down western image been cut directly above the NPR1 band? Most likely, there are no or just faint signals but the upper border of the red box on the full-size membrane might hide signals.
How was the treatment with salicylic acid done, which concentration has been used?

Figure 1d: faint NPR1 band obtained with MED15D FL.
Lane 110: Why has the MED15D-FL protein been used, why not MED15A-FL? In the original pull-down western, a faint NPR1 band seems to be visible.

Lane 121: “screens8,25“ has to be corrected to screens18,25 (wrong reference)

Lane 156 and lane 445: a control for TGA3/NPR1/MED15A-KIX without SA is missing for the statements “SA-bound NPR1 links Mediator to TGA-DNA” and “SA-mediated recruitment of MED15A-KIX to......”

Lanes 327,328: NPR1+MED and NPR1+MED in the presence of NIMIN1 hasn’t been performed within one experiment.

Lane 431: Stars in Figure 2a?

Lane 434: BiFC assays with ASK1 are just quantified in Figure 2c, corresponding images are shown in Extended Data Fig. 1g.

Lane 435: Avoid “endogenous”. RFP is an internal marker serving as control for transformation and expression efficiency.

Lane 489ff, Figure 5: size of the NIMIN1 αH peptide (short?) is missing

Lane 548: Extended Data Fig. 3: The mirror plot is a perfect mirror image. The “+SA datapoints are copied from Figure 4a.

Lane 741: MED15D-FL? In Figure2b, MED15D-KIX is indicated.

Lanes 741, 742: ASK1 has not been used for the in vitro pull-down.

Figure 3d: two αSC1 label

Extended Data Fig. 3f: Why not 50 µM SA as used in Figure 1e?

Figure 4g: less input protein for the mutated NPR1s

Extended Data Fig. 4b: BTB-ANK input amount low

The manuscript contains many sloppy errors and spelling mistakes, especially in the material and methods section. Just a

few examples: Lane 97 “Polymerase” instead of polymerase. Lane 278: MED15A instead of “MED15”. Lane 516 “plot” instead of plots. Lane 752 “QiaGen” instead of Qiagen. Lane 650: Sentence “As His-Venus-tagged NPRs.....”. Lane 682: “AlphaSreen”. Lane 749 “For ASK1, used XbaI and PstI.” Lane 794 “petal” instead of pestle.
Not all abbreviations are explained (e.g. lane 608, “mMBP” or lane 613, “Msb”)

Referee #3

(Remarks to the Author)
MED15A, a recently identified proximal component of NPR1, plays an indispensable role in mediating NPR1-dependent transcriptional activation within the SA signaling pathway. In addition, NIMIN1 has long been known to associate with NPR1 and negatively regulate SA responses. In this study, Zhang et al. demonstrate that the the KIX domain of MED15A directly interacts with NPR1 in an SA-dependent manner and provide structural insights into this interaction. Furthermore, they structurally elucidate the NPR1–NIMIN1 interaction, which contributes to a better understanding of its antagonistic role in NPR1-mediated SA signaling. The current manuscript has several issues that need to be addressed.

Major points
1.In this study, the authors only show interaction between NPR1 and the isolated KIX domain of MED15A. However, it is the full-length MED15A—not the KIX fragment—that functions in planta. Thus, all subsequent analyses based solely on the KIX domain remain questionable. Moreover, potential artifacts introduced by using only the KIX domain cannot be excluded. Therefore, the interaction and structural analyses between NPR1 and MED15A should be verified using full length of MED15A protein.
2.The E90K mutation is a known loss-of-function, SA-insensitive allele identified in previous genetic screens. Although MED15A-KIX-E90K shows reduced interaction with NPR1 compared with wild-type MED15A-KIX, it still associates with NPR1 in vivo upon SA treatment (Fig. 2b). This observation is inconsistent with previous genetic evidence and the in vitro interaction data shown in Fig. 1f, and therefore requires further clarification.
3.It appears that only one MED15A KIX domain shows electron density in the 2:2 symmetric NPR1-SA-MED15A-KIX dimer claimed by the authors (Ext Fig 2a). Additionally, the authors performed chemical cross-linking on the purified NPR1-SA-MED15A-KIX complex (Line 857). Therefore, the 2:2 dimeric complex is largely inferred by the authors (Fig 3a), clarification on this point is requested.
4.The A61V mutation in MED15A has been reported previously to disrupt MED15A function. However, the structural analyses presented here do not provide mechanistic insights into how this mutation impairs MED15A activity (Lines 236–240).
5.Structural analyses indicate that MED15A-KIX does not contact the ANK domain of NPR1. It is therefore unclear why the isolated NPR1 SBD domain fails to interact efficiently with MED15A-KIX in the presence of SA when the ANK domain is absent.
6.To elucidate the NPR1–NIMIN1 interaction, it would be preferable to use full-length NPR1 and NIMIN1 proteins, rather than relying solely on an NPR1 SBD domain and a truncated NIMIN1 fragment.
7.Since SA disrupts the NPR1-NIMIN1 interaction, the NPR1-MED15A interaction would be expected to be enhanced upon SA treatment in the BiFC assays (Fig. 5h, i), even in the presence of NIMIN1 co-expression. This point requires clarification or additional experimental support.
8.In the BiFC assays, NIMIN1 should competitively bind to YFPN-fused NPR1 against YFPC-fused NIMIN1, thereby resulting in a weaker interaction signal compared to when NIMIN1 is not co-expressed with YFPC-fused NIMIN1 and YFPN-fused NPR1. However, the quantitative interaction signals presented in Fig. 5i appear to contradict this assertion. It is requested to either clarify this discrepancy or provide complementary in vivo interaction assays to validate their findings.
9.It is recommended that the physiological functions of the interaction interfaces within the NPR1-MED15A KIX and NPR1-NIMIN1 protein complexes should be validated by introducing targeted point mutations at the interfaces, followed by transcriptional activity assays in plants or phenotypic observation in transgenic plants.
10.SA is embedded within the center of the NPR1-SBC four-helix bundle (Line 196) and therefore cannot directly contact MED15A-KIX. Hence, referring to SA as a "glue" here is misleading. Although the authors attempt to describe both SA and gibberellin as "intramolecular glues" (Line 359), this description remains highly susceptible to misinterpretation, as readers may assume that SA, much like a conventional "glue", simultaneously contacts both NPR1 and MED15A-KIX to bridge them, whereas in reality SA only directly binds to the receptor NPR1. Therefore, the term "glues" in the title "Salicylic Acid Allosterically Glues NPR1 and Mediator to Activate Plant Immunity" is also recommended to be revised.

Minor points
1.In Fig. 2a, the two slower-migrating bands above the labeled NPR1 in the IP samples are likely non-specific proteins pulled down during immunoprecipitation. However, it is unclear why the abundance of these non-specific bands differs among the MED15A/D/E samples.
2.Line 186: “discernible” (spelling).
3.Line 232: “CTH” is introduced without definition. Should this be the C-terminal helical arm (CTA) in Line 194 and the C-terminal arm (CTA) in Line 471?

Referee #4

(Remarks to the Author)
I co-reviewed this manuscript with one of the reviewers who provided the listed reports.

Referee #5

(Remarks to the Author)
The manuscript by Zhang and coworkers outlines the allosteric mechanism underlying SA binding to its receptor, NPR1, and the concomitant activating effect on MED15A binding. The authors approach this using a series of technically sound and well-designed experiments, starting with pull-down assays and immunoprecipitation of whole-cell lysates to demonstrate this interaction, with appropriate controls including other KIX domain-containing MED15 paralogs and MED15A variants. They further show cooperative binding of SA and MED15A using AlphaScreen competition assays, ITC, and BLI. Cryo-EM structures and HDX-MS analyses provide detailed and convincing structural information characterizing this interaction and explaining the loss-of-function mutants observed in genome libraries. Additionally, the work demonstrates how the negative feedback regulator, NIMIN1, binds NPR1 to displace MED15A. Overall, this study provides mechanistic insight into how SA-mediated gene regulation is facilitated. This is an interesting study that merits publication in Nature once the following minor points are addressed.

Points to address:
1) "Such an SA-dependent NPR1-interacting protein might also help explain the inconsistent SA-binding activity of NPR1, which varies substantially depending on the methods employed for protein purification and ligand-binding measurement."

Is there a reference to support this claim?

2) "In addition to NIMIN1, which has been shown to directly interact with NPR1"

Please provide a citation for this statement.

3) "To identify the missing factor that hypothetically interacts with SA-bound NPR1, we performed AlphaFold predictions of the SA receptor in complexes with individual NPR1-proximal proteins recently revealed by proximity labeling experiments17."

The claim is that NPR1 binds MED15A in an SA-dependent manner. Were these AlphaFold predictions performed with SA included?

4) Figure 1G. What is the error associated with the fitted values shown?

5) "AlphaFold predictions indicate that the Arabidopsis MED15A CTD engages in analogous interactions with its partners, MED14 and MED23 (Extended Data Fig. 1i)."

What is the confidence level of this prediction? No data are provided to suggest that this is a robust interaction beyond a visual inspection of the predicted structure.

6) Figure 4C - HDX data: The figure scale should be quantitative, showing either ΔDa or Δ% deuterated rather than using qualitative labels such as "more protected."

7) "The apparent unfolding rate is approximately 10-fold slower with MED15A-KIX bound, further indicating that the MED15A interaction stabilizes the helices within the SBD."

The authors should show how this 10-fold slower exchange rate was determined using a figure panel.

8) Before discussing HDX results, a sentence or two should briefly explain what reduced deuterium uptake means in the context of protein structure to make the section more interpretable to a broad audience. Also please consider briefly expanding on the HDX-MS workflow in the main text, including mention of the continuous-labeling strategy.

9) Please include the HDX summary table as per "Masson, G.R., Burke, J.E., Ahn, N.G. et al. Recommendations for performing, interpreting and reporting hydrogen deuterium exchange mass spectrometry (HDX-MS) experiments. Nat Methods 16, 595–602 (2019). https://doi.org/10.1038/s41592-019-0459-y" The HDX data deposition entry also needs to be updated. It is currently written as XXXXX.

10) Given the title, "Salicylic Acid Allosterically Glues NPR1 and Mediator to Activate Plant Immunity," the concept of SA as a "molecular glue" should be introduced much earlier in the discussion. Additionally, the paper does not provide direct evidence that SA promotes NPR1 interaction with the entire Mediator complex, but rather only with MED15A, which is a component of Mediator. The title should be revised to reflect this specificity more accurately.

Version 1:

Reviewer comments:

Referee #1

(Remarks to the Author)
The revision has satisfactorily addressed my previous concerns. I support publication of the revised version of the

manuscript in Nature.

Referee #2

(Remarks to the Author)
The authors have adequately revised the manuscript in response to my comments. For the resubmitted version, I have only a few remaining points:

It is not necessary to include Figure R2, but the statement of the authors in their rebuttal letter "…at such a high concentration, MED15AKIX by itself starts to shift the TGA-NPR1 associated DNA, but not free DNA..." is not correct. Lane 5 (MED14AKIX alone plus DNA) shows a clear band shift!!!

Lane 538 should be corrected to: As encoded by an SA-responsive gene, NIMIN1..

Extended Data Fig. 1h: How did the authors detect Venus fluorescence? A standard SDS-PAGE protocol disrupts the protein's structure thereby causing loss of its fluorescence.

Referee #3

(Remarks to the Author)
In this revised version, the authors have provided new data that have basically addressed my previous concerns.

In the title, given that NPR1 specifically interacts with MED15A but not other Mediator subunits (e.g., MED15D/E), it is recommended that the authors substitute 'Mediator-15' directly with 'MED15A'.

Additionally, this manuscript still contains some minor errors, examples are shown as below:

1)Line 55, insert 'the' before 'sequence level'.
2)Line 59, insert 'assumed' before 'opposing mechanisms', as the detailed mechanisms by which NPR3/4 modulate transcription have not been elucidated previously, or in this manuscript. Similarly, in line 204.
3)Line 63, the full name of TGA.
4)Line 106, pull-down.
5)Line 123, please superscript KIX in 'NPR1-MED15A-KIX', do not hyphenate it with MED15A.
6)Line 142-158, I realize that MED15A stabilizes the binding of NPR1 with SA, but the phrasing like 'recruitment of MED15A-KIX sensitizes NPR1 for binding SA' may be misleading. It would benefit from some revision, for example: recruitment of MED15A-KIX by SA-bound NPR1 further enhances its SA sensitivity.
7)Line 242, standardize the usage of amino acid abbreviations.
8)Line 253-275, in fact, this section is largely redundant with "MED15A enhances SA-binding affinity of NPR1", as both try to address the cooperative nature of the tri-molecular complex (NPR1-SA-MED15AKIX) formation.
9)Line 290, please italicize 'PR'.
10)Line 301, 'the NIMIN1 fragment', is it amino acid 36-68? Please briefly note it.
11)Line 350-352, it is recommended that the authors revise these sentences, as the description 'classical plant hormones' is not proper when comparing SA with auxin, JA, and GA. Meanwhile, they are somewhat redundant in content.

Referee #4

(Remarks to the Author)
I co-reviewed this manuscript with one of the reviewers who provided the listed reports.

Referee #5

(Remarks to the Author)
The authors have thoroughly addressed the comments from all reviewers. I recommend the manuscript for publication and congratulate the authors on their work.

Version 2:

Reviewer comments:

Referee #3

(Remarks to the Author)
The revisions have addressed my concerns. I recommend the manuscript for publication.

Open Access This Peer Review File is licensed under a Creative Commons Attribution 4.0 International License, which permits use, sharing, adaptation, distribution and reproduction in any medium or format, as long as you give appropriate credit to the original author(s) and the source, provide a link to the Creative Commons license, and indicate if changes were made.
In cases where reviewers are anonymous, credit should be given to 'Anonymous Referee' and the source.
The images or other third party material in this Peer Review File are included in the article's Creative Commons license, unless indicated otherwise in a credit line to the material. If material is not included in the article's Creative Commons license and your intended use is not permitted by statutory regulation or exceeds the permitted use, you will need to obtain permission directly from the copyright holder.
To view a copy of this license, visit https://creativecommons.org/licenses/by/4.0/

Point-to-Point Responses

Referees' comments:

Referee #1 (Remarks to the Author):

Salicylic acid (SA) is a plant defense hormone that plays a critical role in establishing local and systemic resistance. NPR1 functions as a receptor for SA to positively regulate SA-induced immune gene expression, whereas NIMIN1 is an NPR1-interacting protein that acts as a transcriptional repressor. Despite its established roles in plant immunity, the molecular mechanisms by which NPR1 modulates SA signaling remain incompletely understood. By leveraging AlphaFold predictions, this study identifies the KIX domain of MED15A as a specific NPR1-binding partner, a component which was previously shown to be genetically important for SA signaling. This interaction is validated biochemically and found to be SA-dependent. The authors further determine a cryo-EM structure of NPR1 bound to the MED15A KIX domain, revealing that the KIX domain binds exclusively to the SA-binding domain of NPR1 (NPR1-SBD). Using hydrogen-deuterium exchange mass spectrometry (HDX-MS), they show that SA binding robustly stabilizes the SA-binding core (SBC) within NPR1-SBD, elucidating the mechanism underlying SA-induced NPR1–MED15A-KIX interaction. They also solve the crystal structure of MBP-fused NPR1-SBD in complex with NIMIN1. Structural comparison reveals that NIMIN1 not only sterically occludes the MED15A-KIX binding site on NPR1-SBD but also remodels the SA-binding pocket. Together, these findings demonstrate that SA-bound NPR1 and the Mediator complex together function as a transcriptional co-activator, filling a critical gap in our understanding of SA-induced immune signaling and representing a significant advance in the field of plant immunity. The experiments were well performed, the data are solid, and the conclusions are well supported. The manuscript is clearly written and is a strong candidate for publication in Nature.

We appreciate the time and effort the reviewer spent on reviewing our manuscript and sincerely thank the reviewer for these positive and enthusiastic comments on our findings.

Major comments:

1. As mentioned in the introduction, the NPR1 paralogs NPR3 and NPR4 also function as SA receptors but negatively regulate SA-responsive genes. Based on the structural insights into the NIMIN1-NPR1 interaction, can the authors predict and test whether NIMIN1 interacts with NPR3 and NPR4? NPR3, NPR4, and an NPR4 mutant (R419Q) that loses SA binding but retains the ability to interact with TGA2 and form homodimers have been successfully purified (Ding et al., Cell, 2018). It would be straightforward to test these interactions.

We thank the reviewer for raising this important point. We have indeed predicted and tested the potential binding of NIMIN1 to NPR3 and NPR4 and how their interactions respond to SA. As shown below, the AlphaFold predictions suggest that both NPR3 and NPR4 can interact with NIMIN1 via their SBD domains (Figure R1a). Using purified recombinant proteins, we were able

to further validate their direct interactions (Figure R1b, lane 2, 6, & 10). [redacted] Besides NIMIN1, the *Arabidopsis thaliana* genome also encodes two additional NIMIN1 paralogs, NIMIN2 and NIMIN3. These NIMIN1 paralogs also show differential binding and SA sensitivity for the three NPR paralogs. We are now in the process of systematically analyzing their interactions both in vitro and in vivo.

Unlike NPR1, which positively regulate SA-induced expression of defense genes, NPR3 and NPR4 have been reported to repress gene expression (Ding et al. Cell 2018/Ref. 11). How the NIMIN proteins participate and regulate the activity of NPR3 and NPR4, possibly by recruiting TOPLESS and TOPLESS-like proteins, represents an important question to be thoroughly dissected at the mechanistic level. Because of the NPR1-focused scope of this current manuscript and the complexity of the interactions among NPR3, NPR4, and the three different NIMIN paralogs, we decide to save these NPR3/4-related results for a separate study, which we will prepare a manuscript for publication after we complete our mechanistic investigation. We, nevertheless, thank the reviewer for suggesting these experiments.

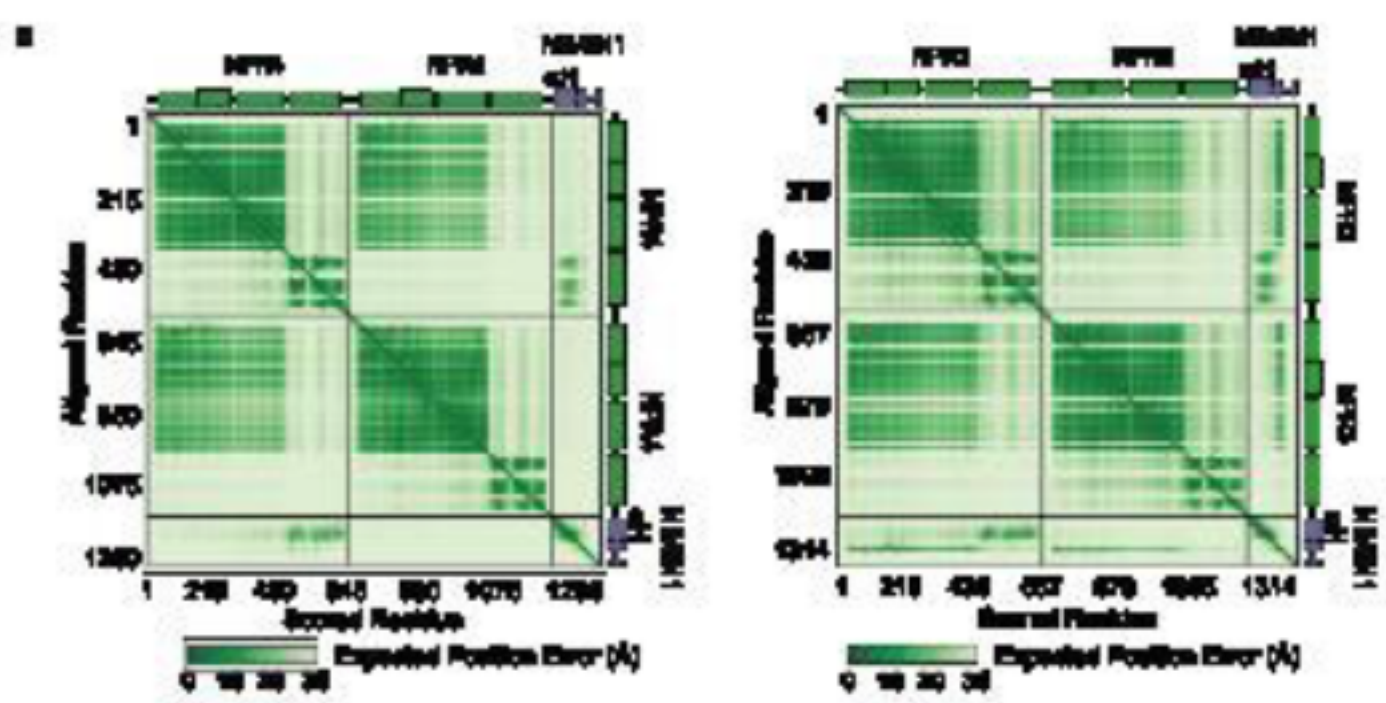

[Figure panel redacted]

Figure R1. a) AlphaFold predictions of the NIMIN1-NPR4 and NIMIN1-NPR3 interactions. [redacted]

2. ITC was used to quantify the SA-binding affinity of NPR1 in the presence of the MED15A-KIX domain. However, under these conditions, the measured heat signal arises from both SA binding to NPR1 and the subsequent binding of MED15A-KIX to SA-bound NPR1, rather than SA binding alone. This complicates the interpretation of the apparent affinity.

We wholeheartedly agree with the reviewer that we need to be cautious when interpreting the apparent affinity of SA toward NPR1 in the presence of MED15A-KIX determined by ITC.

Different from the conventional binary interaction, the ITC measurement of a ternary interaction system results from the collective heat changes of two binding events, the NPR1-SA interaction and the NPR1-MED15A-KIX interaction, which take place in a cooperative manner. We have accordingly revised the sentence to “Remarkably, inclusion of MED15A-KIX enhanced the *‘apparent SA-binding affinity’* of NPR1 to ~ *119 nM*, *which reflects the cooperative engagement of the ligand and the binding partner with the hormone receptor*.”

Minor comments:
1. Lines 149–150: “inclusion of MED15A-KIX enhanced the SA-binding affinity of NPR1 to ~100 nM (Fig. 2e)” The actual value in Fig. 2e is ~119 nM and should be corrected accordingly.

We apologize for the oversight and have now made the correction as “~119 nM”.

2. Line 221: “CTA helix is simultaneously engaged with the αK1 and αK2 helices of MED15A-KIX: This should read “αK1 and αK3 helices,” based on the structural data.

We thank the reviewer for catching this error. We have made the correction accordingly.

3. Line 418: “full-length MED15C” — this should likely be “full-length MED15D,” as MED15D is the isoform discussed in the relevant context.

Yes. This mistake has been corrected. Thank you!

Referee #2 (Remarks to the Author):

Using AlphaFold predictions, the authors identified the Mediator component MED15A as a candidate protein interacting with the salicylic acid (SA) receptor NPR1. MED15A was genetically found as a crucial SA signalling component and part of the NPR1 proxitome before. In this study, in vitro pull-down assays, bio-layer interferometry and AlphaScreen assays revealed that SA increases the binding affinity between NPR1 and the N-terminal KIX domain of MED15A. This result was validated in vivo by bimolecular fluorescence complementation (BiFC) assays. With isothermal titration calorimetry and radio-labelled ligand binding assays, the authors additionally demonstrated that the presence of MED15A-KIX enhances the SA-binding affinity of NPR1. An electrophoresis mobility shift assay showed the in vitro formation of a TGA3-NPR1-MED15A KIX complex being formed at the TGA3 target elements of the PR1 promoter.

The authors performed cryo-electron microscopy of SA-bound NPR1 in complex with the MED15A KIX domain as well as hydrogen-deuterium exchange mass spectrometry to show cooperative binding of SA and MED15A-KIX to NPR1.

In the last section of this manuscript, the molecular function of the NPR1-interacting protein NIMIN1, a known transcriptional repressor was analysed. The crystal structure of the NPR1 SA-binding domain (SBD, fused to the maltose binding protein) in complex with a NIMIN1 fragment, AlphaScreen-based competition assays and BiFC analyses provided evidence for an inhibition of the NPR1-MED15A-KIX interaction by NIMIN1. Radio-labeled ligand binding assay demonstrated that SA-binding to NPR1 is abrogated in the presence of the NIMIN1 peptide. Altogether, the authors presented a model consisting of an SA-bound NPR1 dimer attached to two TGA3 homodimers and two MED15A-KIX domains bridging to the Mediator complex for SA-induced transcriptional activation of NPR1/TGA-dependent target promoters.

It was so far unknown how binding of SA to NPR1 enhances its transcriptional activation capacity. Here, Zhang et al. present comprehensive analyses of the structure and the cooperative binding kinetics of a complex consisting of NPR1, SA and the MED15A KIX domain. I have to admit, that I am not a structural biologist, so it is difficult for me to judge e.g. the quality of the cryo-EM and crystallisation data, but I think the paper is of high interest in the plant-pathogen field.

We thank the reviewer for carefully reviewing our manuscript and offering constructive feedback.

However, I have some remarks:

Figure 1c: NPR3 and NPR4 fusions migrate a little bit slower than NPR1. Why has the pull-down western image been cut directly above the NPR1 band? Most likely, there are no or just faint signals but the upper border of the red box on the full-size membrane might hide signals. How was the treatment with salicylic acid done, which concentration has been used?

We thank the reviewer for pointing out this oversight. We have now re-cropped the gel image to include more upper space both in the main figure and in the uncropped image to address the reviewer's concern. In all the in-vitro pulldown experiments, proteins were expressed and purified separately in buffers that did not contain any SA. For the SA-treated samples, SA was added to 200 μM in the buffer throughout the pull-down experiments, including sample incubation, resin equilibration and wash. We have included these details in the Methods section.

Figure 1d: faint NPR1 band obtained with MED15D FL. Lane 110: Why has the MED15D-FL protein been used, why not MED15A-FL? In the original pull-down western, a faint NPR1 band seems to be visible.

We greatly appreciate the reviewer's sharp and careful attention to detail. There is indeed a very faint band obtained with MED15D FL, although the intensity of this band does not change when SA is present, suggesting that the weak interaction might be non-specific. Taking this into consideration, we have rephrased the sentence as "*Despite these similarities, MED15E and MED15D showed little to no interaction with NPR1 in the presence or absence of SA.*"

We realize that we have omitted in our original manuscript some details about MED15D, which is distinct from MED15A and MED15E by featuring two predicted KIX domains. The first KIX domain close to the N-terminus of the protein shares higher sequence homology to the MED15A KIX domain. This is the reason why we included MED15D FL in our experiment. We have now added a sub-sentence to the text, which reads "Although MED15E shares greater sequence similarity with MED15A, MED15D harbors two tandem KIX domains, the first of which is more homologous to MED15A-KIX."

For a different reason, which was raised by Reviewer 3, we have now also included new data demonstrating that purified NPR1 not only directly interacts with the KIX domain of MED15A, but also MED15A in its full-length form (new Extended Data Fig. 1h). Similar to MED15A-KIX, the interaction between MED15A-FL and NPR1 is also strengthened by SA. These results rule out the possibility that the NPR1-MED15A-KIX domain interaction is an artifact of the truncated form of MED15A.

Lane 121: "screens8,25" has to be corrected to screens18,25 (wrong reference)

We thank the reviewer for catching this mistake and have included multiple citations relevant to the statement. Include 8, 11, 18, 25.

Lane 156 and lane 445: a control for TGA3/NPR1/MED15A-KIX without SA is missing for the statements "SA-bound NPR1 links Mediator to TGA-DNA" and "SA-mediated recruitment of MED15A-KIX to......"

Once again, we appreciate the reviewer's attention to detail. We have performed the experiment suggested by the reviewer, which is shown below. Determined by the DNA-TGA3 and TGA3-NPR1 intrinsic binding affinities and the sensitivity of the EMSA assay, we had to mix 600 nM TGA3 and 15 μM NPR1 with 100 nM dsDNA in order to detect clear and complete consecutive supershifts of the DNA band (Figure R2, lane 1, 2, 3, & 4). To further monitor the upshift of the TGA-NPR1-bound DNA band by MED15A, we had to include the KIX domain of MED15A at a concentration (15 μM) equal to NPR1. Not surprisingly, at such a high concentration, MED15A-KIX by itself starts to shift the TGA-NPR1 associated DNA, but not free DNA, band in the absence of SA (Figure R2, lane 5 and 6) due to the basal weak affinity between MED15A-KIX and NPR1. Such a weak interaction was detected in our affinity pull down and BLI experiments (Figure 1d and 1e).

The overarching goal of this EMSA experiment is to demonstrate that the MED15A-KIX-NPR1 interaction is compatible with NPR1-TGA3-DNA complex assembly. Because we have presented, in the prior sections leading to this EMSA experiment, a series of results establishing the SA-dependent interaction between NPR1 and MED15A-KIX at physiological concentration in the nanomolar range, we prefer to leave out this control, which is expected to confuse non-experts. To be more precise and rigorous, however, we have revised the concluding sentence in the main text to "We, therefore, conclude that *SA-bound NPR1 can bridge MED15A and DNA-bound TGA3*." We have also changed the figure legend to "*Assembly of as-1*-anchored TGA3-

MED15A complex by NPR1 but not NPR4 *in the presence of SA* as assessed by EMSA." Nevertheless, we seek advice from the reviewer regarding whether we should include the control and provide more detailed explanation in the figure legend.

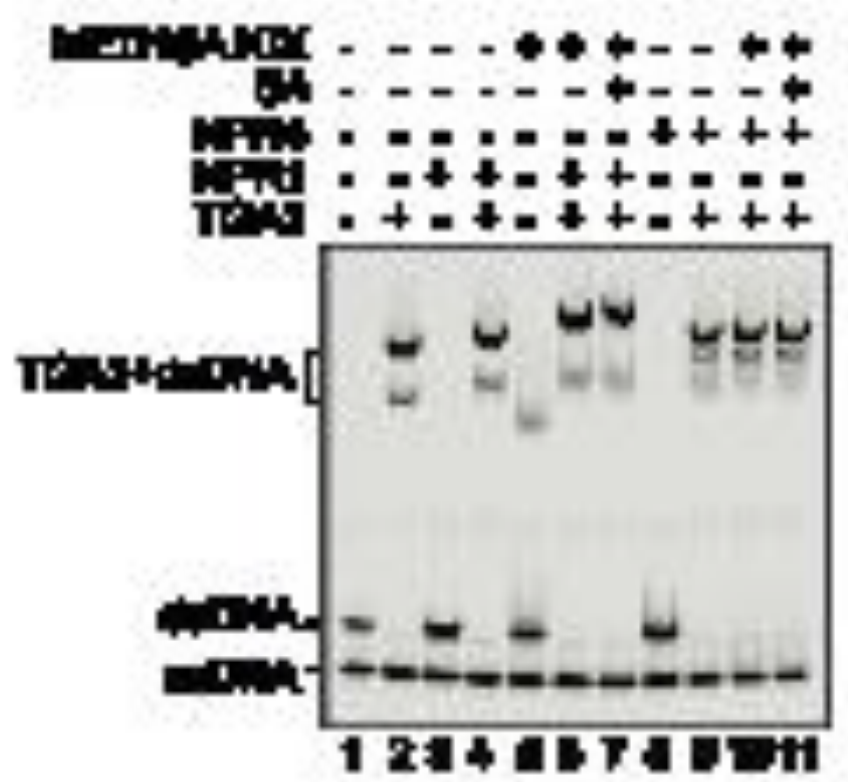

Figure R2. Assembly of *as-1*-anchored TGA3-MED15A complex by NPR1 but not NPR4 in the presence of SA as assessed by EMSA.

Lanes 327,328: NPR1+MED and NPR1+MED in the presence of NIMIN1 hasn't been performed within one experiment.

We would like to clarify that the BiFC assays on NPR1+MED15A and NPR1+MED15A+NIMIN1 in the absence and presence of SA were actually performed within the same experiment, even though the results were plotted in different graphs (Figure 2b, 2c & Figure 5h, 5i) to avoid repetitive graphs. We apologize for the confusion and have added one sentence in the figure legend of Figure 5 to clarify this: "*These BiFC assays were performed in the same experiment described in Figure 2*."

Lane 431: Stars in Figure 2a?

We thank the reviewer for catching our oversight on the asterisks, which indicate MED15-associated bands that are nonspecifically labeled by the anti-NPR1 antibody. To better illustrate the nature of these bands, we repeated the experiments and included more negative controls. The results are shown in the new Extended Data Figure 1g. We have also added the annotation of the asterisks in the figure legend.

Lane 434: BiFC assays with ASK1 are just quantified in Figure 2c, corresponding images are shown in Extended Data Fig. 1g.

We have now referred original Extended Data Fig. 1g, which is now Extended Data Fig. 1i, in the figure legend of Figure 2c.

Lane 435: Avoid "endogenous". RFP is an internal marker serving as control for transformation

and expression efficiency.

We thank the reviewer for the constructive suggestion. We have replaced the word “endogenous” with “internal”.

Lane 489ff, Figure 5: size of the NIMIN1 αH peptide (short?) is missing

We have now included the detail for the peptide, which is “NIMIN1-αH short (residues 36-68)”, to indicate its size in Fig. 5f and 5g. We also added peptide details to Extended data Fig. 3e and 4c, as well as in the Methods section related to the in vitro pull-down assays.

Lane 548: Extended Data Fig. 3: The mirror plot is a perfect mirror image. The “+SA data points are copied from Figure 4a.

We thank the reviewer for catching this oversight. The mirror plot for the + SA & MED15A-KIX data has been corrected and updated.

Lane 741: MED15D-FL? In Figure2b, MED15D-KIX is indicated.

We again appreciate the reviewer’s attention to detail. We did use MED15D-FL, instead of MED15D-KIX, in the BiFC experiment. We have revised Figure 2b to correct the mistake.

Lanes 741, 742: ASK1 has not been used for the in vitro pull-down.

The reviewer is correct. We have revised the sentence in the Methods section to be more precise.

Figure 3d: two αSC1 label

We thank the reviewer for catching this error. One label is now removed.

Extended Data Fig. 3f: Why not 50 µM SA as used in Figure 1e?

The two experiments were performed separately. Based on our AlphaLISA results (Fig. 1g), the binding between NPR1 and MED15A-KIX was already saturated at 10 µM SA, which prompted us to use 10 µM SA in subsequent experiments, including the one described in Extended Data Fig. 3f. To ensure that the same conclusion holds at higher SA concentration, we repeated the experiment with 500 µM SA. As shown below in Figure R3, the results with SA at higher concentration are similar to that obtained with 10 µM SA, in which MED15A-KIX and NPR1-SBD show barely detectable interaction.

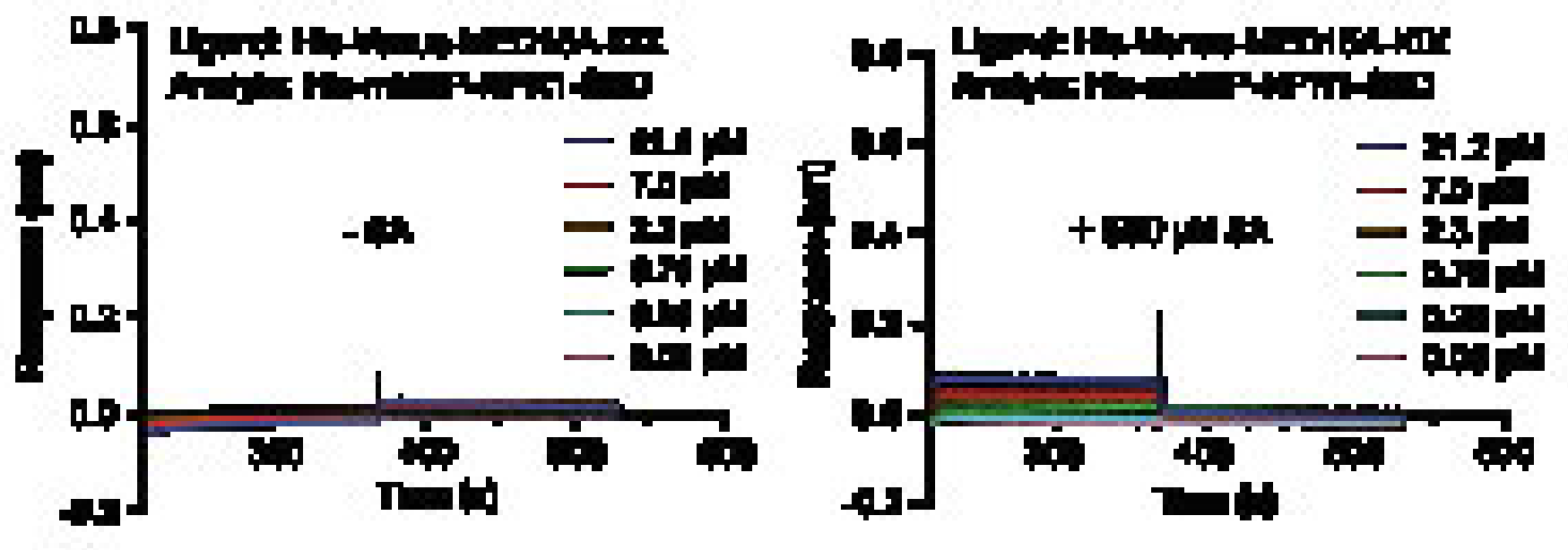

Figure R2. Lack of interactions between isolated NPR1-SBD and MED15A-KIX in the absence or presence of SA as determined by BLI.

Figure 4g: less input protein for the mutated NPR1s

We appreciate the reviewer's rigor and have repeated the experiment to ensure that each NPR1 mutant was present at levels equivalent to the wildtype protein in the input. We have replaced Figure 4g with the new results, which are consistent with and confirm the original conclusion.

Extended Data Fig. 4b: BTB-ANK input amount low

We appreciate the reviewer's rigor and have repeated the experiment to ensure that BTB-ANK was present at a level equivalent to the full-length NPR1 protein in the input. We have replaced Extended Data Fig. 4b with the new results, which are consistent with and confirm the original conclusion.

The manuscript contains many sloppy errors and spelling mistakes, especially in the material and methods section. Just a few examples: Lane 97 "Polymerase" instead of polymerase. Lane 278: MED15A instead of "MED15". Lane 516 "plot" instead of plots. Lane 752 "QiaGen" instead of Qiagen. Lane 650: Sentence "As His-Venus-tagged NPRs.....". Lane 682: "AlphaSreen". Lane 749 "For ASK1, used XbaI and PstI." Lane 794 "petal" instead of pestle.
Not all abbreviations are explained (e.g. lane 608, "mMBP" or lane 613, "Msb")

We sincerely appreciate the reviewer's thoroughness in reviewing our manuscript. We admit that the manuscript was prepared in a rush before the holidays. We have combed through the text and made all the necessary corrections. We have also defined mMBP and Msb/msyB in the Methods section and added a reference (Su et al. 2007/Ref. 53).

Referee #3 (Remarks to the Author):

MED15A, a recently identified proximal component of NPR1, plays an indispensable role in mediating NPR1-dependent transcriptional activation within the SA signaling pathway. In addition, NIMIN1 has long been known to associate with NPR1 and negatively regulate SA responses. In this study, Zhang et al. demonstrate that the ~~the~~ KIX domain of MED15A directly

interacts with NPR1 in an SA-dependent manner and provide structural insights into this interaction. Furthermore, they structurally elucidate the NPR1–NIMIN1 interaction, which contributes to a better understanding of its antagonistic role in NPR1-mediated SA signaling. The current manuscript has several issues that need to be addressed.

We sincerely thank Reviewer #3 and #4 for their efforts in reviewing our manuscript and identifying areas for improvement.

Major points

1.In this study, the authors only show interaction between NPR1 and the isolated KIX domain of MED15A. However, it is the full-length MED15A—not the KIX fragment—that functions in planta. Thus, all subsequent analyses based solely on the KIX domain remain questionable. Moreover, potential artifacts introduced by using only the KIX domain cannot be excluded. Therefore, the interaction and structural analyses between NPR1 and MED15A should be verified using full length of MED15A protein.

We appreciate this comment from the reviewers and have now included data (new Extended Data Fig. 1h) showing that, similar to the isolated KIX domain of MED15A, the full-length MED15A protein can bind NPR1 in an SA-sensitive manner. We would like to point out that there are two important pieces of evidence that prompted us to focus on MED15A-KIX in our studies. First, AlphaFold3 predicts that the NPR1-interacting region is exclusively located to the KIX domain of MED15A (Figure 1b). Second, all three missense mutations found in the *nrb4* alleles were mapped to the MED15A KIX domain (Canet et al. 2012/Ref. 22). Based on AlphaFold3 prediction, the majority of the full-length MED15A protein sequence is intrinsically disordered (Figure 1b). This feature, unfortunately, makes it infeasible to determine the structure of full length MED15A in complex with NPR1.

2.The E90K mutation is a known loss-of-function, SA-insensitive allele identified in previous genetic screens. Although MED15A-KIX-E90K shows reduced interaction with NPR1 compared with wild-type MED15A-KIX, it still associates with NPR1 in vivo upon SA treatment (Fig. 2b). This observation is inconsistent with previous genetic evidence and the in vitro interaction data shown in Fig. 1f, and therefore requires further clarification.

We thank the reviewers for raising this issue for discussion. We would like to clarify that the in vitro affinity pull-down assay described in Fig. 1f, the BiFC assay described in Fig. 2b, and the genetic phenotypic analysis previously reported by Canet et al. 2012 (Ref. 22), are three distinct experiments, in which the proteins of interest, MED15A-KIX-E90K and NPR1, were investigated at different levels and under different conditions. Although a single missense mutation in a protein of interest can often impair protein-protein interactions, resulting in a clear loss-of-function phenotype, it does not necessarily have to completely obliterate binding to a non-detectable level, especially when the protein of interest and its binding partner are expressed at a high level. In other words, a reduction of affinity by 100 folds (e.g., from 10 nM to 1 μM) can be sufficient to impact protein-protein interaction and lead to a clear genetic phenotype when the two endogenous proteins of interests are expressed at 10-100 nM. But their weakened

interactions could still be detected when both proteins were exogenously overexpressed at 1-10 μM in a different experimental setting. For the same reason, similarly weakened protein-protein interactions could be undetectable in a stringent in vitro pull-down assay, which involves multiple steps of wash (non-equilibrium), yet remain detectable under an equilibrium condition as seen in the cell (BiFC).

In Fig. 2b, because both NPR1 and MED15A-KIX were exogenously overexpressed under the 35S promoter, both the basal affinity between NPR1 and wildtype MED15A-KIX without SA and the compromised interaction between NPR1 and MED15A-KIX-E90K that is enhanced by SA can be detected. However, their interaction signals are substantially below the SA-elevated binding between NPR1 and wildtype MED15-KIX.

3.It appears that only one MED15A KIX domain shows electron density in the 2:2 symmetric NPR1-SA-MED15A-KIX dimer claimed by the authors (Ext Fig 2a). Additionally, the authors performed chemical cross-linking on the purified NPR1-SA-MED15A-KIX complex (Line 857). Therefore, the 2:2 dimeric complex is largely inferred by the authors (Fig 3a), clarification on this point is requested.

We would like to point out that the 2:2 symmetric nature of the NPR1-SA-MED15A-KIX dimer can be visualized by one of the representative 2D class averages shown in Extended Figure 2c. To better illustrate this, we show below (Figure R3) a comparison between a representative 2D class average of the free NPR1 dimer published by Kumar et al. 2022 (Ref. 13) and that of the NPR1-SA-MED15A-KIX dimer shown in Extended Figure 2c. As discussed in the main text, the density of the NPR1 SBD is largely invisible unless it is stabilized by MED15A-KIX in the presence of SA. Obviously, such a 2:2 dimer could lose one or both copies of MED15A-KIX during cryo-EM grid preparation, which might explain the heterogeneity of 2D class averages.

Chemical cross-linking is now a common practice to stabilize protein complexes for cryo-EM grid preparation because protein assemblies tend to fall apart at the water-air interface. To our knowledge, this approach has not been found to create artificial protein-protein interactions that have been captured by cryo-EM single particle reconstruction. This is not surprising as non-specific protein crosslinking is not expected to yield hundreds and thousands of particles with the similar protein binding mode. Therefore, the 2:2 dimeric NPR1-SA-MED15A-KIX complex is unlikely the result of a cryo-EM artifact.

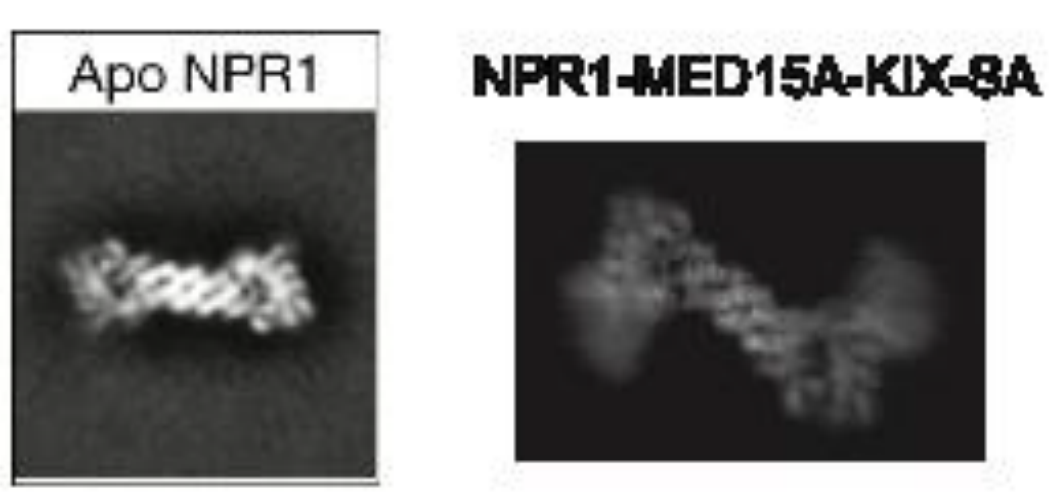

Figure R3. A 2D class average comparison between apo NPR1 dimer (Kumar et al. 2022/Ref. 13) and the MED15A-KIX-SA-bound NPR1 dimer.

4.The A61V mutation in MED15A has been reported previously to disrupt MED15A function. However, the structural analyses presented here do not provide mechanistic insights into how this mutation impairs MED15A activity (Lines 236–240).

We thank the reviewers for flagging the lack of clarity on this mutant. We have now added one sentence to explain the mechanism by which the A61V mutation impacts MED15A activity – "*The resulting bulkier side chain is expected to compromise the proper folding of the compact three-helix bundle*". We also realize that we mislabeled the A61 residue as "A60" in Figure 3e & 3f, which might have contributed to the confusion. We have now made the correction and referred to these two figure panels when describing the A61V mutation.

5.Structural analyses indicate that MED15A-KIX does not contact the ANK domain of NPR1. It is therefore unclear why the isolated NPR1 SBD domain fails to interact efficiently with MED15A-KIX in the presence of SA when the ANK domain is absent.

Our NPR1-MED15-KIX-SA complex structure reveals that NPR1-SBD interacts with MED15A-KIX and NPR1-ANK via two separate interfaces. Our current studies and previous analysis published by Kumar et al. (Ref. 13) indicate that both of these inter-domain interactions rely on SA binding to NPR1-SBD and, at the same time, contribute to stable ligand engagement. Therefore, the interactions among the four components, i.e., SA, NPR1-SBD, NPR1-ANK, and MED15A-KIX, are highly cooperative. The lack of any component in this interacting network, e.g., ANK, will compromise the interactions among the rest of the system. We recognize that the complexity of this system may pose an initial conceptual challenge, but we believe it is this very property — an unexpectedly cooperative four-component network — that constitutes the novelty and elegance of the SA perception mechanism.

6.To elucidate the NPR1–NIMIN1 interaction, it would be preferable to use full-length NPR1 and NIMIN1 proteins, rather than relying solely on an NPR1 SBD domain and a truncated NIMIN1 fragment.

We agree with the reviewers that a structural study should ideally include full-length proteins to possibly capture the complete picture of protein-protein interaction. However, similar to the majority region of MED15A (Point 1), the sequence regions of NIMIN1 flanking the central α-helix are predicted to be intrinsically disordered by AlphaFold. Furthermore, unlike in the complex formed between NPR1 and MED15A, NPR1-SBD in the NPR1-NIMIN1 complex is predicted to be structural uncoupled with from the rest of the protein (Figure R4a vs. R4b). Such decoupling between NPR1-SBD and NPR1-ANK can be explained by the NIMIN1-induced remodeling of NPR1-CTA helix, which would sterically clash with NPR1-ANK (Fig. 5e). Therefore, the NPR1-SBD-NIMIN1-αH module is expected to be flexibly tethered to the rest of the NPR1 dimer, making it invisible upon cryo-EM 2D class averaging. In fact, this is exactly what we have seen when we tried to determine the cryo-EM structure of full-length NPR1 in complex with full-length NIMIN1.

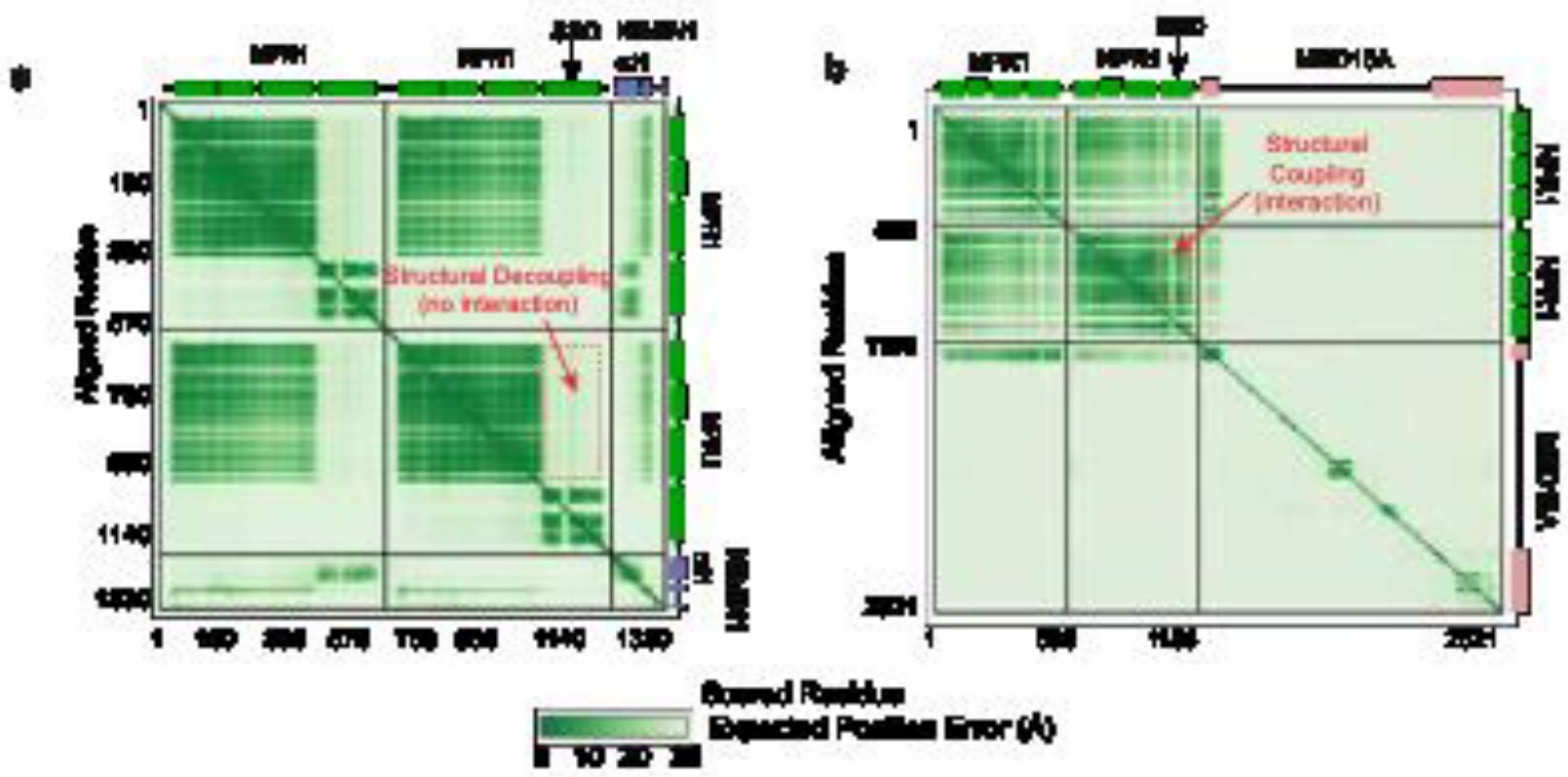

Figure R4. A comparison of the PAE plots of the NPR1-NIMIN1 interaction and the NPR1-MED15A interaction (from main Figure 1b) generated by AlphaFold. The off-diagonal blocks outlined by red dashed lines reflect the spatial relationships between the SBD and the BTB-ANK domains within one NPR1 protomer. Dark green color corresponds to small “expected position error”, which are indicators of structural interactions.

7.Since SA disrupts the NPR1-NIMIN1 interaction, the NPR1-MED15A interaction would be expected to be enhanced upon SA treatment in the BiFC assays (Fig. 5h, i), even in the presence of NIMIN1 co-expression. This point requires clarification or additional experimental support.

We thank the reviewers for raising this interesting question. In Figure 5h, i, we show that SA disrupts NPR1-NIMIN1 interaction when YFP^{N}-NPR1 was co-expressed with NIMIN1-YFP^{C}. To investigate the effect of NIMIN1 on the interaction between NPR1 and MED15A-KIX, free NIMIN1 was overexpressed together with YFP^{N}-NPR1 and MED15A-KIX-YFP^{C}. In these two experiments, the protein levels of NIMIN1, one in the form of YFP^{C}-fusion and the other as a free protein, could be significantly different. We speculate that the free NIMIN1 was expressed at a high enough level that SA cannot effectively block its binding to NPR1.

8.In the BiFC assays, NIMIN1 should competitively bind to YFPN-fused NPR1 against YFPC-fused NIMIN1, thereby resulting in a weaker interaction signal compared to when NIMIN1 is not co-expressed with YFPC-fused NIMIN1 and YFPN-fused NPR1. However, the quantitative interaction signals presented in Fig. 5i appear to contradict this assertion. It is requested to either clarify this discrepancy or provide complementary in vivo interaction assays to validate their findings.

This is yet another interesting raised by the reviewers. When NIMIN1 is co-expressed with YFP^{N}-NPR1 and NIMIN1-YFP^{C}, the interaction signal between YFP^{N}-NPR1 and NIMIN1-YFP^{C} is only slightly reduced compared to when NIMIN1 is absent. In both cases, however, the interaction between YFP^{N}-NPR1 and NIMIN1-YFP^{C} is sensitive to SA, which is consistent with the competition between SA and NIMIN1 for binding NPR1. One plausible reason for the lack of significant interaction signal reduction when NIMIN1 is co-expressed is that NIMIN1-YFP^{C} is retained in the same protein complex assembly when it is replaced by free NIMIN1. As a repressor, NIMIN1 is characterized by a C-terminal EAR motif (Weigel et al. 2005/Ref. 21),

which is a well-known TOPLESS (TPL)- and TPL-related protein (TPR)-binding motif found in different transcription repressors in plants. Previous structural studies have revealed that TPL and TPR transcription co-repressors form stable homo-tetramers, which harbor four EAR motif binding sites (Ke J et al. 2015 Sci. Adv. E1500107; Martin-Arevalillo R et al. 2017 PNAS 114:8107-8112). It is, therefore, conceivable that hetero-oligomers might form among TPL/TPRs, NIMIN1- YFP^C, and YFP^N-NPR1-NIMIN1 after NIMIN1 competitively binds to YFP^N-NPR1. Within these hetero-oligomers, the BiFC signals would not substantially diminish due to the spatial proximity between NIMIN1- YFP^C and YFP^N-NPR1-NIMIN1. As mentioned in our response to the first major point raised by Reviewer 1 (see above), we are actively investigating the complex assemblies formed among members of the NPR, NIMIN, and TPL/TPR protein families. We will report our findings on this topic in a separate study.

9.It is recommended that the physiological functions of the interaction interfaces within the NPR1-MED15A KIX and NPR1-NIMIN1 protein complexes should be validated by introducing targeted point mutations at the interfaces, followed by transcriptional activity assays in plants or phenotypic observation in transgenic plants.

We thank the reviewers for the suggestions. If we had identified novel protein interactors for the first time and they had not been characterized by mutational analysis, we would have definitely validated the structures by introducing structure-guided point mutations at the protein-protein interfaces, followed by biochemical and functional assays. In this case, however, MED15A was already identified by the Tornero group (Canet et al. 2012/Ref. 22) in a genetics screen and three mutant alleles were found that are specifically defective in SA signaling. As discussed in our manuscript, two of the missense mutations (E66K, E90K) are precisely mapped at the NPR1-MED15A-KIX interface and the third resides in the hydrophobic core of the MED15 KIX domain (A61V). In our opinion, they represent compelling evidence in support of the structure. In congruence with their common SA-insensitive phenotype, we further validated their negative impact on NPR1-MED15-KIX interaction (Figure 1f). Moreover, the Tornero group has also published a series of SA-insensitive NPR1 mutant alleles (Canet et al. 2010/Ref. 29), which involve missense mutations mapped at the interfaces between NPR1-SBD and MED15A-KIX, NPR1-SBD and SA, as well as NPR1-SBD and NPR1-ANK. One of these mutants is NPR1 R544K, which is located at the NPR1-SBD-MED15A-KIX interface, as highlighted in our manuscript. Together, these mutations provide additional evidence validating our structure. Lastly, we are aware of a manuscript co-submitted by Dr. Yuelin Zhang's group at the University of British Columbia, in which the functional defects of a MED15A mutant carrying a missense mutation at the NPR1-interacting interface and the NPR1 R544K mutant have been characterized in a transcription activity assay. Collectively, these data obtained by different research groups all help validate the physiological importance of the NPR1-MED15A-KIX interaction interfaces.

In the case of NIMIN1, the Pfitzner group has published a series of papers characterizing the structure-function relationship of the protein using yeast two hybrid- and co-IP-based protein-protein interaction assays, SA-induced gene expression reporter assay, and pathogen resistance phenotypic analysis. These studies have identified the tandem

phenylalanine motif of NIMIN1, which is mapped at the center of the NPR1-NIMIN1 interface in our structure. Their results are highlighted in our description of the NPR1-NIMIN1 complex structure.

Nevertheless, following the recommendations made by the reviewers, we have selected additional NPR1-SBD, MED15A-KIX, and NIMIN1-αH residues at their interaction interfaces and introduced a series of mutations to further validate the NPR1-MED15A-KIX and NPR1-SBD-NIMIN1-αH structures. In affinity pull-down assays, all these mutations either impaired or completely abolished the binding of NPR1 to MED15A-KIX or full-length NIMIN1. We now include these new data in Extended Data Fig. 2e (NPR1-MED15A-KIX interactions) and Extended Data Fig. 4c (NPR1-SBD-NIMIN1-αH interactions). In conjunction with the results described above, we believe that the functional relevance of the interaction interfaces unveiled in our structural studies is fully validated.

10.SA is embedded within the center of the NPR1-SBC four-helix bundle (Line 196) and therefore cannot directly contact MED15A-KIX. Hence, referring to SA as a "glue" here is misleading. Although the authors attempt to describe both SA and gibberellin as "intramolecular glues" (Line 359), this description remains highly susceptible to misinterpretation, as readers may assume that SA, much like a conventional "glue", simultaneously contacts both NPR1 and MED15A-KIX to bridge them, whereas in reality SA only directly binds to the receptor NPR1. Therefore, the term "glues" in the title "Salicylic Acid Allosterically Glues NPR1 and Mediator to Activate Plant Immunity" is also recommended to be revised.

The reviewers' point is well taken. We have also been debating among ourselves on whether it is appropriate to raise "allosteric glue" as a concept. We believe that the confusion arises from the different usage of the word "glue". When used in the term of "molecule glue", it refers to small molecules acting at the protein interface, promoting protein-protein interaction. On other occasions, the word "glue" has been casually used in the field of induced proximity to describe compounds that foster protein-protein interaction in general. We wholeheartedly agree with the reviewers that mixing the usage in our story can be misleading. Given that the unique aspect of SA's mechanism of action revealed in our study is its ability to induce the proper folding of NPR1-SBD to engage MED15A-KIX, we have now changed the title to "Salicylic Acid Co-folds NPR1 to Recruit Mediator-15 in Plant Immunity Activation". To a large degree, this mechanism is reminiscent of that of the plant hormone GA, which binds to the C-terminal domain of its receptor, GID1, and induces the engagement of its N-terminal extension domain that is otherwise flexible. This leads to the binding of DELLAs.

Minor points

1.In Fig. 2a, the two slower-migrating bands above the labeled NPR1 in the IP samples are likely non-specific proteins pulled down during immunoprecipitation. However, it is unclear why the abundance of these non-specific bands differs among the MED15A/D/E samples.

The two slower-migrating bands above NPR1 in the IP samples are actually non-specific proteins co-purified with the MED15A/D/E proteins from E. coli, which were labeled by anti-

NPR1 antibody. This is why the abundance of these non-specific bands differs among the samples. To better illustrate their origin, we have repeated the IP experiments by including more control lanes. The new data is shown as Extended Data Fig. 1g.

2.Line 186: “discernible” (spelling).

The typo has been corrected. Thank you!

3.Line 232: “CTH” is introduced without definition. Should this be the C-terminal helical arm (CTA) in Line 194 and the C-terminal arm (CTA) in Line 471?

We thank the reviewer for catching this mistake. “CTH” in Line 232 should be “CTA”, which is defined on Line 194.

Referee #4 (Remarks to the Author):

I co-reviewed this manuscript with one of the reviewers who provided the listed reports.

We appreciate the reviewer for co-reviewing our manuscript.

Referee #5 (Remarks to the Author):

The manuscript by Zhang and coworkers outlines the allosteric mechanism underlying SA binding to its receptor, NPR1, and the concomitant activating effect on MED15A binding. The authors approach this using a series of technically sound and well-designed experiments, starting with pull-down assays and immunoprecipitation of whole-cell lysates to demonstrate this interaction, with appropriate controls including other KIX domain-containing MED15 paralogs and MED15A variants. They further show cooperative binding of SA and MED15A using AlphaScreen competition assays, ITC, and BLI. Cryo-EM structures and HDX-MS analyses provide detailed and convincing structural information characterizing this interaction and explaining the loss-of-function mutants observed in genome libraries. Additionally, the work demonstrates how the negative feedback regulator, NIMIN1, binds NPR1 to displace MED15A. Overall, this study provides mechanistic insight into how SA-mediated gene regulation is facilitated. This is an interesting study that merits publication in Nature once the following minor points are addressed.

We thank the reviewer for the positive comments about our study.

Points to address:
1) “Such an SA-dependent NPR1-interacting protein might also help explain the inconsistent SA-binding activity of NPR1, which varies substantially depending on the methods employed for protein purification and ligand-binding measurement.”

Is there a reference to support this claim?

We have added several references reporting that SA either shows no binding to NPR1 (Fu et al. 2012/Ref. 10) has a measurable affinity to NPR1 with a Kd value ranging from 110 nM to 1.86 μM (Wu et al. 2012/Ref. 12, Ding et al. 2018/Ref. 11, Wang et al. 2020/Ref. 15, & Ji et al. 2022/Ref. 16).

2) "In addition to NIMIN1, which has been shown to directly interact with NPR1"

Please provide a citation for this statement.

We have added two references for this statement (Weigel et al. 2001/Ref. 20; Weigel at al. 2005/Ref. 21).

3) "To identify the missing factor that hypothetically interacts with SA-bound NPR1, we performed AlphaFold predictions of the SA receptor in complexes with individual NPR1-proximal proteins recently revealed by proximity labeling experiments17."

The claim is that NPR1 binds MED15A in an SA-dependent manner. Were these AlphaFold predictions performed with SA included?

This is a great question. The AlphaFold predictions were performed without SA. Despite the lack of the ligand, AlphaFold3 was able to yield converging solutions for the NPR1-MED15A-KIX complex with low "expected position errors" as seen in the PAE plots (e.g., Figure 1b) and high confidence (pLDDT scores, data not shown). Besides physical and chemical properties of the amino acids, AlphaFold utilizes co-evolution information of residues, which is provided by multiple sequence alignments (MSA), to identify interface contacts. This is achieved by mapping evolutionary and spatial relationships between every pair of amino acids from the two target proteins. Importantly, although AlphaFold was able to predict and identify MED15A as a potential direct interactor of NPR1 from its proxiome, we had to turn to wet-bench experiments to establish the SA-dependency of NPR1-MED15A binding.

4) Figure 1G. What is the error associated with the fitted values shown?

The EC_{50} values were obtained from the nonlinear regression fits for the dose response curves of SA and BA in promoting NPR1-MED15A-KIX interaction. We have now included the 95% confidence intervals for the two EC_{50} values in the figure legend.

5) "AlphaFold predictions indicate that the Arabidopsis MED15A CTD engages in analogous interactions with its partners, MED14 and MED23 (Extended Data Fig. 1i)."

What is the confidence level of this prediction? No data are provided to suggest that this is a

robust interaction beyond a visual inspection of the predicted structure.

In Extended Data Fig. 1i, we show the five solutions from AlphaFold predictions of the MED14-MED15A-MED23 complex with only MED15A-CTD being superimposed. The “co-superposition” of the five copies of MED14 and MED23 in the five solutions indicate low “expected position errors” for the MED14-MED15A-CTD and MED15A-CTD-MED23 interfaces. In essence, the converging solutions of the MED14-MED15A-MED23 interfaces represents another way to visualize the confidence level of AlphaFold prediction besides the PAE plots.

The reviewer is correct that, ideally, we should provide experimental evidence to demonstrate the interaction between MED15A and the rest of the Mediator complex. However, this would involve reconstituting the entire plant Mediator complex, which is itself a major endeavor. It is worthwhile mentioning that not only the endogenous Mediator complex isolated from *Arabidopsis thaliana* has been shown to contain MED15A (Chen et al. 2022 Int. J. Mol. Sci. 23:6170), but additional subunits of the plant Mediator complex have also been identified in the NPR1 proxiome (Powers et al. 2024/Ref. 19). To our knowledge, MED15 (including MED15A in plants) has been documented to function almost exclusively as a core subunit of the Mediator complex in all eukaryotic species.

6) Figure 4C - HDX data: The figure scale should be quantitative, showing either ΔDa or Δ% deuterated rather than using qualitative labels such as “more protected.”

The reviewer made a good point. We have updated the legend in the figure to quantitatively show the extent of changes in ΔDa.

7) “The apparent unfolding rate is approximately 10-fold slower with MED15A-KIX bound, further indicating that the MED15A interaction stabilizes the helices within the SBD.”

The authors should show how this 10-fold slower exchange rate was determined using a figure panel.

This is another great suggestion. We have now included an additional panel in Extended Data Fig. 3c that plots the fraction opened across time points for +SA and +SA &MED15 states along with the kinetic fits.

8) Before discussing HDX results, a sentence or two should briefly explain what reduced deuterium uptake means in the context of protein structure to make the section more interpretable to a broad audience. Also please consider briefly expanding on the HDX-MS workflow in the main text, including mention of the continuous-labeling strategy.

We thank the reviewer for the constructive suggestions. We have included a sentence as a brief overview of what HDX-MS measures. Due to space constraints, unfortunately, we have to balance the details of each type of techniques we have used and the overall description of the study in the main text.

9) Please include the HDX summary table as per “Masson, G.R., Burke, J.E., Ahn, N.G. et al. Recommendations for performing, interpreting and reporting hydrogen deuterium exchange mass spectrometry (HDX-MS) experiments. Nat Methods 16, 595–602 (2019). https://doi.org/10.1038/s41592-019-0459-y” The HDX data deposition entry also needs to be updated. It is currently written as XXXXX.

We have included Supplementary Table 1 containing all of the results as well as the summary table in the format that has been suggested by Masson et al. 2019 in their Nat Methods paper. The summary table is in a tab titled “Statistics”. The PRIDE submission accession number has now been included.

10) Given the title, “Salicylic Acid Allosterically Glues NPR1 and Mediator to Activate Plant Immunity,” the concept of SA as a “molecular glue” should be introduced much earlier in the discussion. Additionally, the paper does not provide direct evidence that SA promotes NPR1 interaction with the entire Mediator complex, but rather only with MED15A, which is a component of Mediator. The title should be revised to reflect this specificity more accurately.

We appreciate the reviewer’s suggestion to introduce the concept of “molecular glue” earlier in the Discussion section. In response to the comments made by Reviewer 3 under Point 10, however, we have decided to deemphasize the “glue” aspect of SA to avoid any confusion over the definition of “molecular glue”. We have changed the title and the Discussion section to highlight the unique mechanism of action of SA as a co-folder of NPR1 to recruit MED15A.

As mentioned under Point 5), the current literature suggests that the MED15 orthologs, including MED15A in plants, do not have a physiological function outside the Mediator complex. In the previous study mapping the proxiome of NPR1, several Mediator subunits in addition to MED15A, have been identified, implicating that MED15A was captured when bound to the rest of the Mediator complex. We, therefore, included the term “Mediator” in the original title. Nevertheless, we echo the rigorous treatment suggested by the reviewer and have replaced the term “Mediator” with “Mediator-15”. We have also toned down our claim in the Discussion section to state that “our findings *suggest* that SA instead promotes recruitment of the Mediator complex to NPR1…” instead of “our findings show that SA…”. To better illustrate the nuances of MED15A-KIX vs. the Mediator complex, we added a schematic model of SA signaling through NPR1 to Fig. 5j.

Point-to-Point Responses

Referees' comments:

Referee #1 (Remarks to the Author):

The revision has satisfactorily addressed my previous concerns. I support publication of the revised version of the manuscript in Nature.

We sincerely thank the reviewer for the constructive comments and suggestions made in the last cycle and for supporting the publication of our revised manuscript.

Referee #2 (Remarks to the Author):

The authors have adequately revised the manuscript in response to my comments. For the resubmitted version, I have only a few remaining points:

We are grateful to the reviewer for his/her effort and time spent on reviewing our manuscript.

It is not necessary to include Figure R2, but the statement of the authors in their rebuttal letter "…at such a high concentration, MED15AKIX by itself starts to shift the TGA-NPR1 associated DNA, but not free DNA..." is not correct. Lane 5 (MED15A-KIX alone plus DNA) shows a clear band shift!!!

We appreciate that the observation made by reviewer. However, it is important to point out that MED15A-KIX by itself only shifts a small fraction of DNA in lane 5 of Figure R2, whereas it causes a complete up-shift of the TGA-NPR1-associated DNA band in lane 6. In our multiple times of EMSA trials, we noticed that MED15A-KIX could shift the DNA even in the presence of a high concentration of the non-specific competitor, poly(dI-dC) (up to 500 ng/uL). MED15A-KIX has a high pI of 10.52, whereas all other proteins involved in this experiment have a pI below 7. We believe that the shift caused by MED15A-KIX represents the non-specific interaction between the positively charged MED15A-KIX and the negatively charged DNA in our reaction buffer at pH 7.5.

Despite these observations, our original conclusion that SA-mediated MED15A-KIX binding to NPR1 is compatible with TGA-NPR1 assembly with DNA still holds. In lane 7, the DNA band upshifted by MED15A-KIX alone is invisible, indicating that the non-specific $MED15A^{KIX}$-DNA interaction is negligible when TGA-NPR1-SA is present. We appreciate the reviewer's suggestion to not include Figure R2 in the manuscript to avoid any confusion it might introduce to the general readers.

Lane 538 should be corrected to: As encoded by an SA-responsive gene, NIMIN1.

We have made the correction.

Extended Data Fig. 1h: How did the authors detect Venus fluorescence? A standard SDS-PAGE protocol disrupts the protein's structure thereby causing loss of its fluorescence.

We followed an in-gel florescence detection protocol published by Sanial et al. (Biol Cell 2025, PMID: 39924827). Specifically, the protein samples in 1x sample buffer were not boiled but loaded directly on the SDS-PAGE gel. This allows one to detect the Venus signal without disrupting the structure of the fluorescent protein. The Venus signal was detected under wavelength 488nm (protein ladder at 680nm) prior to Coomassie staining of the gel. After staining and de-staining, the gel was imaged again for Coomassie blue. We have included the technical details in Methods.

Referee #3 (Remarks to the Author):

In this revised version, the authors have provided new data that have basically addressed my previous concerns.

In the title, given that NPR1 specifically interacts with MED15A but not other Mediator subunits (e.g., MED15D/E), it is recommended that the authors substitute 'Mediator-15' directly with 'MED15A'.

We appreciate the reviewer for the specific suggestion. We have decided to change the title to "Transcriptional activation of plant immunity by salicylic acid",

which is more concise and better reflects the mechanism by which NPR1 is dually regulated by SA and NIMIN1.

Additionally, this manuscript still contains some minor errors, examples are shown as below:

1)Line 55, insert ‘the’ before ‘sequence level’.

We have made the correction.

2)Line 59, insert ‘assumed’ before ‘opposing mechanisms’, as the detailed mechanisms by which NPR3/4 modulate transcription have not been elucidated previously, or in this manuscript. Similarly, in line 204.

We have made the suggested edit.

3)Line 63, the full name of TGA.

We have added the full name of TGA.

4)Line 106, pull-down.

We have made the correction.

5)Line 123, please superscript KIX in ‘NPR1-MED15A-KIX’, do not hyphenate it with MED15A.

We have made the suggested change.

6)Line 142-158, I realize that MED15A stabilizes the binding of NPR1 with SA, but the phrasing like ‘recruitment of MED15A-KIX sensitizes NPR1 for binding SA’ may be misleading. It would benefit from some revision, for example: recruitment of MED15A-KIX by SA-bound NPR1 further enhances its SA sensitivity.

We have revised the sentence as suggested.

7)Line 242, standardize the usage of amino acid abbreviations.

Good catch. We have made the correction.

8)Line 253-275, in fact, this section is largely redundant with “MED15A enhances SA-binding affinity of NPR1”, as both try to address the cooperative nature of the tri-molecular complex (NPR1-SA-MED15AKIX) formation.

We appreciate the reviewer’s comment but would like to emphasize that this section, while thematically echoing the prior sections entitled “MED15A enhances SA-binding affinity of NPR1” and “MED15A interacts with NPR1 in an SA-dependent manner”, is focused on using HDX-MS to reveal the structural impact made by SA and MED15A on NPR1-SBD, beyond the quantification of their interactions. The HDX-MS analysis is meant to mechanistically connect the affinity measurements and the cryo-EM structure, which only offers a snapshot of the $MED15A^{KIX}$-NPR1-SA ternary complex assembly. The HDX-MS results allow us to conclude that the binding of SA and MED15A cooperatively stabilizes the structural fold of NPR1-SBD, which is otherwise difficult to assess.

9)Line 290, please italicize ‘PR’.

We have made the correction.

10)Line 301, ‘the NIMIN1 fragment’, is it amino acid 36-68? Please briefly note it.
We have added the annotation.

11)Line 350-352, it is recommended that the authors revise these sentences, as the description ‘classical plant hormones’ is not proper when comparing SA with auxin, JA, and GA. Meanwhile, they are somewhat redundant in content.
We have streamlined the sentence to be more concise and technically accurate.

Referee #4 (Remarks to the Author):

I co-reviewed this manuscript with one of the reviewers who provided the listed reports.
We thank the reviewer for co-reviewing our manuscript together with Referee #3.

Referee #5 (Remarks to the Author):

The authors have thoroughly addressed the comments from all reviewers. I recommend the manuscript for publication and congratulate the authors on their work.

We thank the reviewer for carefully reviewing our manuscript and providing constructive feedback that help strengthen our story.
